# High-Dose Buprenorphine Induction in the Emergency Department for Treatment of Opioid Use Disorder

**DOI:** 10.1001/jamanetworkopen.2021.17128

**Published:** 2021-07-15

**Authors:** Andrew A. Herring, Aidan A. Vosooghi, Joshua Luftig, Erik S. Anderson, Xiwen Zhao, James Dziura, Kathryn F. Hawk, Ryan P. McCormack, Andrew Saxon, Gail D’Onofrio

**Affiliations:** 1 Department of Emergency Medicine, Highland Hospital—Alameda Health System, Oakland, California; 2 Department of Emergency Medicine, University of California, San Francisco, San Francisco; 3 Keck School of Medicine, University of Southern California, Los Angeles; 4 Yale Center for Analytical Sciences, Yale University, New Haven, Connecticut; 5 Department of Emergency Medicine, Yale School of Medicine, New Haven, Connecticut; 6 Ronald O. Perelman Department of Emergency Medicine, NYU Grossman School of Medicine, New York, New York; 7 Department of Psychiatry and Behavioral Sciences, University of Washington, Seattle; 8 Department of Chronic Disease Epidemiology Yale School of Public Health, New Haven, Connecticut

## Abstract

**Importance:**

Emergency departments (EDs) sporadically use a high-dose buprenorphine induction strategy for the treatment of opioid use disorder (OUD) in response to the increasing potency of the illicit opioid drug supply and commonly encountered delays in access to follow-up care.

**Objective:**

To examine the safety and tolerability of high-dose (>12 mg) buprenorphine induction for patients with OUD presenting to an ED.

**Design, Setting, and Participants:**

In this case series of ED encounters, data were manually abstracted from electronic health records for all ED patients with OUD treated with buprenorphine at a single, urban, safety-net hospital in Oakland, California, for the calendar year 2018. Data analysis was performed from April 2020 to March 2021.

**Interventions:**

ED physicians and advanced practice practitioners were trained on a high-dose sublingual buprenorphine induction protocol, which was then clinically implemented.

**Main Outcomes and Measures:**

Vital signs; use of supplemental oxygen; the presence of precipitated withdrawal, sedation, and respiratory depression; adverse events; length of stay; and hospitalization during and 24 hours after the ED visit were reported according to total sublingual buprenorphine dose (range, 2 to >28 mg).

**Results:**

Among a total of 391 unique patients (median [interquartile range] age, 36 [29-48] years), representing 579 encounters, 267 (68.3%) were male and 170 were (43.5%) Black. Homelessness (88 patients [22.5%]) and psychiatric disorders (161 patients [41.2%]) were common. A high dose of sublingual buprenorphine (>12 mg) was administered by 54 unique clinicians during 366 (63.2%) encounters, including 138 doses (23.8%) greater than or equal to 28 mg. No cases of respiratory depression or sedation were reported. All 5 (0.8%) cases of precipitated withdrawal had no association with dose; 4 cases occurred after doses of 8 mg of buprenorphine. Three serious adverse events unrelated to buprenorphine were identified. Nausea or vomiting was rare (2%-6% of cases). The median (interquartile range) length of stay was 2.4 (1.6-3.75) hours.

**Conclusions and Relevance:**

These findings suggest that high-dose buprenorphine induction, adopted by multiple clinicians in a single-site urban ED, was safe and well tolerated in patients with untreated OUD. Further prospective investigations conducted in multiple sites would enhance these findings.

## Key Points

**Question:**

Is high-dose (>12 mg) buprenorphine induction safe and well tolerated in patients with untreated opioid use disorder who present to the emergency department?

**Findings:**

In this case series of 579 cases, 54 clinicians followed a high-dose buprenorphine (monoproduct) protocol. There were no documented episodes of respiratory depression or excessive sedation, and precipitated withdrawal was rare (0.8% of cases) and was not associated with dosing.

**Meaning:**

These findings suggest that high-dose buprenorphine induction, adopted by multiple clinicians in a single-site, urban emergency department, was safe and well tolerated in patients with untreated opioid use disorder.

## Teaser

This case series examines the safety and tolerability of high-dose (>12 mg) buprenorphine induction in the emergency department (ED) for treatment of opioid use disorder.

## Introduction

Initiation of buprenorphine for the treatment of opioid use disorder (OUD) in the emergency department (ED), combined with linkage to outpatient care, is an effective strategy to reduce mortality and morbidity among persons living with opioid addiction.^1,2^ With a total of 50 042 lives lost in 2019 because of opioid overdose deaths, projections of a 30% increase in 2020, and evidence that ED-initiated buprenorphine is both life-saving and cost-effective, the American College of Emergency Physicians, the US Surgeon General, the National Institute of Drug Abuse, and the Substance Abuse and Mental Health Services Administration have all called for ED access to initiation of buprenorphine for treatment of OUD.^3,4,5,6^ Yet, patients frequently face substantial barriers to obtaining buprenorphine in a timely manner after ED discharge because of a lack of ED clinicians with US Drug Enforcement Agency X-waivers permitting them to prescribe buprenorphine or other logistical issues in filling prescriptions associated with prior authorization requirements, transportation, and health insurance coverage gaps.^7,8,9,10,11^

The timing and dose of buprenorphine during induction may be an opportunity to address the lack of timely follow-up care after ED discharge. Existing treatment guidelines published by the Department of Health and Human Services,^12,13,14^ which were developed for office-based practice, limit the maximum sublingual (SL) buprenorphine induction dose during the first 24 hours to 8 to 12 mg. For most individuals with OUD, higher doses are required for effective agonist blockade and suppression of opioid withdrawal and craving.^15,16,17,18,19,20,21^ An accelerated induction procedure that achieves therapeutic buprenorphine levels obtained in less than 3 to 4 hours, vs the typical 2 to 3 days, could potentially increase safety during the crucial gap between ED discharge and continuation of treatment in the outpatient setting.^4,17,22^

**Figure 1.**
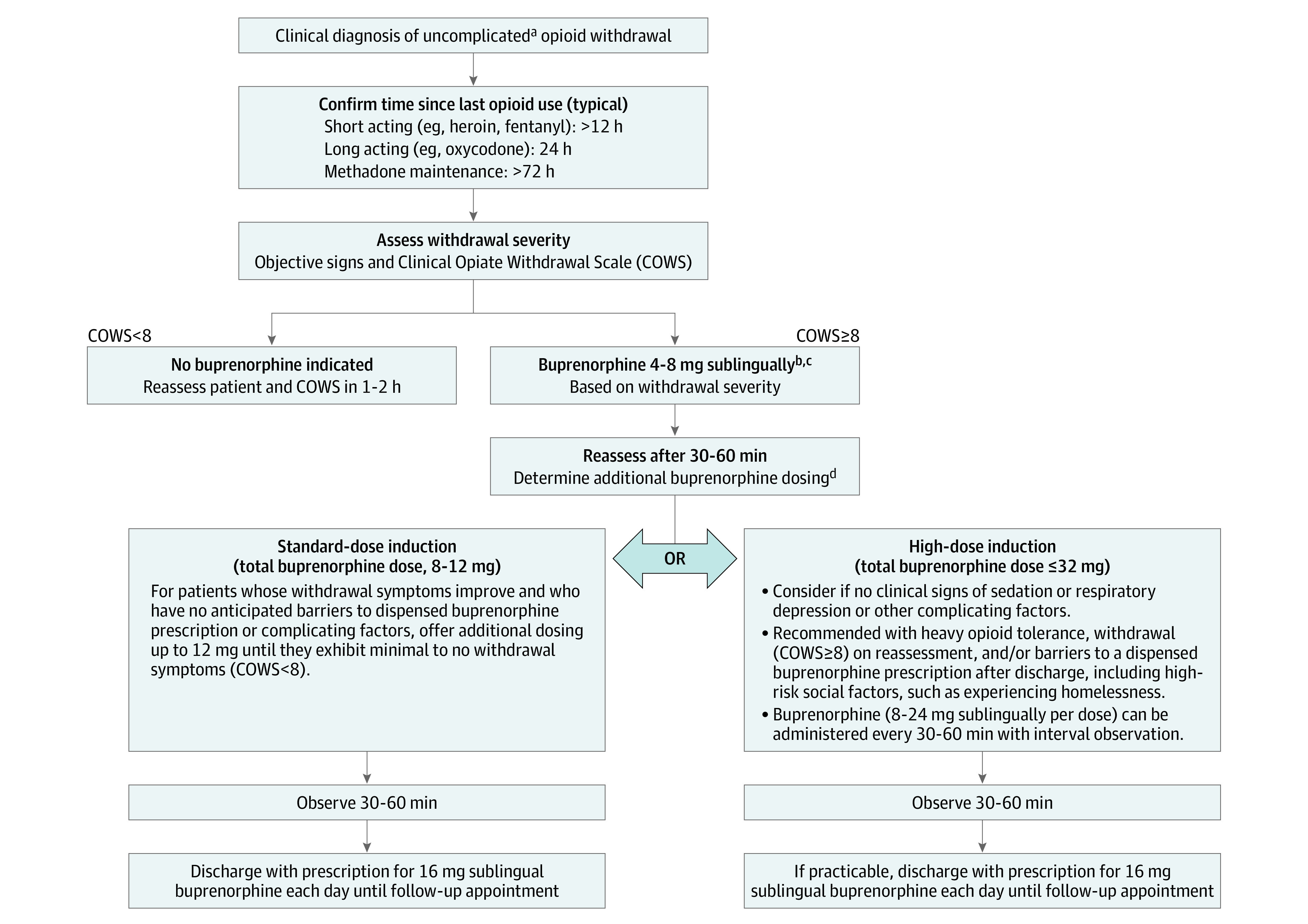
High-Dose Buprenorphine Treatment Pathway ^a^Complicating factors include age 65 years or older; altered mental status; viable pregnancy; methadone use; intoxication with alcohol, benzodiazepines, or other sedatives; postoverdose reversal with naloxone; anticipated surgery; long-term opioid therapy for pain; or any serious acute medical illness, such as heart failure, liver failure, kidney failure, or respiratory distress. ^b^Withdrawal is a clinical determination based on the Clinical Opiate Withdrawal Scale (COWS): mild, 5-12; moderate, 13-24; severe, greater than 25. A starting dose of 8 mg sublingual should be considered in moderate-to-severe withdrawal. ^c^Buprenorphine, sublingual monoproduct was used. ^d^Standard dose induction (8-12 mg) is associated with a lower risk of sedation, respiratory depression, and adverse effects, such as nausea and headache, particularly in patients with complicating factors; the duration and magnitude of withdrawal suppression is less. High doses may produce sedation, respiratory depression, nausea, and headache; the duration and magnitude of withdrawal suppression is greater.

Thus, we evaluated an ED high-dose (>12 mg) buprenorphine induction clinical pathway (Figure 1). We sought to describe patient and visit characteristics, clinical outcomes, and any adverse events associated with the high-dose protocol in a large cohort of patients with OUD presenting in opioid withdrawal.

## Methods

The Alameda Health System institutional review board approved this case series and granted a waiver of informed consent because the data were deidentified and the study posted minimal risk to participants. Study procedures followed the recommended reporting guideline for abstraction and reporting of retrospective case series data.^23,24^

### Study Design and Setting

We conducted a retrospective electronic health record (EHR) review of patients aged 18 years or older treated with SL-buprenorphine at a large, urban, safety net ED between January 1, 2018, and December 31, 2018. The purpose was to evaluate the safety and tolerability of an ED-implemented, high-dose clinical pathway for initiation of buprenorphine for treatment of OUD. Self-reported race and ethnicity were included in our analysis to describe the demographic profile of patients receiving buprenorphine treatment in the ED.

### High-Dose Buprenorphine Pathway

The high-dose ED buprenorphine induction pathway includes a dose option up to 32 mg SL to increase the magnitude and duration of opioid withdrawal suppression (Figure 1).^16,17,18,19,20,25^ Fifty-eight emergency medicine attending and resident physicians and 21 advanced practice practitioners (APPs) received training on the high-dose buprenorphine pathway initially during a live clinician workshop on September 19, 2017, which was video recorded and placed on the department’s website for future reference. Ongoing reinforcement at ED daily clinician rounds and case conferences were conducted iteratively throughout the next year. In-person technical assistance was provided Monday through Friday, 9:00 am to 5:00 pm, by 2 substance use navigators (SUNs). Each navigator had experience supporting more than 100 ED buprenorphine inductions. The pathway and supporting materials were posted in clinical areas, online,^26^ and in the EHR. There was no formal high-dose competency assessment, and an emergency physician who was board certified in addiction medicine was available at all times for on-call consultation.

The selection of patients for high-dose buprenorphine induction, defined as more than 12 mg of SL-buprenorphine during the ED stay, was based on the patient’s history, vital signs, physical examination findings, clinical judgment using scoring systems such as the Clinical Opioid Withdrawal Scale (COWS; mild, 5-12; moderate, 13-24; severe, >25), and evaluation of complicating factors.^17,27^ Specialty consultation was suggested for patients with complicating factors, including recent methadone use; anticipated surgery; receipt of long-term opioid therapy for pain; intoxication with alcohol, benzodiazepines, or other sedatives; postoverdose reversal with naloxone; chronic pulmonary disease; or serious acute illness and/or organ failure (Figure 1). All patients determined to be clinically appropriate for buprenorphine induction were recommended to receive an initial buprenorphine dose of 4 to 8 mg SL on the basis of their level of withdrawal and to be reassessed in 30 to 45 minutes. Patients with improvement in withdrawal symptoms after the initial dose and same-day access to a dispensed buprenorphine prescription after discharge were offered the standard-dose induction pathway. Doses of up to 12 mg could be administered to achieve minimal to mild withdrawal (COWS score <8). Patients without complicating medical factors but with barriers to immediate access to buprenorphine after discharge who also demonstrated no concerning clinical signs of sensitivity to buprenorphine, induced respiratory depression, or sedation were offered the high-dose induction pathway (Figure 1). The dosing of buprenorphine could be done in increments of 4 to 8 mg or with the full 24 mg at once, depending on the patient’s level of withdrawal. A collaborative process of shared decision-making with the patient was promoted during on-shift didactic sessions and one-on-one technical assistance provided by SUNs and an addiction specialist physician.^28,29^ Clinicians were not required to participate in the high-dose pathway. APPs were able to order buprenorphine without prior approval of a physician.

### Inclusion Criteria

All patients receiving treatment with SL-buprenorphine were included in the analysis. Ongoing quality assurance reconciliation of the buprenorphine tablet counts via the automated medication dispensing system and the EHR orders found agreement without discrepancies.

### Methods of Measurement

All relevant clinical documentation was abstracted from the EHR at Alameda Health System by a trained primary reviewer (A.A.V.) blinded to the study aims and a secondary reviewer (A.A.H.) blinded to abstraction of the primary reviewer. To minimize bias, a standardized data collection form and manual of operations including variable definitions was used.^30^ The data collection elements and variable definitions are in the eTable in the Supplement. Interrater reliability agreement for key study variables was assessed between reviewers in a pilot random sample of 10 records outside the 2018 study population. Key study variables included SL-buprenorphine dosing, respiratory rate, symptoms associated with opioid withdrawal or excessive buprenorphine toxicity, and adverse events. All measures were collected for all encounters included in the study. After acceptable interrater reliability was established in these 10 pilot records, 1 of every 10 study records was assessed for interrater agreement until the conclusion of data abstraction. Nurse, emergency clinician, and SUN documentation; patient chief complaint; vital signs including oxygen saturation; and use of supplemental oxygen and ancillary medications for withdrawal were recorded before and after administration of buprenorphine. In addition, the triage designation, Emergency Severity Index (range, 1-5, with higher scores indicating lower patient acuity), clinician type administering the buprenorphine, ED length of stay, and hospitalization were recorded.

A clinical form completed by the SUN prospectively captured additional data on the patient’s opioid use history, buprenorphine dosing details, and experience in the ED, including treatment outcomes, such as occurrence of worsening or precipitated withdrawal, excessive sedation, nausea and/or vomiting, or any other adverse event. Potential adverse events were all reviewed by expert clinicians independent of the clinical site (K.F.H. and G.D.). All return medical visits within 24 hours of discharge from the buprenorphine initiation visit were reviewed for documentation of an adverse event after discharge from the baseline ED visit. Data abstraction for each encounter were entered into Research Electronic Data Capture software version 7.3.6 (Vanderbilt University).^30^ Raters had 1.00 agreement for all extracted variables in both pilot and full study samples using the weighted Cohen κ test.

### Outcome Measures

Our primary outcomes were (1) the occurrence of precipitated withdrawal and (2) any other serious adverse event attributable to buprenorphine administration, including sedation, decreased respiratory rate, hypoxia, and/or naloxone rescue administration in ED or in the 24 hours after discharge. We used the Opioid–Common Terminology Criteria for Adverse Events definitions: grade 3 (hospitalizations), grade 4 (life-threatening event; urgent intervention required), or grade 5 (death).^31^ Grades 4 and 5 were considered serious adverse events. Secondary outcomes abstracted were signs and symptoms recorded from the EHR that were derived from standardized and validated instruments to assess withdrawal (COWS)^27^ and excessive buprenorphine toxicity (Opioid-32 questionnaire).^32^ Because of the retrospective study design, the actual scales themselves were not documented and, thus, could not be calculated as intended. Precipitated withdrawal was defined as ED clinician diagnosis of precipitated withdrawal or documentation of increasing severity of COWS score within 1 hour after buprenorphine administration.^33,34^ Any new nausea and/or vomiting documented in the hour after buprenorphine administration was recorded. Other opioid-related adverse effects (eg, headache, itchiness, or urinary retention) were categorized using items from the Opioid Related Symptom Distress Scale.^35^

### Statistical Analysis

Characteristics were summarized using frequencies (percentages) for categorical variables and means (SD) or medians (interquartile ranges) for continuous variables. Comparisons across buprenorphine doses were made using the Kruskal-Wallis test for continuous variables and χ^2^ or Fisher exact tests for categorical variables. The χ^2^ test for trend was used to compare Emergency Severity Index between total dose groups. All statistical tests were 2-sided. Significant associations (*P* < .05) were followed by post hoc pairwise comparisons of all doses. Given the exploratory nature of this study and the goal of illuminating potential safety signals, no adjustment to type I error was performed. Summaries were made at an encounter level treating encounters as independent events. Statistical analysis was performed with R statistical software version 4.0.2 (R Project for Statistical Computing). Data analysis was performed from April 2020 to March 2021.

## Results

### Participants

A total of 391 unique ED patients (median [interquartile range] age, 36 [29-48] years; 267 men [68.3%]) were identified as having been treated with SL-buprenorphine (monoproduct) between January 1, 2018, and December 31, 2018, during a total of 579 ED visits. Of the patients, 170 (43.5%) were Black, and 57 (14.6%) were Hispanic or Latino. Many patients were homeless (88 patients [22.5%]) and had comorbid non–substance use–related psychiatric disorders (161 patients [41.2%]). More than one-half (209 patients [53.5%]) had never been treated with (or self-prescribed illicitly) buprenorphine (Table 1).

**Table 1.** Baseline Demographic and Clinical Characteristics of Patients Receiving Sublingual Buprenorphine Induction for Opioid Use Disorder

Characteristic	Patients, No. (%) (n = 391)
Sex	
Male	267 (68.3)
Female	124 (31.7)
Age, y	
18-25	38 (9.7)
26-34	138 (35.3)
35-44	93 (23.8)
45-54	60 (15.3)
55-64	48 (12.3)
65-73	14 (3.6)
Race^a^	
Black	170 (43.5)
White	148 (37.8)
Other race^b^	73 (18.7)
Ethnicity^a^	
Hispanic or Latino	57 (14.6)
Non-Hispanic or non-Latino	334 (85.4)
Insurance status	
Medi-Cal	274 (70.1)
Medicare	26 (6.7)
Military	1 (0.3)
Other public insurance	12 (3.1)
Private	23 (5.9)
No insurance	49 (12.5)
Homeless^c^	
Yes	88 (22.5)
No	303 (77.5)
Psychiatric diagnosis^d^	
Yes	161 (41.2)
No	230 (58.8)
Buprenorphine exposure history^e^	
No	209 (53.5)
Yes	176 (45.0)
Emergency department visits, No.^f^	
1	292 (74.7)
2-4	86 (22.0)
5-14	13 (3.3)

^a^ Reported by patients.

^b^ Refers to Asian, Pacific Islander, Native American, or unknown.

^c^ Documented by the emergency department staff.

^d^ Refers to any non–substance use psychiatric diagnosis in the health care record.

^e^ Reported by patients and documented in the electronic health record.

^f^ Refers to visits during the study period January 1, 2018, to December 31, 2018.

### Outcomes

**Figure 2.**
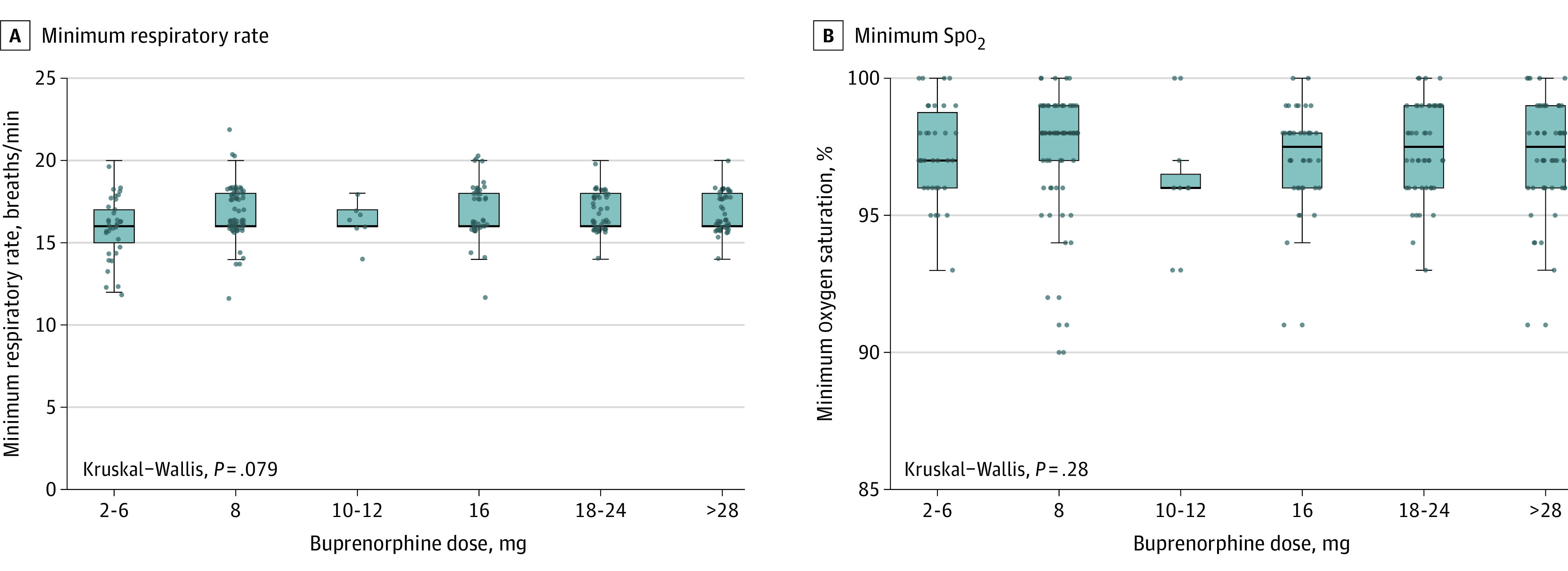
Minimum Respiratory Rate and Oxygen Saturation (SpO_2_) Following Initial Dose by Buprenorphine Dose Boxes correspond to 25th and 75th percentiles, with lines in boxes denoting medians. Dots denote outliers. Error bars denote 95% CIs. Kruskal-Wallis test compares distributions of respiratory rate and oxygen saturation across buprenorphine dose categories.

All 579 ED visits were grouped into escalating categories of SL- buprenorphine dose and analyzed. There were 366 (63.2%) high-dose inductions using more than 12 mg of SL-buprenorphine, including 138 doses (23.8%) greater than or equal to 28 mg (Table 2). Review of vital sign documentation, including measurements of blood pressure, respiratory rate, heart rate, and oxygen saturation, found no significant association with total buprenorphine dose. No patient was administered naloxone at any time after buprenorphine administration. We found no documentation of decreased respiratory rate in the highest dose group of 28 mg or higher (Figure 2). Supplemental oxygen was administered during 17 encounters. Three patients with documented chronic obstructive pulmonary disease accounted for 9 of those encounters, and all oxygen supplements were administered before buprenorphine. The overall median (interquartile range) length of stay in the ED was 2.4 (1.6-3.75) hours. Most patients were triaged as low severity and were treated by an APP. The documented incidence of nausea or vomiting after buprenorphine was low (2%-6% of cases) with rates of 4% for a dose of 8 mg, 6% for doses of 10 to 12 mg, 5% for doses of 16 mg, 2% for doses of 20 to 24 mg, and 2% for doses of 28 mg or higher.

**Table 2.** Clinical Characteristics of Sublingual Buprenorphine Induction for Opioid Use Disorder During Emergency Department Visits

Characteristic	Total buprenorphine dose sublingual						*P* value^a^
	2-6 mg (n = 55)	8 mg (n = 136)	10-12 mg (n = 22)	16 mg (n = 106)	20-24 mg (n = 122)	≥28 mg (n = 138)	
Systolic blood pressure, median (IQR), mm Hg							
At triage	133 (120-150)	132 (120-150)	132 (110-140)	128 (120-140)	128 (120-150)	130 (120-140)	.75
Maximum	135 (130-160)	140 (130-160)	140 (130-150)	133 (120-150)	134 (120-150)	142 (120-160)	.48
Minimum	118 (110-130)	117 (110-130)	103 (97-130)	116 (100-130)	116 (110-130)	121 (110-140)	.83
Respiratory rate, median (IQR), breaths/min							
At triage	18 (16-18)	18 (16-18)	18 (16-18)	18 (16-18)	18 (17-18)	18 (17-18)	.26
Maximum	18 (18-18)	18 (18-20)	18 (17-18)	18 (18-20)	18 (18-20)	18 (18-20)	.23
Minimum	16 (15-17)	16 (16-18)	16 (16-17)	16 (16-18)	16 (16-18)	16 (16-18)	.08
Heart rate, median (IQR), beats/min							
At triage	84 (70-98)	89.5 (78-100)	87 (81-94)	83.5 (75-95)	88 (80-100)	87 (79-99)	.16
Maximum	87 (77-100)	92.5 (82-100)	88 (76-98)	90 (80-100)	95.5 (87-100)	96.5 (81-100)	.73
Minimum	71 (63-80)	76 (68-85)	64 (60-84)	77 (66-86)	80 (70-89)	76 (71-88)	.26
Temperature, °F	98 (97-98)	97.8 (97-98)	97.6 (97-98)	97.5 (97-98)	97.9 (97-98)	97.8 (97-98)	.24
Oxygen saturation, median (IQR), %							
At triage	99 (98-100)	99 (98-100)	98 (97-100)	99 (98-100)	99 (98-100)	99 (98-100)	.29
Maximum	100 (99-100)	99 (99-100)	100 (98-100)	99 (98-100)	99 (98-100)	99 (99-100)	.13
Minimum	97 (96-99)	98 (97-99)	96 (96-96)	97.5 (96-98)	97.5 (96-99)	97.5 (96-99)	.25
Supplemental oxygen, patients, No. (%)	6 (11)	3 (2.2)^b^	1 (4.5)	4 (3.8)	2 (1.6)^b^	1 (0.72)^b^	.01
Chronic obstructive pulmonary disease diagnosis, patients, No. (%)	17 (2.9)	2 (3.6)	3 (2.2)	1 (4.5)	4 (3.8)	2 (1.6)	.76
Emergency Severity Index, patients, No. (%)							
1	0 (0)	0 (0)	0 (0)	0 (0)	0 (0)	0 (0)	.10
2	7 (13)	9 (6.6)	0 (0)	5 (4.7)	4 (3.3)	5 (3.6)	
3	24 (44)	38 (28)	5 (23)	31 (29)	38 (31)	33 (24)	
4	15 (27)	63 (46)	11 (50)	46 (43)	64 (52)	81 (59)	
5	9 (16)	26 (19)	6 (27)	24 (23)	16 (13)	19 (14)	
Length of stay, median (IQR), h	3.5 (2.4-5.8)	2.6 (1.7-4.4)^b^	2.6 (2.1-3.7)	2.1 (1.5-3.5)^b^^,^^c^	2.2 (1.4-3.3)^b^	2.3 (1.7-3.6)^b^	.002
Clinician type, No. (%)							
Advance practice provider	22 (40)	72 (53)	15 (68)^b^	64 (60)^b^	87 (71)^b^^,^^c^	99 (72)^b^^,^^c^	<.001^d^
Medical doctor	33 (60)	64 (47)	7 (32)	42 (40)	35 (29)	39 (28)	
Adverse events, No. (%)							
Precipitated withdrawal	0	4 (2.9)	0	0	0	1 (0.7)	.20
Hospitalization	5 (9.1)	4 (2.9)	1 (4.5)	3 (2.8)	8 (6.6)	4 (2.9)	.26
Return to ED within 24 h	2 (3.6)	10 (7.4)	3 (14.0)	9 (8.5)	6 (4.9)	15 (11.0)	.32
Time to return to ED within 24 h, median (IQR), h	13.8 (12-16)	11.4 (5.9-14)	17.8 (11-20)	.4 (6.5-23)	15.1 (13-18)	18.4 (14-22)	.52

Abbreviations: ED, emergency department; IQR, interquartile range.

SI conversion factor: To convert degrees Fahrenheit to degrees Celsius, subtract 32 and multiply by 0.556.

^a^ *P* values are for any differences among categories of total buprenorphine sublingual dose. After significant omnibus test, all pairwise comparisons were performed. Results of pairwise dose category comparisons that are significant are marked by a footnote indicating which column was different.

^b^ *P* < .05 for pairwise comparison to 2- to 6-mg total dose.

^c^ *P* < .05 for pairwise comparison to 8-mg total dose.

^d^ *P* value for any difference in the proportions of encounters that were by advance practice practitioners across the total buprenorphine dose categories.

**Figure 3.**
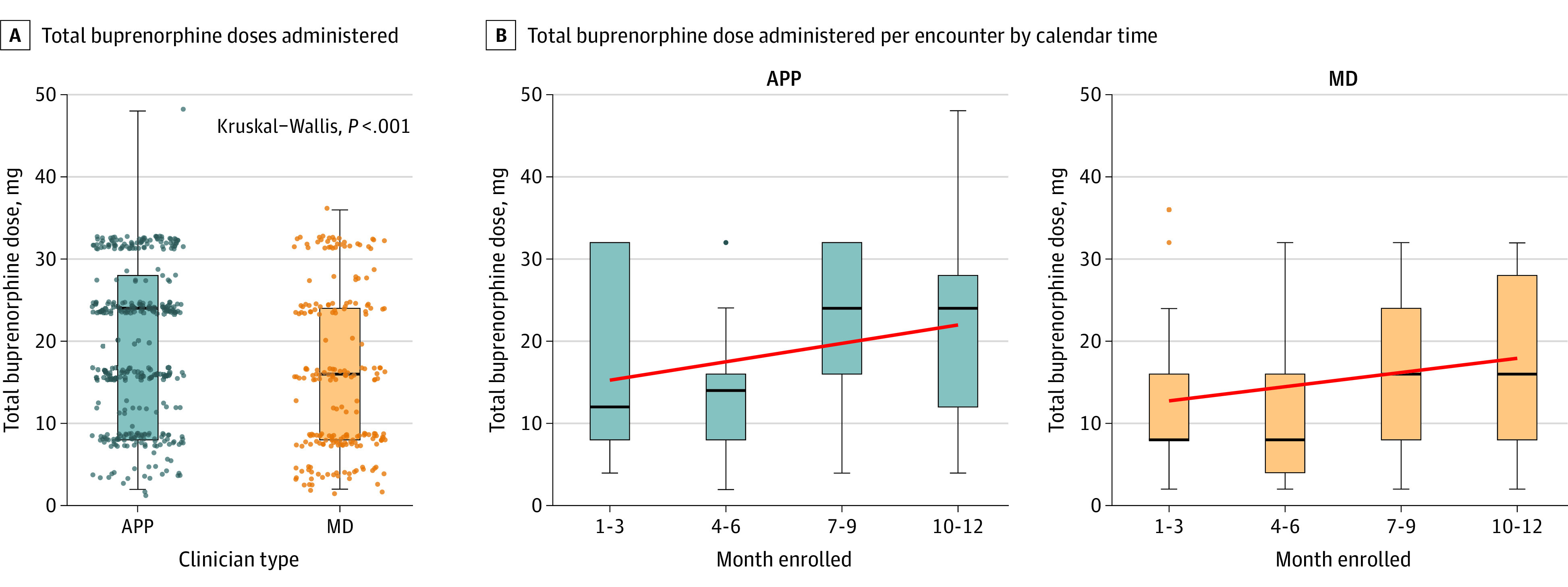
Buprenorphine Doses Administered by Physicians (MDs) and Advanced Practice Practitioners (APPs) A, Total doses are shown. Each dot represents a unique patient encounter. Boxes correspond to 25th and 75th percentiles, with lines in boxes denoting medians. Error bars denote 95% CIs. Kruskal-Wallis test compares distributions of respiratory rate and oxygen saturation across buprenorphine dose categories. MDs provided high-dose buprenorphine in 71 of 220 patient encounters (32%), and APPs provided high-dose buprenorphine in 181 of 359 encounters (50%). B, Total buprenorphine dose administered per encounter by calendar time (in quarter years) of encounter. Red line indicates the best fit line. Error bars denote 95% CIs. The Spearman correlation of dose administered by time was 0.23 (*P* < .001) overall, 0.19 (*P* = .005) for MDs, and 0.22 (*P* < .001) for APPs. Dots denote outliers. Two patients received higher dosing. One patient in the MD group received 36 mg as the inpatient team ordered an additional buprenorphine 20 hours into the admission while the patient boarded in the ED. They did well and followed-up in the hospital bridge clinic. The second patient in the APP group who received 48 mg was experiencing homelessness, used excessively high amounts of opioids daily, and insisted they needed large buprenorphine doses for symptom control. They were seen previously in the ED, with demonstrated tolerance to high dosing. They went to an inpatient substance treatment program.

Overall, 54 unique clinicians were observed in our study. Among them, all 21 APPs (100%) and 29 of 33 attending physicians (88%) administered high-dose buprenorphine at least once, ranging from 2 to 39 cases for APPs and 1 to 28 cases for physicians. Over time, clinicians tended toward use of an increasing total dose of buprenorphine (Figure 3).

### Precipitated Withdrawal

There were 5 cases (event rate, 0.8%) of documented precipitated withdrawal among 579 visits with buprenorphine administration (Table 2). Of these, 4 cases occurred after usual dosing of 8 mg and, thus, were unrelated to high-dose buprenorphine. Additional buprenorphine—for a total dose of 28 mg—successfully treated the precipitated withdrawal, and in all cases the patients were discharged in stable or improved conditions. In the fifth case, the patient tolerated a dose of 8 mg and experienced precipitated withdrawal after administration of an additional 24 mg of buprenorphine. After further EHR review, the patient had concurrent stimulant use that may have contributed to the clinical picture.

### Serious Adverse Events

There were 3 life-threatening adverse events requiring urgent intervention (grade 4 determined by the Common Terminology Criteria for Adverse Events definition). One patient was found to have diabetic ketoacidosis. The second patient presented to the ED on 2 separate occasions in withdrawal and was hospitalized for acute myocardial infarction. All 3 events were determined to be unrelated to their buprenorphine induction. There were no serious adverse events associated with buprenorphine administration, including sedation, hypoxia, and/or naloxone rescue in the ED or 24 hours after discharge. No patients were admitted for treatment of buprenorphine precipitated withdrawal. There were 25 hospitalizations attributable to the primary medical complaints with buprenorphine used as an adjunctive treatment for co-occurring opioid withdrawal. Fifteen of the 25 patients (60%) were admitted for treatment of an infection—abscess, cellulitis, septic arthritis, or pneumonia. Five patients (20%) were admitted for chronic obstructive pulmonary disease. The remaining diagnoses included malignant entity, heart failure, Crohn disease, and stimulant intoxication. Hospitalization was not associated with buprenorphine dose (Table 2).

Forty-five participants had return visits within 24 hours of discharge, with return rates ranging from 3.6% to 14.0% across dose categories (Table 2). In review of return visits, there were no cases of precipitated withdrawal, opioid overdose, sedation, respiratory depression, or other adverse event attributable to buprenorphine treatment. Some patients (10%-18%) were unsuccessful accessing follow-up treatment immediately after discharge and required repeat dosing in the ED.

## Discussion

In this large case series of ED patients with OUD treated with a high-dose pathway of buprenorphine induction, we observed no instances of buprenorphine toxicity (eg, sedation or respiratory depression), and adverse events including precipitated withdrawal were rare. Our low rate of precipitated withdrawal is consistent with other ED studies using a similar protocol but without the high-dose pathway. To date and to our knowledge, there have been no formal studies of high-dose strategies, although they are used sporadically. Our findings suggest that the high-dose induction pathway is safe, addresses withdrawal symptoms rapidly, removes barriers to short-term medication access, and extends the duration of action of buprenorphine, all of which offer substantial benefit to the individual and potentially improve follow-up. This benefit has heightened clinical importance most recently because of the limitations for prompt follow-up caused by the COVID-19 pandemic.

The current SL-buprenorphine induction guidance provided by the Substance Abuse and Mental Health Services Administration^12,13^ assumes that a patient has access to a dispensed buprenorphine prescription and recommends starting at 2 to 4 mg, followed by a second dose of 2 to 4 mg after approximately 2 hours, with a maximum of 8 to 12 mg on day 1 and 16 mg on day 2. This multiday induction prolongs the time for maximum saturation of μ-opioid receptors and reduction in cravings.^20^ In the 2017 Food and Drug Administration labeling update to buprenorphine,^14^ inadequate dose escalation on initiation has been identified as a reason that patients stop receiving treatment. Patients often face substantial delays in accessing the dispensed medication after discharge.^36,37^ Thus, multiday induction recommendations may inadvertently prolong symptoms in patients presenting to EDs for opioid withdrawal.^16,17,38^

The high-dose pathway allows for a consideration of patients’ social determinants of health, a major issue for most ED patients with OUD.^39^ Almost one-quarter of our patients were homeless, 41.2% had co-occurring non–substance use–related psychiatric disorders, and the majority were uninsured or underinsured. Some patients (10%-18%) were unsuccessful accessing follow-up treatment immediately after discharge and required repeat dosing in the ED. Higher dosing provides the individual with a critical extended period without craving, allowing more opportunity to navigate the barriers to follow-up, where obstacles to treatment engagement are more easily addressed.^38^ Repeat ED engagement after buprenorphine initiation may also provide harm reduction as patients navigate the complex and, at times, prolonged transition from buprenorphine initiation to retention in maintenance outpatient addiction treatment.^38,40^

The high-dose pathway was widely accepted and adopted by a large proportion of ED clinicians, with 88% of physicians and 100% of APPs treating patients with buprenorphine. The ED length of stay was remarkably short, with a median of just over 2 hours for most patients with high-dose administration and often occurred in low-acuity areas, thus dispelling the myth perpetuated by many ED clinicians that adoption of ED-initiated buprenorphine is complex and time-consuming. The advantage over slower, more rigid, buprenorphine inductions is that patients can be more collaborative, rather than being forced into a treatment pathway that may not meet their needs.^29^

Not all patients should receive a high-dose induction. Our findings suggest that clinicians followed guidance to exercise caution with larger doses in patients who are more ill. For example, we found that a larger percentage of patients requiring hospitalization were treated with lower doses of buprenorphine most likely consistent with the protocol. Although the safety profile of buprenorphine and the ceiling effect on its depression are well established, in ill or intoxicated patients with reduced respiratory reserve, the sedation and respiratory depression of buprenorphine can be clinically important.^41,42^

In the context of the opioid overdose crisis and COVID-19 pandemic, consideration of the perceived risks of precipitated withdrawal and oversedation in rapid dose escalation should be balanced by considerations of the risk of delay in treatment. In a study^43^ of primary care patients with unobserved home induction of buprenorphine, rates of precipitated withdrawal were 3%, greater than what we observed. The extended opioid blockade provided by the high-dose pathway may impart more substantial overdose protection and more effective blunting of the euphoric and reinforcing effects of any opioids used in the high-risk window following ED departure prior to engagement in follow-up care.^20^ The increasing prevalence of high-potency illicit opioids, such as fentanyl and fentanyl analogues, likely increases the risk of overdose after ED discharge, making the protective properties of buprenorphine all the more important.^44,45^

### Limitations

The primary limitations of this study are its retrospective design in a single site. We relied on clinical documentation as the source of data for evaluation of our primary and secondary outcomes and did not prospectively compare the high-dose clinical pathway with the traditional induction strategies. Clinicians did not always document a complete COWS score but did document pertinent symptoms, such as large pupils or diaphoresis, or a summative clinical impression of opioid withdrawal. Thus, comparison of initial and subsequent COWS scores was not available for analysis. As an early California Bridge site, ED clinicians received extensive training in assessing and treating patients with OUD.^26^

Risks inherent to retrospective reviews, such as incomplete and/or inconsistent documentation found in the EHR and the reliability of abstracting it accurately, were mitigated by the use of a predetermined robust collection instrument by a well-trained blinded research assistant and excellent interrater reliability determined using a separate cohort of patients before study extraction. Potential adverse events were reviewed by expert clinicians who were independent of the clinical site. The expertise of the clinicians provides assurances related to the quality of the relevant data abstracted from the medical record (ie, signs and symptoms of opioid withdrawal and opioid-related distress)^27,31,35^ and makes it unlikely that a serious adverse event, such as precipitated withdrawal or excessive sedation, would not be documented. Precipitated withdrawal is not subtle, and its signs and symptoms are readily apparent and would result in medications administered. Documentation by the physician, APP, or nurse would be available.

The results may not be generalizable to other EDs without staff expertise and with the availability of other buprenorphine formulations, because only the monoproduct was used. It is possible that high-dose induction using a combination buprenorphine-naloxone formulation could result in clinically meaningful absorption of naloxone.^46^

## Conclusions

At a single, urban ED, a high-dose buprenorphine treatment pathway was a safe and effective method of induction and did not result in increased incidence of precipitated withdrawal, oversedation, or other adverse events attributable to buprenorphine. A therapeutic dose of buprenorphine was achieved within less than 3 hours of ED stay, mostly in low-acuity treatment area.

## Notes

Supplement. **eTable.** Data Dictionary
