# Supplementary material for: High-Dose Buprenorphine Induction in the Emergency Department for Treatment of Opioid Use Disorder
Source: JAMA Netw Open. 2021 Jul 15;4(7):e2117128. doi: 10.1001/jamanetworkopen.2021.17128 (PMC8283555; doi:10.1001/jamanetworkopen.2021.17128)
Supplement: Supplement. — eTable. Data Dictionary [file jamanetwopen-e2117128-s001.pdf]

Supplemental Online Content

Herring AA, Vosooghi AA, Luftig J, et al. High-dose buprenorphine induction in the emergency department for treatment of opioid use disorder. *JAMA Netw Open*. 2021;4(7):e2117128.
doi:10.1001/jamanetworkopen.2021.17128

eTable. Data Dictionary

This supplemental material has been provided by the authors to give readers additional information about their work.

© 2021 Herring AA et al. *JAMA Network Open.*

eTable. Data Dictionary

Data Dictionary Codebook

#	Variable / Field Name	Field Label<br>*Field Note*	Field Attributes (Field Type, Validation, Choices, Calculations, etc.)
1	**record_id**	**Record ID**	**text**
2	**reviewer**	**Chart Reviewer Name**<br>*Enter as: Last Name, First Initial.*	**radio, Required**<br>**1** Herring, A.<br>**2** Vosooghi, A.<br>**3** Vuong, C.<br>**4** Other
3	**reviewer_oth**<br>**Show the field ONLY if: [reviewer] = '4'**	**If "Other," please specify as "Last name, First initial."**	**text, Required**
4	**name**	**Section Header:** *Demographics*<br>**Patient's full name as it appears in Wellsoft: Last name, First name**<br>*Copy and paste from Wellsoft (click on the relevant ield in Chart, then Ctrl-C).*	**text, Required, Identifier**
5	**dob**	**Date of birth**<br>*Copy and paste from Wellsoft.*	**text (date_mdy), Required, Identifier**
6	**age**	**Age**<br>*Calculated from dob.*	**calc, Required, Identifier**<br>**Calculation: rounddown(datediff("today", [dob], "y", "mdy"), 0)**
7	**gender**	**Gender**<br>*Trans and GNC information can be found in HPI.*	**radio, Required**
8	**arrival**	**Date and time of arrival**<br>*Copy and paste from Wellsoft*	**text (datetime_mdy), Required, Identifier**
9	**mrn**	**MRN**<br>*Copy and paste from Wellsoft.*	**text (integer, Min: 0, Max: 999999999), Required, Identifier**
10	**encounter**	**Encounter #**<br>*Copy and paste from Wellsoft.*	**text, Required, Identifier**

© 2021 Herring AA et al. *JAMA Network Open.*

11	**race**	**Race**<br>*Look in "View: Patient Charts - All Fields" in Wellsoft.*	**radio, Required**<br>1 American Indian or Alaska Native<br>2 Asian Indian<br>3 Black<br>4 Chinese<br>5 Filipino<br>6 Guamanian or Chamorro<br>7 Japanese<br>8 Korean<br>9 Native Hawaiian<br>10 Other Asian<br>11 Other Pacific Islander<br>12 Other Race<br>13 Samoan<br>14 Vietnamese<br>15 White<br>16 Other Race<br>999 Unknown
12	**ethnicity**	**Ethnicity**<br>*1 = Hispanic or Latino; 2 = Non-Hispanic or Non-Latino; 4 = Other Hispanic or Latino*	**radio, Required**<br>1 Hispanic or Latino<br>2 Non-Hispanic or Non-Latino<br>999 Unknown/Declined to state
13	**address**	**Mailing Address**<br>*Copy and paste from Wellsoft. Write "N/A" if none listed (i.e., homeless).*	**text, Required, Identifier**
14	**city**	**City**<br>*Copy and paste from Wellsoft. Write "N/A" if none listed.*	**text, Required, Identifier**
15	**state**	**State**<br>*Copy and paste from Wellsoft. Write "N/A" if none listed.*	**text, Required, Identifier**
16	**zip**	**Zip code**<br>*Enter 99999 if no zip code available.*	**text (zipcode), Required, Identifier**
17	**homeless**	**Homeless**<br>*Back-ill from chart, if necessary.*	**radio, Required**<br>1 True<br>0 False<br>999 Blank
18	**emerg_phone**	**Section Header:** *Ext Dmg*<br>**Emergency Contact # ******<br>*Found under "Ext Dmg." Enter 999 999 9999 if none listed.*	**text, Required, Identifier**

© 2021 Herring AA et al. *JAMA Network Open.*

	19	**insurance_1**	**Section Header:** *Financial*<br>**Insurance status ******<br>*Look in "View: Patient Charts - All Fields" in Wellsoft. If none listed, check against ED Bridge intake form.*	**checkbox, Required**<br>1 insurance_1_1 Private<br>2 insurance_1_2 Medi-Cal<br>3 insurance_1_3 Medicare<br>4 insurance_1_4 Military (i.e. Tricare, VA, etc)<br>5 insurance_1_5 Indian Health Service<br>6 insurance_1_6 County based (HealthPAC, Healthy SF, etc.)<br>7 insurance_1_7 Other public insurance<br>8 insurance_1_8 No insurance<br>999 insurance_1_999 Unknown
	20	**cc**	**Section Header:** *RN/Triage*<br>**Chief complaint**<br>*Copy and paste from Wellsoft chart CC. Also use this space to specify "Other" from the ED Bridge Initial Encounter sheet if applicable.*	**notes, Required**
	21	**mode_arrival**	**Mode of arrival**	**radio, Required**<br>1 Ambulatory/ Private Vehicle/ Wheelchair<br>2 ALS<br>3 BLS<br>4 Law Enforcement<br>5 Other<br>999 None listed
	22	**mode_arrival_oth**<br>**Show the field ONLY if: [mode_arrival] = '5'**	**If "Other," please specify.**	**text, Required**
	23	**ht**	**Pt's height (cm)**<br>*Enter 999 if not done this visit.*	**text, Required**
	24	**weight**	**Pt's weight (kg)**<br>*Enter 999 if not done this visit.*	**text, Required**
	25	**attending**	**Section Header:** *MD Chart*<br>**MD ED**<br>*Last name and irst initial, as it appears in the MD Chart section. Enter "N/A" if none listed.*	**text, Required**
	26	**res**	**Res/PA/NP**<br>*Last name and irst initial, as it appears in the MD Chart section. Enter "N/A" if none listed.*	**text, Required**
	27	**bup_rx**	**Section Header:** *Script / Rx*<br>**Was a prescription for buprenorphine given during the patient's ED visit? ******	**radio, Required**<br>1 Yes<br>0 No

© 2021 Herring AA et al. *JAMA Network Open.*

	28	**bup_rx_form**<br><br>**Show the field ONLY if:**<br>**[bup_rx] = '1'**	**Formulation**<br>*Look under Rx 1, Rx 2, etc.*	**radio, Required**<br>1 Buprenorphine/Naloxone<br>2 Buprenorphine
	29	**bup_rx_route**<br><br>**Show the field ONLY if:**<br>**[bup_rx] = '1'**	**Route**<br>*From "Freq/Rte"*	**radio, Required**<br>1 SL<br>2 BUCC<br>3 TD<br>4 IJ<br>5 IV<br>6 IM<br>7 ID<br>8 SQ<br>9 Other<br>999 Unknown
	30	**bup_rx_route_oth**<br><br>**Show the field ONLY if:**<br>**[bup_rx_route] = '9'**	**If "Other," please specify.**	**text, Required**
	31	**bup_rx_combo_strength**<br><br>**Show the field ONLY if:**<br>**[bup_rx_form] = '1'**	**Strength (in mg buprenorphine/mg naloxone)**<br>*From "Dose/Conc"*	**radio, Required**<br>1 2/0.5<br>2 4/1<br>3 8/2<br>4 12/3
	32	**bup_rx_mono_strength**<br><br>**Show the field ONLY if:**<br>**[bup_rx_form] = '2'**	**Strength (in mg)**	**radio, Required**<br>1 2<br>2 8
	33	**bup_rx_freq**<br><br>**Show the field ONLY if:**<br>**[bup_rx] = '1'**	**Frequency**<br>*From "Freq/Rte"*	**radio, Required**<br>1 QD<br>2 Every other day<br>3 BID<br>4 TID<br>5 QID<br>6 QH<br>7 QHS<br>8 Q4h<br>9 Q4-6h<br>10 QWK<br>11 Other<br>999 Unknown
	34	**bup_rx_freq_oth**<br><br>**Show the field ONLY if:**	**[bup_rx_freq] = '11'**	

© 2021 Herring AA et al. *JAMA Network Open.*

If "Other," please specify.

text, Required

© 2021 Herring AA et al. *JAMA Network Open.*

	35	bup_rx_num<br><br>Show the field ONLY if:<br>[bup_rx] = '1'	**# of doses**<br>*From "Disp." Enter numerical value.*	text, Required
	36	mult_bup_rx<br><br>Show the field ONLY if:<br>[bup_rx] = '1'	**Was more than one prescription for buprenorphine given during the patient's ED visit?**	yesno, Required<br>1 Yes<br>0 No
	37	bup_rx_form_2<br><br>Show the field ONLY if:<br>[mult_bup_rx] = '1'	**Formulation**<br>*Look under Rx 1, Rx 2, etc.*	radio, Required<br>1 Buprenorphine/Naloxone<br>2 Buprenorphine
	38	bup_rx_route_2<br><br>Show the field ONLY if:<br>[mult_bup_rx] = '1'	**Route**<br>*From "Freq/Rte"*	radio, Required<br>1 SL<br>2 BUCC<br>3 TD<br>4 IJ<br>5 IV<br>6 IM<br>7 ID<br>8 SQ<br>9 Other<br>999 Unknown
	39	bup_rx_route_oth_2<br><br>Show the field ONLY if:<br>[bup_rx_route_2] = '9'	**If "Other," please specify.**	text, Required
	40	bup_rx_combo_strength_2<br><br>Show the field ONLY if:<br>[bup_rx_form_2] = '1'	**Strength (in mg buprenorphine/mg naloxone)**<br>*From "Dose/Conc"*	radio, Required<br>1 2/0.5<br>2 4/1<br>3 8/2<br>4 12/3
	41	bup_rx_mono_strength_2<br><br>Show the field ONLY if:<br>[bup_rx_form_2] = '2'	**Strength (in mg)**	radio, Required<br>1 2<br>2 8

© 2021 Herring AA et al. *JAMA Network Open.*

	42	**bup_rx_freq_2**<br><br>**Show the field ONLY if: [mult_bup_rx] = '1'**	**Frequency**<br>*From "Freq/Rte"*	**radio, Required**<br>1 QD<br>2 Every other day<br>3 BID<br>4 TID<br>5 QID<br>6 QHS<br>7 Q4h<br>8 Q4-6h<br>9 QWK<br>10 Other<br>999 Unknown
	43	**bup_rx_freq_oth_2**<br><br>**Show the field ONLY if: [bup_rx_freq_2] = '10'**	**If "Other," please specify.**	**text, Required**
	44	**bup_rx_num_2**<br><br>**Show the field ONLY if: [mult_bup_rx] = '1'**	**# of doses**<br>*From "Disp." Enter numerical value.*	**text, Required**
	45	**bup_prescriber**<br><br>**Show the field ONLY if: [bup_rx] = '1'**	**Name the prescriber.**<br>*Copy and paste from "MD ED Last" in Wellsoft*	**text, Required**
	46	**other_rx**	**Was a medication prescribed to treat a co-occurring medical or psychiatric disorder?**	**yesno, Required**<br>1 Yes<br>0 No
	47	**oth_rx_num**<br><br>**Show the field ONLY if: [other_rx] = '1'**	**How many other medications were prescribed?**<br>*Not including buprenorphine*	**radio, Required**<br>1 1<br>2 2<br>3 3<br>4 4<br>5 5<br>6 6<br>7 7
	48	**oth_rx_1**<br><br>**Show the field ONLY if: [oth_rx_num] = '1' or [oth_rx_num] = '2' or [oth_rx_num] = '3' or [oth_rx_num] = '4' or [oth_rx_num] = '5' or [oth_rx_num] = '6' or [oth_rx_num] = '7'**	**Please list the (first) medication prescribed.**	**text, Required**

© 2021 Herring AA et al. *JAMA Network Open.*

	49	**oth_rx_2**<br><br>**Show the field ONLY if: [oth_rx_num] = '2' or [oth_rx_num] = '3' or [oth_rx_num] = '4' or [oth_rx_num] = '5' or [oth_rx_num] = '6' or [oth_rx_num] = '7'**	**Please list the second medication prescribed.**	**text, Required**
	50	**oth_rx_3**<br><br>**Show the field ONLY if: [oth_rx_num] = '3' or [oth_rx_num] = '4' or [oth_rx_num] = '5' or [oth_rx_num] = '6' or [oth_rx_num] = '7'**	**Please list the third medication prescribed.**	**text, Required**
	51	**oth_rx_4**<br><br>**Show the field ONLY if: [oth_rx_num] = '4' or [oth_rx_num] = '5' or [oth_rx_num] = '6' or [oth_rx_num] = '7'**	**Please list the fourth medication prescribed.**	**text, Required**
	52	**oth_rx_5**<br><br>**Show the field ONLY if: [oth_rx_num] = '5' or [oth_rx_num] = '6' or [oth_rx_num] = '7'**	**Please list the fifth medication prescribed.**	**text, Required**
	53	**oth_rx_6**<br><br>**Show the field ONLY if: [oth_rx_num] = '6' or [oth_rx_num] = '7'**	**Please list the sixth medication prescribed.**	**text, Required**
	54	**oth_rx_7**<br><br>**Show the field ONLY if: [oth_rx_num] = '7'**	**Please list the seventh medication prescribed.**	**text, Required**
	55	**ancillary_rx**<br><br>**Show the field ONLY if: [other_rx] = '1'**	**Which of the following classes of medications was the patient prescribed during their E.D. visit?**	**checkbox, Required**<br>1 | ancillary_rx_1 | Anti-emetics<br>2 | ancillary_rx_2 | Anti-diarrheals<br>3 | ancillary_rx_3 | Clonidine<br>4 | ancillary_rx_4 | Non-opioid painkillers<br>5 | ancillary_rx_5 | Opioids (not including buprenorphine)<br>6 | ancillary_rx_6 | Gabapentin<br>7 | ancillary_rx_7 | Psychiatric medications<br>8 | ancillary_rx_8 | Benzodiazepines<br>999 | ancillary_rx_999 | None of the above

© 2021 Herring AA et al. *JAMA Network Open.*

	56	moud_appt	**Section Header:** *Dispo*<br>**Was an appointment or referral made for this patient's substance use disorder?**<br>*Look under "Dispo." Include Pain Clinic, Substance Use Clinic, Cherry Hill, and ED Bridge. Can back-ill from chart.*	**radio, Required**<br>1 Yes<br>0 No
	57	disp	**Disposition**	**radio, Required**<br>1 Home<br>2 Admit<br>3 Eloped/ AMA<br>4 LWOT/ LWBS<br>5 Board & Care<br>6 Transfer (JGPH)<br>7 Transfer (Other hospital)<br>8 Morgue<br>9 Law Enforcement<br>10 Other
	58	disp_oth<br>**Show the field ONLY if:** [disp] = '10'	**If "Other," please specify.**	**text, Required**
	59	phone_1	**Preferred Phone # ******<br>*Enter 999 999 9999 if no contact number.*	**text (phone), Required, Identifier**
	60	disp_date	**Disposition date**	**text (date_mdy), Required, Identifier**
	61	disp_time	**Disposition time**<br>*Military time. xx:xx*	**text (time), Required**
	62	wellsoft_chart_tab_complete	**Section Header:** *Form Status*<br>**Complete?**	**dropdown**<br>0 Incomplete<br>1 Unverified<br>2 Complete
**Instrument:** ED Visit Vitals **(ed_visit_vitals)**				
	63	sbp_triage	**Triage systolic blood pressure**<br>*First recorded SBP. Look in "Vitals."*	**text (integer, Min: 0, Max: 300), Required**
	64	mult_sbp	**Was more than one systolic blood pressure measurement recorded for this visit?**	**radio, Required**<br>1 Yes<br>0 No
	65	sbp_max<br>**Show the field ONLY if:** [mult_sbp] = '1'	**Maximum systolic blood pressure**	**text (integer, Min: 0, Max: 300), Required**
	66	sbp_min<br>**Show the field ONLY if:** [mult_sbp] = '1'	**Minimum systolic blood pressure**	**text (integer, Min: 0, Max: 300), Required**
	67	sbp_last<br>**Show the field ONLY if:** [mult_sbp] = '1'	**Last systolic blood pressure before discharge**<br>*Last recorded SBP.*	**text (integer, Min: 0, Max: 300), Required**
	68	dbp_triage	**Triage diastolic blood pressure**	**text (integer, Min: 0, Max: 300), Required**

© 2021 Herring AA et al. *JAMA Network Open.*

	69	**hr_triage**	**Triage heart rate (bpm)**<br>*First record pulse. Look in "Vitals."*	**text (integer, Min: 0, Max: 300), Required**
	70	**mult_hr**	**Was more than one heart rate recorded for this visit?**	**radio, Required**<br>**1 Yes**<br>**0 No**
	71	**hr_max**<br><br>**Show the field ONLY if: [mult_hr] = '1'**	**Maximum heart rate (bpm)**	**text (integer, Min: 0, Max: 300), Required**
	72	**hr_min**<br><br>**Show the field ONLY if: [mult_hr] = '1'**	**Minimum heart rate (bpm)**	**text (integer, Min: 0, Max: 300), Required**
	73	**hr_last**<br><br>**Show the field ONLY if: [mult_hr] = '1'**	**Last heart rate (bpm) before discharge**<br>*Last recorded pulse.*	**text (integer, Min: 0, Max: 300), Required**
	74	**rr_triage**	**Triage respiratory rate**	**text (integer, Min: 0, Max: 300), Required**
	75	**mult_rr**	**Was more than one respiratory rate recorded for this visit?**	**yesno, Required**<br>**1 Yes**<br>**0 No**
	76	**rr_max**<br><br>**Show the field ONLY if: [mult_rr] = '1'**	**Maximum respiratory rate**	**text, Required**
	77	**rr_min**<br><br>**Show the field ONLY if: [mult_rr] = '1'**	**Minimum respiratory rate**	**text, Required**
	78	**rr_last**<br><br>**Show the field ONLY if: [mult_rr] = '1'**	**Last respiratory rate before discharge**	**text, Required**
	79	**o2_triage**	**Triage oxygen saturation (%)**<br>*Enter numerical value only (no %). First recorded O2 sat. Look in "Vitals."*	**text (integer, Min: 0, Max: 100), Required**
	80	**mult_o2**	**Was more than one oxygen saturation measurement recorded for this visit?**	**yesno, Required**<br>**1 Yes**<br>**0 No**
	81	**o2_max**<br><br>**Show the field ONLY if: [mult_o2] = '1'**	**Maximum oxygen saturation (%)**<br>*Enter numerical value only (no%)*	**text, Required**
	82	**o2_min**<br><br>**Show the field ONLY if: [mult_o2] = '1'**	**Minimum oxygen saturation (%)**<br>*Enter numerical value only (no %)*	**text (integer, Min: 0, Max: 100), Required**
	83	**o2_last**<br><br>**Show the field ONLY if: [mult_o2] = '1'**	**Last oxygen saturation before discharge (%)**<br>*Enter numerical value only (no %). Last recorded O2 sat.*	**text (integer, Min: 0, Max: 100), Required**

© 2021 Herring AA et al. *JAMA Network Open.*

	84	nprs	**Triage Numeric Pain Rating Scale (or any available)**<br>*Choose 999 if no NPRS recorded.*	**dropdown, Required**<br>0 = 0<br>1 = 1<br>2 = 2<br>3 = 3<br>4 = 4<br>5 = 5<br>6 = 6<br>7 = 7<br>8 = 8<br>9 = 9<br>10 = 10<br>999 = 999
	85	mult_nprs	**Was more than one Numeric Pain Rating Scale recorded for this visit?**	**yesno, Required**<br>1 = Yes<br>0 = No
	86	nprs_max<br><br>**Show the field ONLY if: [mult_nprs] = '1'**	**Maximum Numeric Pain Rating Scale**	**dropdown, Required**<br>0 = 0<br>1 = 1<br>2 = 2<br>3 = 3<br>4 = 4<br>5 = 5<br>6 = 6<br>7 = 7<br>8 = 8<br>9 = 9<br>10 = 10
	87	nprs_min<br><br>**Show the field ONLY if: [mult_nprs] = '1'**	**Minimum Numeric Pain Rating Scale**	**dropdown, Required**<br>0 = 0<br>1 = 1<br>2 = 2<br>3 = 3<br>4 = 4<br>5 = 5<br>6 = 6<br>7 = 7<br>8 = 8<br>9 = 9<br>10 = 10

© 2021 Herring AA et al. *JAMA Network Open.*

	88	nprs_last<br><br>Show the field ONLY if:<br>[mult_nprs] = '1'	Last Numeric Pain Rating Scale before discharge	dropdown, Required<br>0 0<br>1 1<br>2 2<br>3 3<br>4 4<br>5 5<br>6 6<br>7 7<br>8 8<br>9 9<br>10 10
	89	ed_visit_vitals_complete	Section Header: *Form Status*<br>Complete?	dropdown<br>0 Incomplete<br>1 Unverified<br>2 Complete
Instrument: Provider Notes and ED Buprenorphine (provider_notes_and_ed_buprenorphine)				
	90	ed_reason_1	Reason for ED visit ****<br>*Complete based on provider notes in Wellsoft chart.*	checkbox, Required<br>1 ed_reason_1_1 Opioid withdrawal<br>2 ed_reason_1_2 Overdose<br>3 ed_reason_1_3 Seeking buprenorphine/detox<br>4 ed_reason_1_4 Non-opioid substance use (EtOH, stimulants)<br>5 ed_reason_1_5 Non-opioid substance withdrawal (EtOH, stimulants)<br>6 ed_reason_1_6 SSTI (abscess, cellulitis)<br>7 ed_reason_1_7 Endocarditis<br>8 ed_reason_1_8 Osteomyelitis<br>9 ed_reason_1_9 Pain management (acute or chronic)<br>10 ed_reason_1_10 Psychiatric symptoms (depression, anxiety, psych med refill, etc)<br>11 ed_reason_1_11 Other medical visit (trauma, med refill, respiratory infection, etc.)

© 2021 Herring AA et al. *JAMA Network Open.*

	91	moud<br><br>Show the field ONLY if:<br>[ed_reason_1(3)] = '1'	If "Seeking buprenorphine/detox," which of the following best describes the patient's ED visit?	radio, Required<br>1 Bup Start<br>2 Bup Lapse Prevention<br>3 Bup Restart<br>999 Unclear
	92	rn_triage	**Section Header:** *Clinical Notes*<br><br>RN triage HPI from Wellsoft<br>*Copy and paste triage RN note.*	notes, Required
	93	referral_yn	Was the patient referred to Highland for this ED visit?	yesno, Required<br>1 Yes<br>0 No
	94	referral<br><br>Show the field ONLY if:<br>[referral_yn] = '1'	Where was the patient referred from?	radio, Required<br>1 Another ED<br>2 Cherry Hill<br>3 Sober living environment<br>4 Family/friends/co-worker<br>5 Signage/advertising<br>6 Primary care doctor<br>7 Counselor<br>8 Law enforcement/drug court<br>9 Other hospital<br>10 Psychiatry/JGP<br>11 Other
	95	referall_oth<br><br>Show the field ONLY if:<br>[referral] = '11'	If "Other," please specify.	text, Required
	96	prebup_sedation	**Section Header:** *Pre-Bup Sedation*<br><br>Which of the following signs or symptoms of sedation were documented before buprenorphine administration?	checkbox, Required<br>1 prebup_sedation_1 Nodding<br>2 prebup_sedation_2 Sleepy, drowsy, diffi culty staying awake<br>3 prebup_sedation_3 Feelings of general fatigue or weakness<br>0 prebup_sedation_0 None of the above

© 2021 Herring AA et al. *JAMA Network Open.*

	97	**prebup_intox**	**Section Header:** *Pre-Bup Intoxication*<br>**Which of the following signs or symptoms of intoxication were documented before buprenorphine administration?**	**checkbox, Required**<br>1 prebup_intox_1 Good mood<br>2 prebup_intox_2 Coasting (Spaced out)<br>3 prebup_intox_3 Pleasant Sick<br>4 prebup_intox_4 Drive (energy)<br>5 prebup_intox_5 High<br>6 prebup_intox_6 Drunken<br>7 prebup_intox_7 Soapbox (talkative)<br>8 prebup_intox_8 Rush<br>9 prebup_intox_9 Friendly<br>10 prebup_intox_10 Relaxed<br>11 prebup_intox_11 Diffi culty concentrating<br>12 prebup_intox_12 Feeling lightheaded or dizzy<br>13 prebup_intox_13 Feeling confused<br>0 prebup_intox_0 None of the above
	98	**prebup_gi**	**Section Header:** *Pre-Bup GI*<br>**Which of the following gastrointestinal signs or symptoms were documented before buprenorphine administration?**	**checkbox, Required**<br>1 prebup_gi_1 Constipation<br>2 prebup_gi_2 Turning of stomach/cramping/abdom pain<br>3 prebup_gi_3 Nausea<br>4 prebup_gi_4 Vomiting<br>5 prebup_gi_5 Diarrhea<br>0 prebup_gi_0 None of the above
	99	**prebup_anxious**	**Section Header:** *Pre-Bup Anxiety*<br>**Which of the following signs or symptoms of anxiety were documented before buprenorphine administration?**	**checkbox, Required**<br>1 prebup_anxious_1 Anxiety, irritability, or agitation<br>2 prebup_anxious_2 Nervousness<br>3 prebup_anxious_3 Tremor<br>4 prebup_anxious_4 Restlessness<br>0 prebup_anxious_0 None of the above

© 2021 Herring AA et al. *JAMA Network Open.*

	100	prebup_vitals	Which of the following abnormal vital signs were documented before buprenorphine administration?	checkbox, Required<br>1 prebup_vitals_1 Hypoxia<br>2 prebup_vitals_2 Hypercarbia<br>3 prebup_vitals_3 Hypotension<br>4 prebup_vitals_4 Bradycardia<br>0 prebup_vitals_0 None of the above
	101	prebup_withdrawal	**Section Header:** *Pre-Bup Withdrawal*<br>Which of the following signs or symptoms of withdrawal were documented before buprenorphine administration?	checkbox, Required<br>1 prebup_withdrawal 1 Yawning<br>2 prebup_withdrawal 2 Gooseflesh skin (chills/piloerectio<br>3 prebup_withdrawal 3 Pulse > 80<br>4 prebup_withdrawal 4 Sweating (diaphoresis)<br>5 prebup_withdrawal 5 Pupil size larger t normal (dilation/mydriasi<br>6 prebup_withdrawal 6 Bone, joint aches, body aches (arthralgias/myal<br>7 prebup_withdrawal 7 Runny nose or tearing (rhinorrhe or lacrimation)<br>0 prebup_withdrawal 0 None of the abov
	102	prebup_misc_sx	**Section Header:** *Pre-Bup Miscellaneous*<br>Which of the following miscellaneous opioid related side effects were documented before buprenorphine administration?	checkbox, Required<br>1 prebup_misc_sx 1 Diffculty passing urine<br>2 prebup_misc_sx 2 Itchiness<br>3 prebup_misc_sx 3 Dry mouth<br>4 prebup_misc_sx 4 Headache<br>5 prebup_misc_sx 5 Unable to walk<br>6 prebup_misc_sx 6 Unable to discharge<br>7 prebup_misc_sx 7 Pupil size smaller than normal (constriction/miosis)<br>0 prebup_misc_sx 0 None of the above
	103	pupils	**Section Header:** *Pre-Bup Pupils*<br>Pupil Quality	radio, Required<br>1 Normal<br>2 Dilated<br>3 Constricted<br>999 Not listed
	104	prebup_pupil_mm	Pupil measurement listed?	yesno, Required<br>1 Yes<br>0 No

© 2021 Herring AA et al. *JAMA Network Open.*

	105	**prebup_pupil_size**<br><br>**Show the field ONLY if: [prebup_pupil_mm] = '1'**	**Please enter the pupil size measured (in mm)**	**text, Required**
	106	**cows**	**Section Header:** *Buprenorphine Administration in the E.D. *****<br><br>**Was an initial COWS score documented before pt received buprenorphine?**	**radio, Required**
	107	**cows_before**<br><br>**Show the field ONLY if: [cows] = '1'**	**What was the COWS score before bup?**	**text, Required**
	108	**bup_ed**	**How many doses of buprenorphine did the pt receive in the ED?**	**radio, Required**
	109	**firstbup**<br><br>**Show the field ONLY if: [bup_ed] = '1' or [bup_ed] = '2' or [bup_ed] = '3' or [bup_ed] = '4' or [bup_ed] = '5' or [bup_ed] = '6' or [bup_ed] = '7'**	**First bup dose**	**radio, Required**
	110	**firstbup_oth**<br><br>**Show the field ONLY if: [firstbup] = '14'**	**If "Other," please specify.**	**text, Required**
	111	**firstbup_time**<br><br>**Show the field ONLY if: [bup_ed] = '1' or [bup_ed] = '2' or [bup_ed] = '3' or [bup_ed] = '4' or [bup_ed] = '5' or [bup_ed] = '6' or [bup_ed] = '7'**	**What date and time was this dose administered?**	**text (datetime_mdy), Required**

© 2021 Herring AA et al. *JAMA Network Open.*

1	Yes
0	No

0	0
1	1
2	2
3	3
4	4
5	5
6	6
7	7

1	0.3 mg IV
2	2 mg SL
3	4 mg SL
4	6 mg SL
5	8 mg SL
6	10 mg SL
7	12 mg SL
8	14 mg SL
9	16 mg SL
10	24 mg SL
11	32 mg SL
12	10mcgpatch
13	20mcgpatch
14	Other

© 2021 Herring AA et al. *JAMA Network Open.*

	112	**bup_admit_1**<br><br>**Show the field ONLY if: [bup_ed] = '1' or [bup_ed] = '2' or [bup_ed] = '3' or [bup_ed] = '4' or [bup_ed] = '5' or [bup_ed] = '6' or [bup_ed] = '7'**	**Was this dose of buprenorphine ordered through Soarian Clinicals?**	**yesno, Required**<br>1 Yes<br>0 No
	113	**post_bup_pt**<br><br>**Show the field ONLY if: [bup_ed] = '1' or [bup_ed] = '2' or [bup_ed] = '3' or [bup_ed] = '4' or [bup_ed] = '5' or [bup_ed] = '6' or [bup_ed] = '7'**	**Does the patient feel same, better, or worse within 90 minutes after this buprenorphine administration?**<br>*Remember to look in "Vitals" for a decrease/increase in pain.*	**radio, Required**<br>1 Same<br>2 Better<br>3 Worse<br>999 No documentation or unclear
	114	**secondbup**<br><br>**Show the field ONLY if: [bup_ed] = '2' or [bup_ed] = '3' or [bup_ed] = '4' or [bup_ed] = '5' or [bup_ed] = '6' or [bup_ed] = '7'**	**Second bup dose**	**radio, Required**<br>1 0.3 mg IV<br>2 2 mg SL<br>3 4 mg SL<br>4 6 mg SL<br>5 8 mg SL<br>6 10 mg SL<br>7 12 mg SL<br>8 14 mg SL<br>9 16 mg SL<br>10 24 mg SL<br>11 32 mg SL<br>12 10 mcg patch<br>13 20 mcg patch<br>14 Other
	115	**secondbup_oth**<br><br>**Show the field ONLY if: [secondbup] = '14'**	**If "Other," please specify.**	**text, Required**
	116	**secondbup_time**<br><br>**Show the field ONLY if: [bup_ed] = '2' or [bup_ed] = '3' or [bup_ed] = '4' or [bup_ed] = '5' or [bup_ed] = '6' or [bup_ed] = '7'**	**What date and time was this dose administered?**	**text (datetime_mdy), Required**
	117	**bup_admit_2**<br><br>**Show the field ONLY if: [bup_ed] = '2' or [bup_ed] = '3' or [bup_ed] = '4' or [bup_ed] = '5' or [bup_ed] = '6' or [bup_ed] = '7'**	**Was this dose of buprenorphine ordered through Soarian Clinicals?**	**yesno, Required**<br>1 Yes<br>0 No

© 2021 Herring AA et al. *JAMA Network Open.*

	118	**post_bup_pt_2**<br>**Show the field ONLY if: [bup_ed] = '2' or [bup_ed] = '3' or [bup_ed] = '4' or [bup_ed] = '5' or [bup_ed] = '6' or [bup_ed] = '7'**	**Does the patient feel same, better, or worse within 90 minutes after this buprenorphine administration?**<br>*Remember to look in "Vitals" for a decrease/increase in pain.*	**radio, Required**<br>1 Same<br>2 Better<br>3 Worse<br>999 No documentation or unclear
	119	**thirdbup**<br>**Show the field ONLY if: [bup_ed] = '3' or [bup_ed] = '4' or [bup_ed] = '5' or [bup_ed] = '6' or [bup_ed] = '7'**	**Third bup dose**	**radio, Required**<br>1 0.3 mg IV<br>2 2 mg SL<br>3 4 mg SL<br>4 6 mg SL<br>5 8 mg SL<br>6 10 mg SL<br>7 12 mg SL<br>8 14 mg SL<br>9 16 mg SL<br>10 24 mg SL<br>11 32 mg SL<br>12 10 mcg patch<br>13 20 mcg patch<br>14 Other
	120	**thirdbup_oth**<br>**Show the field ONLY if: [thirdbup] = '14'**	**If "Other," please specify.**	**text, Required**
	121	**thirdbup_time**<br>**Show the field ONLY if: [bup_ed] = '3' or [bup_ed] = '4' or [bup_ed] = '5' or [bup_ed] = '6' or [bup_ed] = '7'**	**What date and time was this dose administered?**	**text (datetime_mdy), Required**
	122	**bup_admit_3**<br>**Show the field ONLY if: [bup_ed] = '3' or [bup_ed] = '4' or [bup_ed] = '5' or [bup_ed] = '6' or [bup_ed] = '7'**	**Was this dose of buprenorphine ordered through Soarian Clinicals?**	**yesno, Required**<br>1 Yes<br>0 No
	123	**post_bup_pt_3**<br>**Show the field ONLY if: [bup_ed] = '3' or [bup_ed] = '4' or [bup_ed] = '5' or [bup_ed] = '6' or [bup_ed] = '7'**	**Does the patient feel same, better, or worse within 90 minutes after this buprenorphine administration?**<br>*Remember to look in "Vitals" for a decrease/increase in pain.*	**radio, Required**<br>1 Same<br>2 Better<br>3 Worse<br>999 No documentation or unclear

© 2021 Herring AA et al. *JAMA Network Open.*

	124	**fourthbup**<br>**Show the field ONLY if: [bup_ed] = '4' or [bup_ed] = '5' or [bup_ed] = '6' or [bup_ed] = '7'**	**Fourth bup dose**	**radio, Required**<br>1 0.3 mg IV<br>2 2 mg SL<br>3 4 mg SL<br>4 6 mg SL<br>5 8 mg SL<br>6 10 mg SL<br>7 12 mg SL<br>8 14 mg SL<br>9 16 mg SL<br>10 24 mg SL<br>11 32 mg SL<br>12 10 mcg patch<br>13 20 mcg patch<br>14 Other
	125	**fourthbup_oth**<br>**Show the field ONLY if: [fourthbup] = '14'**	**If "Other," please specify.**	**text, Required**
	126	**fourthbup_time**<br>**Show the field ONLY if: [bup_ed] = '4' or [bup_ed] = '5' or [bup_ed] = '6' or [bup_ed] = '7'**	**What date and time was this dose administered?**	**text (datetime_mdy), Required**
	127	**bup_admit_4**<br>**Show the field ONLY if: [bup_ed] = '4' or [bup_ed] = '5' or [bup_ed] = '6' or [bup_ed] = '7'**	**Was this dose of buprenorphine ordered through Soarian Clinicals?**	**yesno, Required**<br>1 Yes<br>0 No
	128	**post_bup_pt_4**<br>**Show the field ONLY if: [bup_ed] = '4' or [bup_ed] = '5' or [bup_ed] = '6' or [bup_ed] = '7'**	**Does the patient feel same, better, or worse within 90 minutes after this buprenorphine administration?**<br>*Remember to look in "Vitals" for a decrease/increase in pain.*	**radio, Required**<br>1 Same<br>2 Better<br>3 Worse<br>999 No documentation or unclear

© 2021 Herring AA et al. *JAMA Network Open.*

	129	fifthbup<br><br>Show the field ONLY if: [bup_ed] = '5' or [bup_ed] = '6' or [bup_ed] = '7'	Fifth bup dose	radio, Required<br>1 0.3 mg IV<br>2 2 mg SL<br>3 4 mg SL<br>4 6 mg SL<br>5 8 mg SL<br>6 10 mg SL<br>7 12 mg SL<br>8 14 mg SL<br>9 16 mg SL<br>10 24 mg SL<br>11 32 mg SL<br>12 10 mcg patch<br>13 20 mcg patch<br>14 Other
	130	fifthbup_oth<br><br>Show the field ONLY if: [fifthbup] = '14'	If "Other," please specify.	text, Required
	131	fifthbup_time<br><br>Show the field ONLY if: [bup_ed] = '5' or [bup_ed] = '6' or [bup_ed] = '7'	What date and time was this dose administered?	text (datetime_mdy), Required
	132	bup_admit_5<br><br>Show the field ONLY if: [bup_ed] = '5' or [bup_ed] = '6' or [bup_ed] = '7'	Was this dose of buprenorphine ordered through Soarian Clinicals?	yesno, Required<br>1 Yes<br>0 No
	133	post_bup_pt_5<br><br>Show the field ONLY if: [bup_ed] = '5' or [bup_ed] = '6' or [bup_ed] = '7'	Does the patient feel same, better, or worse within 90 minutes after this buprenorphine administration?<br>*Remember to look in "Vitals" for a decrease/increase in pain.*	radio, Required<br>1 Same<br>2 Better<br>3 Worse<br>999 No documentation or unclear

© 2021 Herring AA et al. *JAMA Network Open.*

	134	**sixthbup**<br><br>**Show the field ONLY if: [bup_ed] = '6' or [bup_ed] = '7'**	**Sixth bup dose**	**radio, Required**<br>1 — 0.3 mg IV<br>2 — 2 mg SL<br>3 — 4 mg SL<br>4 — 6 mg SL<br>5 — 8 mg SL<br>6 — 10 mg SL<br>7 — 12 mg SL<br>8 — 14 mg SL<br>9 — 16 mg SL<br>10 — 24 mg SL<br>11 — 32 mg SL<br>12 — 10 mcg patch<br>13 — 20 mcg patch<br>14 — Other
	135	**sixthbup_oth**<br><br>**Show the field ONLY if: [sixthbup] = '14'**	**If "Other," please specify.**	**text, Required**
	136	**sixthbup_time**<br><br>**Show the field ONLY if: [bup_ed] = '6' or [bup_ed] = '7'**	**What date and time was this dose administered?**	**text (datetime_mdy), Required**
	137	**bup_admit_6**<br><br>**Show the field ONLY if: [bup_ed] = '6' or [bup_ed] = '7'**	**Was this dose of buprenorphine ordered through Soarian Clinicals?**	**yesno, Required**<br>1 — Yes<br>0 — No
	138	**post_bup_pt_6**<br><br>**Show the field ONLY if: [bup_ed] = '6' or [bup_ed] = '7'**	**Does the patient feel same, better, or worse within 90 minutes after this buprenorphine administration?**<br>*Remember to look in "Vitals" for a decrease/increase in pain.*	**radio, Required**<br>1 — Same<br>2 — Better<br>3 — Worse<br>999 — No documentation or unclear

© 2021 Herring AA et al. *JAMA Network Open.*

	139	seventhbup<br><br>Show the field ONLY if:<br>[bup_ed] = '7'	Seventh bup dose	radio, Required<br>1 0.3 mg IV<br>2 2 mg SL<br>3 4 mg SL<br>4 6 mg SL<br>5 8 mg SL<br>6 10 mg SL<br>7 12 mg SL<br>8 14 mg SL<br>9 16 mg SL<br>10 24 mg SL<br>11 32 mg SL<br>12 10 mcg patch<br>13 20 mcg patch<br>14 Other
	140	seventhbup_oth<br><br>Show the field ONLY if:<br>[seventhbup] = '14'	If "Other," please specify.	text, Required
	141	seventhbup_time<br><br>Show the field ONLY if:<br>[bup_ed] = '7'	What date and time was this dose administered?	text (datetime_mdy), Required
	142	bup_admit_7<br><br>Show the field ONLY if:<br>[bup_ed] = '7'	Was this dose of buprenorphine ordered through Soarian Clinicals?	yesno, Required<br>1 Yes<br>0 No
	143	post_bup_pt_7<br><br>Show the field ONLY if:<br>[bup_ed] = '7'	Does the patient feel same, better, or worse within 90 minutes after this buprenorphine administration?<br>*Remember to look in "Vitals" for a decrease/increase in pain.*	radio, Required<br>1 Same<br>2 Better<br>3 Worse<br>999 No documentation or unclear
	144	buptotalcheck_1<br><br>Show the field ONLY if:<br>[bup_ed] = '1'	You have indicated bup administration of [firstbup]. Is this correct?	yesno, Required<br>1 Yes<br>0 No
	145	buptotalcheck_2<br><br>Show the field ONLY if:<br>[bup_ed] = '2'	You have indicated bup administration of [firstbup], [secondbup]. Is this correct?	yesno, Required<br>1 Yes<br>0 No
	146	buptotalcheck_3<br><br>Show the field ONLY if:<br>[bup_ed] = '3'	You have indicated bup administration of [firstbup], [secondbup], [thirdbup]. Is this correct?	yesno, Required<br>1 Yes<br>0 No

© 2021 Herring AA et al. *JAMA Network Open.*

	147	buptotalcheck_4<br><br>Show the field ONLY if:<br>[bup_ed] = '4'	You have indicated bup administration of [firstbup], [secondbup], [thirdbup], [fourthbup]. Is this correct?	yesno, Required<br>1 Yes<br>0 No
	148	buptotalcheck_5<br><br>Show the field ONLY if:<br>[bup_ed] = '5'	You have indicated bup administration of [firstbup], [secondbup], [thirdbup], [fourthbup], [fifthbup]. Is this correct?	yesno, Required<br>1 Yes<br>0 No
	149	buptotalcheck_6<br><br>Show the field ONLY if:<br>[bup_ed] = '6'	You have indicated bup administration of [firstbup], [secondbup], [thirdbup], [fourthbup], [fifthbup], [sixthbup]. Is this correct?	yesno, Required<br>1 Yes<br>0 No
	150	buptotalcheck_7<br><br>Show the field ONLY if:<br>[bup_ed] = '7'	You have indicated bup administration of [firstbup], [secondbup], [thirdbup], [fourthbup], [fifthbup], [sixthbup], [seventhbup]. Is this correct?	yesno, Required<br>1 Yes<br>0 No
	151	buptotalfix<br><br>Show the field ONLY if:<br>[buptotalcheck_1] = '0' or [buptotalcheck_2] = '0' or [buptotalcheck_3] = '0' or [buptotalcheck_4] = '0' or [buptotalcheck_5] = '0' or [buptotalcheck_6] = '0' or [buptotalcheck_7] = '0'	Check chart and fix buprenorphine doses.	radio
	152	sumbup_sl<br><br>Show the field ONLY if:<br>[bup_ed] = '1' or [bup_ed] = '2' or [bup_ed] = '3' or [bup_ed] = '4' or [bup_ed] = '5' or [bup_ed] = '6' or [bup_ed] = '7'	Double check chart and above. Add up the total SL bup administered (mg)<br>*Add up all SL doses.*	text, Required
	153	iv_bup	Did the patient receive IV buprenorphine?	yesno, Required<br>1 Yes<br>0 No
	154	sumbup_iv<br><br>Show the field ONLY if:<br>[iv_bup] = '1'	Double check chart and above. Add up the total IV bup administered (mg)<br>*Add up all IV doses.*	text, Required
	155	cows_post<br><br>Show the field ONLY if:<br>[bup_ed] = '1' or [bup_ed] = '2' or [bup_ed] = '3' or [bup_ed] = '4' or [bup_ed] = '5' or [bup_ed] = '6' or [bup_ed] = '7'	Were any COWS scores recorded after pt received buprenorphine in the ED?	radio, Required<br>1 Yes<br>0 No

© 2021 Herring AA et al. *JAMA Network Open.*

	156	**cows_post_number**<br><br>**Show the field ONLY if: [cows_post] = '1'**	**How many COWS scores were recorded after buprenorphine administration?**	**radio, Required**<br>**1 1**<br>**2 2**<br>**3 3**<br>**4 4**<br>**5 5**
	157	**cows_post_1**<br><br>**Show the field ONLY if: [cows_post_number] = '1' or [cows_post_number] = '2' or [cows_post_number] = '3' or [cows_post_number] = '4' or [cows_post_number] = '5'**	**First COWS score post-buprenorphine.**	**text, Required**
	158	**cows_post_time_1**<br><br>**Show the field ONLY if: [cows_post_number] = '1' or [cows_post_number] = '2' or [cows_post_number] = '3' or [cows_post_number] = '4' or [cows_post_number] = '5'**	**Date and time of first COWS score post-buprenorphine.**<br>*Leave blank if unknown.*	**text (datetime_mdy)**
	159	**cows_post_2**<br><br>**Show the field ONLY if: [cows_post_number] = '2' or [cows_post_number] = '3' or [cows_post_number] = '4' or [cows_post_number] ='5'**	**Second COWS score post-buprenorphine.**	**text, Required**
	160	**cows_post_time_2**<br><br>**Show the field ONLY if: [cows_post_number] = '2' or [cows_post_number] = '3' or [cows_post_number] = '4' or [cows_post_number] ='5'**	**Date and time of second COWS score post-buprenorphine.**<br>*Leave blank if unknown.*	**text (datetime_mdy)**
	161	**cows_post_3**<br><br>**Show the field ONLY if: [cows_post_number] = '3' or [cows_post_number] = '4' or [cows_post_number] ='5'**	**Third COWS score post-buprenorphine.**	**text, Required**
	162	**cows_post_time_3**<br><br>**Show the field ONLY if: [cows_post_number] = '3' or [cows_post_number] = '4' or [cows_post_number] ='5'**	**Date and time of third COWS score post-buprenorphine.**<br>*Leave blank if unknown.*	**text (datetime_mdy)**

© 2021 Herring AA et al. *JAMA Network Open.*

	163	cows_post_4<br><br>Show the field ONLY if: [cows_post_number] = '4' or [cows_post_number] = '5'	Fourth COWS score post-buprenorphine.	text, Required
	164	cows_post_time_4<br><br>Show the field ONLY if: [cows_post_number] = '4' or [cows_post_number] = '5'	Date and time of fourth COWS score post-buprenorphine.<br>*Leave blank if unknown.*	text (datetime_mdy)
	165	cows_post_5<br><br>Show the field ONLY if: [cows_post_number] = '5'	Fifth COWS score post-buprenorphine.	text, Required
	166	cows_post_time_5<br><br>Show the field ONLY if: [cows_post_number] = '5'	Date and time of fifth COWS score post-buprenorphine.<br>*Leave blank if unknown.*	text (datetime_mdy)
	167	post_bup	Compare the clinical impression at presentation with the clinical impression after all buprenorphine administrations. At discharge is the patient's overall clinical status:	radio, Required<br>1 Unchanged<br>2 Improved<br>3 Worse<br>999 Can't tell
	168	postbup_ss	Section Header: *Post-Bup Signs and Symptoms*<br>Were any of the signs or symptom listed above documented within 6 hours after the patient received buprenorphine?	yesno, Required<br>1 Yes<br>0 No
	169	postbup_sedation<br><br>Show the field ONLY if: [postbup_ss] ='1'	Section Header: *Post-Bup Sedation*<br>Please review the entire clinical chart and note if any of the following signs or symptoms of sedation were documented within 6 hours after first buprenorphine administration.	checkbox, Required<br>1 postbup_sedation_1 Nodding<br>2 postbup_sedation_2 Sleepy, drowsy, diffculty staying awake<br>3 postbup_sedation_3 Feelings of general fatigue or weakness<br>0 postbup_sedation_0 None of the above
	170	nodding_time_yn<br><br>Show the field ONLY if: [postbup_sedation(1)] = '1'	Did the provider document the time(s) at which nodding was noted?	yesno, Required<br>1 Yes<br>0 No

	171	**nodding_num**<br><br>**Show the field ONLY if: [nodding_time_yn] = '1'**	**How many instances of nodding were documented with timestamps within 6 hours after first buprenorphine administration?**	**radio, Required**<br>0 0<br>1 1<br>2 2<br>3 3<br>4 4<br>5 5
	172	**nod_time1**<br><br>**Show the field ONLY if: [nodding_num] = '1' or [nodding_num] = '2' or [nodding_num] = '3' or [nodding_num] = '4' or [nodding_num] = '5'**	**Record the date and time of the first instance of nodding**	**text (datetime_mdy), Required**
	173	**nod_time2**<br><br>**Show the field ONLY if: [nodding_num] = '2' or [nodding_num] = '3' or [nodding_num] = '4' or [nodding_num] = '5'**	**Record the date and time of the second instance of nodding**	**text (datetime_mdy), Required**
	174	**nod_time3**<br><br>**Show the field ONLY if: [nodding_num] = '3' or [nodding_num] = '4' or [nodding_num] = '5'**	**Record the date and time of the third instance of nodding**	**text (datetime_mdy), Required**
	175	**nod_time4**<br><br>**Show the field ONLY if: [nodding_num] = '4' or [nodding_num] = '5'**	**Record the date and time of the fourth instance of nodding**	**text (datetime_mdy), Required**
	176	**nod_time5**<br><br>**Show the field ONLY if: [nodding_num] = '5'**	**Record the date and time of the fifth instance of nodding**	**text (datetime_mdy), Required**
	177	**sleepy_time_yn**<br><br>**Show the field ONLY if: [postbup_sedation(2)] = '1'**	**Did the provider document the time(s) at which sleepiness, drowsiness, or diffculty staying awake were noted?**	**yesno, Required**<br>1 Yes<br>0 No
	178	**sleepy_num**<br><br>**Show the field ONLY if: [sleepy_time_yn] = '1'**	**How many instances of sleepiness, drowsiness, or diffculty staying awake were documented within 6 hours after first buprenorphine administration?**	**radio, Required**<br>0 0<br>1 1<br>2 2<br>3 3<br>4 4<br>5 5

© 2021 Herring AA et al. *JAMA Network Open.*

	179	sleepy_time1<br><br>Show the field ONLY if:<br>[sleepy_num] = '1' or [sleepy_num] = '2' or [sleepy_num] = '3' or [sleepy_num] = '4' or [sleepy_num] = '5'	Record the date and time of the first instance of sleepiness, drowsiness, or diffculty staying awake.	text (datetime_mdy), Required
	180	sleepy_time2<br><br>Show the field ONLY if:<br>[sleepy_num] = '2' or [sleepy_num] = '3' or [sleepy_num] = '4' or [sleepy_num] = '5'	Record the date and time of the second instance of sleepiness, drowsiness, or diffculty staying awake.	text (datetime_mdy), Required
	181	sleepy_time3<br><br>Show the field ONLY if:<br>[sleepy_num] = '3' or [sleepy_num] = '4' or [sleepy_num] = '5'	Record the date time of the third instance of sleepiness, drowsiness, or diffculty staying awake.	text (datetime_mdy), Required
	182	sleepy_time4<br><br>Show the field ONLY if:<br>[sleepy_num] = '4' or [sleepy_num] = '5'	Record the date and time of the fourth instance of sleepiness, drowsiness, or diffculty staying awake.	text (datetime_mdy), Required
	183	sleepy_time5<br><br>Show the field ONLY if:<br>[sleepy_num] = '5'	Record the date and time of the fifth instance of sleepiness, drowsiness, or diffculty staying awake.	text (datetime_mdy), Required
	184	fatigue_time_yn<br><br>Show the field ONLY if:<br>[postbup_sedation(3)] = '1'	Did the provider document the time(s) at which general fatigue or feelings of weakness were noted?	yesno, Required<br>1 Yes<br>0 No
	185	fatigue_num<br><br>Show the field ONLY if:<br>[fatigue_time_yn] = '1'	How many instances of general fatigue or feelings of weakness were documented with timestamps within 6 hours after first buprenorphine administration?	radio, Required<br>0 0<br>1 1<br>2 2<br>3 3<br>4 4<br>5 5
	186	fatigue_time1<br><br>Show the field ONLY if:<br>[fatigue_num] = '1' or [fatigue_num] = '2' or [fatigue_num] = '3' or [fatigue_num] = '4' or [fatigue_num] = '5'	Record the date and time of the first instance of general fatigue or weakness.	text (datetime_mdy), Required

	187	**fatigue_time2**<br><br>**Show the field ONLY if: [fatigue_num] = '2' or [fatigue_num] = '3' or [fatigue_num] = '4' or [fatigue_num] = '5'**	**Record the date and time of the second instance of general fatigue or weakness.**	**text (datetime_mdy), Required**
	188	**fatigue_time3**<br><br>**Show the field ONLY if: [fatigue_num] = '3' or [fatigue_num] = '4' or [fatigue_num] = '5'**	**Record the date and time of the third instance of general fatigue or weakness.**	**text (datetime_mdy), Required**
	189	**fatigue_time4**<br><br>**Show the field ONLY if: [fatigue_num] = '4' or [fatigue_num] = '5'**	**Record the date and time of the fourth instance of general fatigue or weakness.**	**text (datetime_mdy), Required**
	190	**fatigue_time5**<br><br>**Show the field ONLY if: [fatigue_num] = '5'**	**Record the date and time of the fifth instance of general fatigue or weakness.**	**text (datetime_mdy), Required**
	191	**postbup_intox**<br><br>**Show the field ONLY if: [postbup_ss] = '1'**	**Section Header:** *Post-Bup Intoxication*<br><br>**Please review the entire clinical chart and note if any of the following signs or symptoms of intoxication were documented within 6 hours after first buprenorphine administration.**	**checkbox, Required**<br>1 | postbup_intox_1 | Good mood<br>2 | postbup_intox_2 | Coasting (Spaced out)<br>3 | postbup_intox_3 | Pleasant Sick<br>4 | postbup_intox_4 | Drive (energy)<br>5 | postbup_intox_5 | High<br>6 | postbup_intox_6 | Drunken<br>7 | postbup_intox_7 | Soapbox (talkative)<br>8 | postbup_intox_8 | Rush<br>9 | postbup_intox_9 | Friendly<br>10 | postbup_intox_10 | Relaxed<br>11 | postbup_intox_11 | Diffculty concentrating<br>12 | postbup_intox_12 | Feeling lightheaded or dizzy<br>13 | postbup_intox_13 | Feeling confused<br>0 | postbup_intox_0 | None of the above
	192	**mood_time_yn**<br><br>**Show the field ONLY if: [postbup_intox(1)]='1'**	**Did the provider document the time(s) at which good mood was noted?**	**yesno, Required**<br>1 | Yes<br>0 | No

	193	**mood_num**<br><br>**Show the field ONLY if: [mood_time_yn] = '1'**	**How many instances of good mood were documented with timestamps within 6 hours after first buprenorphine administration?**	**radio, Required**<br>0 0<br>1 1<br>2 2<br>3 3<br>4 4<br>5 5
	194	**mood_time1**<br><br>**Show the field ONLY if: [mood_num] = '1' or [mood_num] = '2' or [mood_num] = '3' or [mood_num] = '4' or [mood_num] = '5'**	**Record the date and time of the first instance of good mood.**	**text (datetime_mdy), Required**
	195	**mood_time2**<br><br>**Show the field ONLY if: [mood_num] = '2' or [mood_num] = '3' or [mood_num] = '4' or [mood_num] = '5'**	**Record the date and time of the second instance of good mood.**	**text (datetime_mdy), Required**
	196	**mood_time3**<br><br>**Show the field ONLY if: [mood_num] = '3' or [mood_num] = '4' or [mood_num] = '5'**	**Record the date and time of third instance of good mood.**	**text (datetime_mdy), Required**
	197	**mood_time4**<br><br>**Show the field ONLY if: [mood_num] = '4' or [mood_num] = '5'**	**Record the date and time of fourth instance of good mood.**	**text (datetime_mdy), Required**
	198	**mood_time5**<br><br>**Show the field ONLY if: [mood_num] = '5'**	**Record the date and time of fifth instance of good mood.**	**text (datetime_mdy), Required**
	199	**coasting_time_yn**<br><br>**Show the field ONLY if: [postbup_intox(2)]='1'**	**Did the provider document the time(s) at which coasting was noted?**	**yesno, Required**<br>1 Yes<br>0 No
	200	**coasting_num**<br><br>**Show the field ONLY if: [coasting_time_yn] = '1'**	**How many instances of coasting were documented with timestamps within 6 hours after first buprenorphine administration?**	**radio, Required**<br>0 0<br>1 1<br>2 2<br>3 3<br>4 4<br>5 5

© 2021 Herring AA et al. *JAMA Network Open.*

	201	**coasting_time1**<br><br>**Show the field ONLY if: [coasting_num] = '1' or [coasting_num] = '2' or [coasting_num] = '3' or [coasting_num] = '4' or [coasting_num] = '5'**	**Record the date and time of the first instance of coasting.**	**text (datetime_mdy), Required**
	202	**coasting_time2**<br><br>**Show the field ONLY if: [coasting_num] = '2' or [coasting_num] = '3' or [coasting_num] = '4' or [coasting_num] = '5'**	**Record the date and time of the second instance of coasting.**	**text (datetime_mdy), Required**
	203	**coasting_time3**<br><br>**Show the field ONLY if: [coasting_num] = '3' or [coasting_num] = '4' or [coasting_num] = '5'**	**Record the date and time of the third instance of coasting.**	**text (datetime_mdy), Required**
	204	**coasting_time4**<br><br>**Show the field ONLY if: [coasting_num] = '4' or [coasting_num] = '5'**	**Record the date and time of the fourth instance of coasting.**	**text (datetime_mdy), Required**
	205	**coasting_time5**<br><br>**Show the field ONLY if: [coasting_num] = '5'**	**Record the date and time of the fifth instance of coasting.**	**text (datetime_mdy), Required**
	206	**pleasant_time_yn**<br><br>**Show the field ONLY if: [postbup_intox(3)]='1'**	**Did the provider document the time(s) at which pleasant sickness was noted?**	**yesno, Required**<br>**1 Yes**<br>**0 No**
	207	**pleasant_num**<br><br>**Show the field ONLY if: [pleasant_time_yn] = '1'**	**How many instances of pleasant sickness were documented with timestamps within 6 hours after first buprenorphine administration?**	**radio, Required**<br>**0 0**<br>**1 1**<br>**2 2**<br>**3 3**<br>**4 4**<br>**5 5**
	208	**pleasant_time1**<br><br>**Show the field ONLY if: [pleasant_num] = '1' or [pleasant_num] = '2' or [pleasant_num] = '3' or [pleasant_num] = '4' or [pleasant_num] = '5'**	**Record the date and time of the first instance of pleasant sickness.**	**text (datetime_mdy), Required**
	209	**pleasant_time2**<br><br>**Show the field ONLY if: [pleasant_num] = '2' or [pleasant_num] = '3' or [pleasant_num] = '4' or [pleasant_num] = '5'**	**Record the date and time of the second instance of pleasant sickness.**	**text (datetime_mdy), Required**

© 2021 Herring AA et al. *JAMA Network Open.*

	210	**pleasant_time3**<br><br>**Show the field ONLY if: [pleasant_num] = '3' or [pleasant_num] = '4' or [pleasant_num] = '5'**	**Record the date and time of the third instance of pleasant sickness.**	**text (datetime_mdy), Required**
	211	**pleasant_time4**<br><br>**Show the field ONLY if: [pleasant_num] = '4' or [pleasant_num] = '5'**	**Record the date and time of the fourth instance of pleasant sickness.**	**text (datetime_mdy), Required**
	212	**pleasant_time5**<br><br>**Show the field ONLY if: [pleasant_num] = '5'**	**Record the date and time of the fifth instance of pleasant sickness.**	**text (datetime_mdy), Required**
	213	**drive_time_yn**<br><br>**Show the field ONLY if: [postbup_intox(4)]='1'**	**Did the provider document the time(s) at which having drive was noted?**	**yesno, Required**<br>1 Yes<br>0 No
	214	**drive_num**<br><br>**Show the field ONLY if: [drive_time_yn] = '1'**	**How many instances of drive were documented with timestamps within 6 hours after first buprenorphine administration?**	**radio, Required**<br>0 0<br>1 1<br>2 2<br>3 3<br>4 4<br>5 5
	215	**drive_time1**<br><br>**Show the field ONLY if: [drive_num] = '1' or [drive_num] = '2' or [drive_num] = '3' or [drive_num] = '4' or [drive_num] = '5'**	**Record the date and time of the first instance of drive.**	**text (datetime_mdy), Required**
	216	**drive_time2**<br><br>**Show the field ONLY if: [drive_num] = '2' or [drive_num] = '3' or [drive_num] = '4' or [drive_num] = '5'**	**Record the date and time of the second instance of drive.**	**text (datetime_mdy), Required**
	217	**drive_time3**<br><br>**Show the field ONLY if: [drive_num] = '3' or [drive_num] = '4' or [drive_num] = '5'**	**Record the date and time of the third instance of drive.**	**text (datetime_mdy), Required**
	218	**drive_time4**<br><br>**Show the field ONLY if: [drive_num] = '4' or [drive_num] = '5'**	**Record the date and time of the fourth instance of drive.**	**text (datetime_mdy), Required**
	219	**drive_time5**<br><br>**Show the field ONLY if: [drive_num] = '5'**	**Record the date and time of the fifth instance of drive.**	**text (datetime_mdy), Required**

© 2021 Herring AA et al. *JAMA Network Open.*

	220	**high_time_yn**<br><br>**Show the field ONLY if: [postbup_intox(5)]='1'**	**Did the provider document the time(s) at which being high was noted?**	**yesno, Required**<br>1 Yes<br>0 No
	221	**high_num**<br><br>**Show the field ONLY if: [high_time_yn] = '1'**	**How many instances of being high were documented with timestamps within 6 hours after first buprenorphine administration?**	**radio, Required**<br>0 0<br>1 1<br>2 2<br>3 3<br>4 4<br>5 5
	222	**high_time1**<br><br>**Show the field ONLY if: [high_num] = '1' or [high_num] = '2' or [high_num] = '3' or [high_num] = '4' or [high_num] = '5'**	**Record the date and time of the first instance of being high.**	**text (datetime_mdy), Required**
	223	**high_time2**<br><br>**Show the field ONLY if: [high_num] = '2' or [high_num] = '3' or [high_num] = '4' or [high_num] = '5'**	**Record the date and time of the second instance of being high.**	**text (datetime_mdy), Required**
	224	**high_time3**<br><br>**Show the field ONLY if: [high_num] = '3' or [high_num] = '4' or [high_num] = '5'**	**Record the date and time of the third instance of being high.**	**text (datetime_mdy), Required**
	225	**high_time4**<br><br>**Show the field ONLY if: [high_num] = '4' or [high_num] = '5'**	**Record the date and time of the fourth instance of being high.**	**text (datetime_mdy), Required**
	226	**high_time5**<br><br>**Show the field ONLY if: [high_num] = '5'**	**Record the date and time of the fifth instance of being high.**	**text (datetime_mdy), Required**
	227	**drunk_time_yn**<br><br>**Show the field ONLY if: [postbup_intox(6)]='1'**	**Did the provider document the time(s) at which drunkenness was noted?**	**yesno, Required**<br>1 Yes<br>0 No
	228	**drunk_num**<br><br>**Show the field ONLY if: [drunk_time_yn] = '1'**	**How many instances of drunkenness were documented with timestamps within 6 hours after first buprenorphine administration?**	**radio, Required**<br>0 0<br>1 1<br>2 2<br>3 3<br>4 4<br>5 5

© 2021 Herring AA et al. *JAMA Network Open.*

	229	**drunk_time1**<br><br>**Show the field ONLY if: [drunk_num] = '1' or [drunk_num] = '2' or [drunk_num] = '3' or [drunk_num] = '4' or [drunk_num] = '5'**	**Record the date and time of the first instance of drunkenness.**	**text (datetime_mdy), Required**
	230	**drunk_time2**<br><br>**Show the field ONLY if: [drunk_num] = '2' or [drunk_num] = '3' or [drunk_num] = '4' or [drunk_num] = '5'**	**Record the date and time of the second instance of drunkenness.**	**text (datetime_mdy), Required**
	231	**drunk_time3**<br><br>**Show the field ONLY if: [drunk_num] = '3' or [drunk_num] = '4' or [drunk_num] = '5'**	**Record the date and time of the third instance of drunkenness.**	**text (datetime_mdy), Required**
	232	**drunk_time4**<br><br>**Show the field ONLY if: [drunk_num] = '4' or [drunk_num] = '5'**	**Record the date and time of the fourth instance of drunkenness.**	**text (datetime_mdy), Required**
	233	**drunk_time5**<br><br>**Show the field ONLY if: [drunk_num] = '5'**	**Record the date and time of the fifth instance of drunkenness.**	**text (datetime_mdy), Required**
	234	**soapbox_time_yn**<br><br>**Show the field ONLY if: [postbup_intox(7)]='1'**	**Did the provider document the time(s) at which talkativeness (soapbox) was noted?**	**yesno, Required**<br>**1 Yes**<br>**0 No**
	235	**soapbox_num**<br><br>**Show the field ONLY if: [soapbox_time_yn] = '1'**	**How many instances of talkativeness were documented with timestamps within 6 hours after first buprenorphine administration?**	**radio, Required**<br>**0 0**<br>**1 1**<br>**2 2**<br>**3 3**<br>**4 4**<br>**5 5**
	236	**soapbox_time1**<br><br>**Show the field ONLY if: [soapbox_num] = '1' or [soapbox_num] = '2' or [soapbox_num] = '3' or [soapbox_num] = '4' or [soapbox_num] = '5'**	**Record the date and time of the first instance of talkativeness.**	**text (datetime_mdy), Required**
	237	**soapbox_time2**<br><br>**Show the field ONLY if: [soapbox_num] = '2' or [soapbox_num] = '3' or [soapbox_num] = '4' or [soapbox_num] = '5'**	**Record the date and time of the second instance of talkativeness.**	**text (datetime_mdy), Required**

© 2021 Herring AA et al. *JAMA Network Open.*

	238	**soapbox_time3**<br><br>**Show the field ONLY if: [soapbox_num] = '3' or [soapbox_num] = '4' or [soapbox_num] = '5'**	**Record the date and time of the third instance of talkativeness.**	**text (datetime_mdy), Required**
	239	**soapbox_time4**<br><br>**Show the field ONLY if: [soapbox_num] = '4' or [soapbox_num] = '5'**	**Record the date and time of the fourth instance of talkativeness.**	**text (datetime_mdy), Required**
	240	**soapbox_time5**<br><br>**Show the field ONLY if: [soapbox_num] = '5'**	**Record the date and time of the fifth instance of talkativeness.**	**text (datetime_mdy), Required**
	241	**rush_time_yn**<br><br>**Show the field ONLY if: [postbup_intox(8)]='1'**	**Did the provider document the time(s) at which rush was noted?**	**yesno, Required**<br>1 Yes<br>0 No
	242	**rush_num**<br><br>**Show the field ONLY if: [rush_time_yn] = '1'**	**How many instances of rush were documented with timestamps within 6 hours after first buprenorphine administration?**	**radio, Required**<br>0 0<br>1 1<br>2 2<br>3 3<br>4 4<br>5 5
	243	**rush_time1**<br><br>**Show the field ONLY if: [rush_num] = '1' or [rush_num] = '2' or [rush_num] = '3' or [rush_num] = '4' or [rush_num] = '5'**	**Record the date and time of the first instance of rush.**	**text (datetime_mdy), Required**
	244	**rush_time2**<br><br>**Show the field ONLY if: [rush_num] = '2' or [rush_num] = '3' or [rush_num] = '4' or [rush_num] = '5'**	**Record the date and time of the second instance of rush.**	**text (datetime_mdy), Required**
	245	**rush_time3**<br><br>**Show the field ONLY if: [rush_num] = '3' or [rush_num] = '4' or [rush_num] = '5'**	**Record the date and time of the third instance of rush.**	**text (datetime_mdy), Required**
	246	**rush_time4**<br><br>**Show the field ONLY if: [rush_num] = '4' or [rush_num] = '5'**	**Record the date and time of the fourth instance of rush.**	**text (datetime_mdy), Required**
	247	**rush_time5**<br><br>**Show the field ONLY if: [rush_num] = '5'**	**Record the date and time of the fifth instance of rush.**	**text (datetime_mdy), Required**

© 2021 Herring AA et al. *JAMA Network Open.*

	248	**friendly_time_yn**<br><br>Show the field ONLY if: [postbup_intox(9)]='1'	Did the provider document the time(s) at which friendliness was noted?	yesno, Required<br>1 Yes<br>0 No
	249	**friendly_num**<br><br>Show the field ONLY if: [friendly_time_yn]='1'	How many instances of friendliness were documented with timestamps within 6 hours after first buprenorphine administration?	radio, Required<br>0 0<br>1 1<br>2 2<br>3 3<br>4 4<br>5 5
	250	**friendly_time1**<br><br>Show the field ONLY if: [friendly_num] = '1' or [friendly_num] = '2' or [friendly_num] = '3' or [friendly_num] = '4' or [friendly_num] = '5'	Record the date and time of the first instance of friendliness.	text (datetime_mdy), Required
	251	**friendly_time2**<br><br>Show the field ONLY if: [friendly_num] = '2' or [friendly_num] = '3' or [friendly_num] = '4' or [friendly_num] = '5'	Record the date and time of the second instance of friendliness.	text (datetime_mdy), Required
	252	**friendly_time3**<br><br>Show the field ONLY if: [friendly_num] = '3' or [friendly_num] = '4' or [friendly_num] = '5'	Record the date and time of the third instance of friendliness.	text (datetime_mdy), Required
	253	**friendly_time4**<br><br>Show the field ONLY if: [friendly_num] = '4' or [friendly_num] = '5'	Record date and the time of the fourth instance of friendliness.	text (datetime_mdy), Required
	254	**friendly_time5**<br><br>Show the field ONLY if: [friendly_num] = '5'	Record the date and time of the fifth instance of friendliness.	text (datetime_mdy), Required
	255	**relaxed_time_yn**<br><br>Show the field ONLY if: [postbup_intox(10)]='1'	Did the provider document the time(s) at which being relaxed was noted?	yesno, Required<br>1 Yes<br>0 No
	256	**relaxed_num**<br><br>Show the field ONLY if: [relaxed_time_yn] = '1'	How many instances of being relaxed were documented with timestamps within 6 hours after first buprenorphine administration?	radio, Required<br>0 0<br>1 1<br>2 2<br>3 3<br>4 4<br>5 5

© 2021 Herring AA et al. *JAMA Network Open.*

	257	**relaxed_time1**<br><br>**Show the field ONLY if: [relaxed_num] = '1' or [relaxed_num] = '2' or [relaxed_num] = '3' or [relaxed_num] = '4' or [relaxed_num] = '5'**	**Record the date and time of the first instance of being relaxed.**	**text (datetime_mdy), Required**
	258	**relaxed_time2**<br><br>**Show the field ONLY if: [relaxed_num] = '2' or [relaxed_num] = '3' or [relaxed_num] = '4' or [relaxed_num] = '5'**	**Record the date and time of the second instance of being relaxed.**	**text (datetime_mdy), Required**
	259	**relaxed_time3**<br><br>**Show the field ONLY if: [relaxed_num] = '3' or [relaxed_num] = '4' or [relaxed_num] = '5'**	**Record the date and time of the third instance of being relaxed.**	**text (datetime_mdy), Required**
	260	**relaxed_time4**<br><br>**Show the field ONLY if: [relaxed_num] = '4' or [relaxed_num] = '5'**	**Record the date and time of the fourth instance of being relaxed.**	**text (datetime_mdy), Required**
	261	**relaxed_time5**<br><br>**Show the field ONLY if: [relaxed_num] = '5'**	**Record the date and time of the fifth instance of being relaxed.**	**text (datetime_mdy), Required**
	262	**concentrate_time_yn**<br><br>**Show the field ONLY if: [postbup_intox(11)]='1'**	**Did the provider document the time(s) at which diffculty concentrating was noted?**	**yesno, Required**<br>**1 Yes**<br>**0 No**
	263	**concentrate_num**<br><br>**Show the field ONLY if: [concentrate_time_yn] = '1'**	**How many instances of diffculty concentrating were documented with timestamps within 6 hours after first buprenorphine administration?**	**radio, Required**<br>**0 0**<br>**1 1**<br>**2 2**<br>**3 3**<br>**4 4**<br>**5 5**
	264	**concentrate_time1**<br><br>**Show the field ONLY if: [concentrate_num] = '1' or [concentrate_num] = '2' or [concentrate_num] = '3' or [concentrate_num] = '4' or [concentrate_num] = '5'**	**Record the date and time of the first instance of diffculty concentrating.**	**text (datetime_mdy), Required**

© 2021 Herring AA et al. *JAMA Network Open.*

	265	**concentrate_time2**<br><br>**Show the field ONLY if: [concentrate_num] = '2' or [concentrate_num] = '3' or [concentrate_num] = '4' or [concentrate_num] = '5'**	**Record the date and time of the second instance of diffculty concentrating.**	**text (datetime_mdy), Required**
	266	**concentrate_time3**<br><br>**Show the field ONLY if: [concentrate_num] = '3' or [concentrate_num] = '4' or [concentrate_num] = '5'**	**Record the date and time of the third instance of diffculty concentrating.**	**text (datetime_mdy), Required**
	267	**concentrate_time4**<br><br>**Show the field ONLY if: [concentrate_num] = '4' or [concentrate_num] = '5'**	**Record the date and time of the fourth instance of diffculty concentrating.**	**text (datetime_mdy), Required**
	268	**concentrate_time5**<br><br>**Show the field ONLY if: [concentrate_num] = '5'**	**Record the date and time of the fifth instance of diffculty concentrating.**	**text (datetime_mdy), Required**
	269	**dizzy_time_yn**<br><br>**Show the field ONLY if: [postbup_intox(12)]='1'**	**Did the provider document the time(s) at which lightheadedness or dizziness were noted?**	**yesno, Required**<br>1 Yes<br>0 No
	270	**dizzy_num**<br><br>**Show the field ONLY if: [dizzy_time_yn] = '1'**	**How many instances of lightheadedness or dizziness were documented with timestamps within 6 hours after first buprenorphine administration?**	**radio, Required**<br>0 0<br>1 1<br>2 2<br>3 3<br>4 4<br>5 5
	271	**dizzy_time1**<br><br>**Show the field ONLY if: [dizzy_num] = '1' or [dizzy_num] = '2' or [dizzy_num] = '3' or [dizzy_num] = '4' or [dizzy_num] = '5'**	**Record the date and time of the first instance of lightheadedness or dizziness.**	**text (datetime_mdy), Required**
	272	**dizzy_time2**<br><br>**Show the field ONLY if: [dizzy_num] = '2' or [dizzy_num] = '3' or [dizzy_num] = '4' or [dizzy_num] = '5'**	**Record the date and time of the second instance of lightheadedness or dizziness.**	**text (datetime_mdy), Required**
	273	**dizzy_time3**<br><br>**Show the field ONLY if: [dizzy_num] = '3' or [dizzy_num] = '4' or [dizzy_num] = '5'**	**Record the date and time of the third instance of lightheadedness or dizziness.**	**text (datetime_mdy), Required**

© 2021 Herring AA et al. *JAMA Network Open.*

	274	dizzy_time4<br><br>Show the field ONLY if: [dizzy_num] = '4' or [dizzy_num] = '5'	Record the date and time of the fourth instance of lightheadedness or dizziness.	text (datetime_mdy), Required
	275	dizzy_time5<br><br>Show the field ONLY if: [dizzy_num] = '5'	Record the date and time of the fifth instance of lightheadedness or dizziness.	text (datetime_mdy), Required
	276	confusion_time_yn<br><br>Show the field ONLY if: [postbup_intox(13)]='1'	Did the provider document the time(s) at which confusion was noted?	yesno, Required<br>1 Yes<br>0 No
	277	confusion_num<br><br>Show the field ONLY if: [confusion_time_yn] = '1'	How many instances of confusion were documented with timestamps within 6 hours after first buprenorphine administration?	radio, Required<br>0 0<br>1 1<br>2 2<br>3 3<br>4 4<br>5 5
	278	confusion_time1<br><br>Show the field ONLY if: [confusion_num] = '1' or [confusion_num] = '2' or [confusion_num] = '3' or [confusion_num] = '4' or [confusion_num] = '5'	Record the date and time of the first instance of confusion.	text (datetime_mdy), Required
	279	confusion_time2<br><br>Show the field ONLY if: [confusion_num] = '2' or [confusion_num] = '3' or [confusion_num] = '4' or [confusion_num] = '5'	Record the date and time of the second instance of confusion.	text (datetime_mdy), Required
	280	confusion_time3<br><br>Show the field ONLY if: [confusion_num] = '3' or [confusion_num] = '4' or [confusion_num] = '5'	Record the date and time of the third instance of confusion.	text (datetime_mdy), Required
	281	confusion_time4<br><br>Show the field ONLY if: [confusion_num] = '4' or [confusion_num] = '5'	Record the date and time of the fourth instance of confusion.	text (datetime_mdy), Required
	282	confusion_time5<br><br>Show the field ONLY if: [confusion_num] = '5'	Record the date and time of the fifth instance of confusion.	text (datetime_mdy), Required

© 2021 Herring AA et al. *JAMA Network Open.*

	283	**postbup_gi**<br><br>**Show the field ONLY if: [postbup_ss] = '1'**	**Section Header:** *Post-Bup GI Symptoms*<br><br>**Please review the entire clinical chart and note if any of the following gastrointestinal signs or symptoms were documented within 6 hours after first buprenorphine administration.**	**checkbox, Required**<br>1 postbup_gi_1 Constipation<br>2 postbup_gi_2 Turning of stomach/cramping/abdo pain<br>3 postbup_gi_3 Nausea<br>4 postbup_gi_4 Vomiting<br>5 postbup_gi_5 Diarrhea<br>0 postbup_gi_0 None of the above
	284	**constipation_time_yn**<br><br>**Show the field ONLY if: [postbup_gi(1)] = '1'**	**Did the provider document the time(s) at which constipation was noted?**	**yesno, Required**<br>1 Yes<br>0 No
	285	**constipation_num**<br><br>**Show the field ONLY if: [constipation_time_yn] = '1'**	**How many instances of constipation were documented with timestamps within 6 hours after first buprenorphine administration?**	**radio, Required**<br>0 0<br>1 1<br>2 2<br>3 3<br>4 4<br>5 5
	286	**constipation_time1**<br><br>**Show the field ONLY if: [constipation_num] = '1' or [constipation_num] = '2' or [constipation_num] = '3' or [constipation_num] = '4' or [constipation_num] = '5'**	**Record the date and time of the first instance of constipation.**	**text (datetime_mdy), Required**
	287	**constipation_time2**<br><br>**Show the field ONLY if: [constipation_num] = '2' or [constipation_num] = '3' or [constipation_num] = '4' or [constipation_num] = '5'**	**Record the date and time of the second instance of constipation.**	**text (datetime_mdy), Required**
	288	**constipation_time3**<br><br>**Show the field ONLY if: [constipation_num] = '3' or [constipation_num] = '4' or [constipation_num] = '5'**	**Record the date and time of the third instance of constipation.**	**text (datetime_mdy), Required**
	289	**constipation_time4**<br><br>**Show the field ONLY if: [constipation_num] = '4' or [constipation_num] = '5'**	**Record the date and time of the fourth instance of constipation.**	**text (datetime_mdy), Required**

© 2021 Herring AA et al. *JAMA Network Open.*

	290	**constipation_time5**<br><br>**Show the field ONLY if: [constipation_num] = '5'**	**Record the date and time of the fifth instance of constipation.**	**text (datetime_mdy), Required**
	291	**stomach_time_yn**<br><br>**Show the field ONLY if: [postbup_gi(2)] = '1'**	**Did the provider document the time(s) at which stomach turning (stomach cramps) was noted?**	**yesno, Required**<br>1 Yes<br>0 No
	292	**stomach_num**<br><br>**Show the field ONLY if: [stomach_time_yn] = '1'**	**How many instances of stomach turning were documented with timestamps within 6 hours after first buprenorphine administration?**	**radio, Required**<br>0 0<br>1 1<br>2 2<br>3 3<br>4 4<br>5 5
	293	**stomach_time1**<br><br>**Show the field ONLY if: [stomach_num] = '1' or [stomach_num] = '2' or [stomach_num] = '3' or [stomach_num] = '4' or [stomach_num] = '5'**	**Record the date and time of the first instance of stomach turning.**	**text (datetime_mdy), Required**
	294	**stomach_time2**<br><br>**Show the field ONLY if: [stomach_num] = '2' or [stomach_num] = '3' or [stomach_num] = '4' or [stomach_num] = '5'**	**Record the date and time of the second instance of stomach turning.**	**text (datetime_mdy), Required**
	295	**stomach_time3**<br><br>**Show the field ONLY if: [stomach_num] = '3' or [stomach_num] = '4' or [stomach_num] = '5'**	**Record the date and time of the third instance of stomach turning.**	**text (datetime_mdy), Required**
	296	**stomach_time4**<br><br>**Show the field ONLY if: [stomach_num] = '4' or [stomach_num] = '5'**	**Record the date and time of the fourth instance of stomach turning.**	**text (datetime_mdy), Required**
	297	**stomach_time5**<br><br>**Show the field ONLY if: [stomach_num] = '5'**	**Record the date and time of the fifth instance of stomach turning.**	**text (datetime_mdy), Required**
	298	**nausea_time_yn**<br><br>**Show the field ONLY if: [postbup_gi(3)] = '1'**	**Did the provider document the time(s) at which nausea was noted?**	**yesno, Required**<br>1 Yes<br>0 No

© 2021 Herring AA et al. *JAMA Network Open.*

	299	**nausea_num**<br><br>**Show the field ONLY if: [nausea_time_yn] = '1'**	**How many instances of nausea were documented with timestamps within 6 hours after first buprenorphine administration?**	**radio, Required**<br>0 0<br>1 1<br>2 2<br>3 3<br>4 4<br>5 5
	300	**nausea_time1**<br><br>**Show the field ONLY if: [nausea_num] = '1' or [nausea_num] = '2' or [nausea_num] = '3' or [nausea_num] = '4' or [nausea_num] = '5'**	**Record the date and time of the first instance of nausea.**	**text (datetime_mdy), Required**
	301	**nausea_time2**<br><br>**Show the field ONLY if: [nausea_num] = '2' or [nausea_num] = '3' or [nausea_num] = '4' or [nausea_num] = '5'**	**Record the date and time of the second instance of nausea.**	**text (datetime_mdy), Required**
	302	**nausea_time3**<br><br>**Show the field ONLY if: [nausea_num] = '3' or [nausea_num] = '4' or [nausea_num] = '5'**	**Record the date and time of the third instance of nausea.**	**text (datetime_mdy), Required**
	303	**nausea_time4**<br><br>**Show the field ONLY if: [nausea_num] = '4' or [nausea_num] = '5'**	**Record the date and time of the fourth instance of nausea.**	**text (datetime_mdy), Required**
	304	**nausea_time5**<br><br>**Show the field ONLY if: [nausea_num] = '5'**	**Record the date and time of the fifth instance of nausea.**	**text (datetime_mdy), Required**
	305	**vomit_time_yn**<br><br>**Show the field ONLY if: [postbup_gi(4)] = '1'**	**Did the provider document the time(s) at which vomiting was noted?**	**yesno, Required**<br>1 Yes<br>0 No
	306	**vomit_num**<br><br>**Show the field ONLY if: [vomit_time_yn] = '1'**	**How many instances of vomiting were documented with timestamps within 6 hours after first buprenorphine administration?**	**radio, Required**<br>0 0<br>1 1<br>2 2<br>3 3<br>4 4<br>5 5

	307	**vomit_time1**<br><br>**Show the field ONLY if: [vomit_num] = '1' or [vomit_num] = '2' or [vomit_num] = '3' or [vomit_num] = '4' or [vomit_num] = '5'**	**Record the date and time of the first instance of vomiting.**	**text (datetime_mdy), Required**
	308	**vomit_time2**<br><br>**Show the field ONLY if: [vomit_num] = '2' or [vomit_num] = '3' or [vomit_num] = '4' or [vomit_num] = '5'**	**Record the date and time of the second instance of vomiting.**	**text (datetime_mdy), Required**
	309	**vomit_time3**<br><br>**Show the field ONLY if: [vomit_num] = '3' or [vomit_num] = '4' or [vomit_num] = '5'**	**Record the date and time of the third instance of vomiting.**	**text (datetime_mdy), Required**
	310	**vomit_time4**<br><br>**Show the field ONLY if: [vomit_num] = '4' or [vomit_num] = '5'**	**Record the date and time of the fourth instance of vomiting.**	**text (datetime_mdy), Required**
	311	**vomit_time5**<br><br>**Show the field ONLY if: [vomit_num] = '5'**	**Record the date and time of the fifth instance of vomiting.**	**text (datetime_mdy), Required**
	312	**diarrhea_time_yn**<br><br>**Show the field ONLY if: [postbup_gi(5)] = '1'**	**Did the provider document the time(s) at which diarrhea was noted?**	**yesno, Required**<br>**1 Yes**<br>**0 No**
	313	**diarrhea_num**<br><br>**Show the field ONLY if: [diarrhea_time_yn] = '1'**	**How many instances of diarrhea were documented with timestamps within 6 hours after first buprenorphine administration?**	**radio, Required**<br>**0 0**<br>**1 1**<br>**2 2**<br>**3 3**<br>**4 4**<br>**5 5**
	314	**diarrhea_time1**<br><br>**Show the field ONLY if: [diarrhea_num] = '1' or [diarrhea_num] = '2' or [diarrhea_num] = '3' or [diarrhea_num] = '4' or [diarrhea_num] = '5'**	**Record the date and time of the first instance of diarrhea.**	**text (datetime_mdy), Required**
	315	**diarrhea_time2**<br><br>**Show the field ONLY if: [diarrhea_num] = '2' or [diarrhea_num] = '3' or [diarrhea_num] = '4' or [diarrhea_num] = '5'**	**Record the date and time of the second instance of diarrhea.**	**text (datetime_mdy), Required**

© 2021 Herring AA et al. *JAMA Network Open.*

<table>
<tr><td></td><td>316</td><td>diarrhea_time3

Show the field ONLY if: [diarrhea_num] = '3' or [diarrhea_num] = '4' or [diarrhea_num] = '5'</td><td>Record the date and time of the third instance of diarrhea.</td><td>text (datetime_mdy), Required</td></tr>
<tr><td></td><td>317</td><td>diarrhea_time4

Show the field ONLY if: [diarrhea_num] = '4' or [diarrhea_num] = '5'</td><td>Record the date and time of the fourth instance of diarrhea.</td><td>text (datetime_mdy), Required</td></tr>
<tr><td></td><td>318</td><td>diarrhea_time5

Show the field ONLY if: [diarrhea_num] = '5'</td><td>Record the date and time of the fifth instance of diarrhea.</td><td>text (datetime_mdy), Required</td></tr>
<tr><td></td><td>319</td><td>postbup_anxious

Show the field ONLY if: [postbup_ss] = '1'</td><td>Section Header: Post-Bup Anxiety

Please review the entire clinical chart and note if any of the following signs or symptoms of anxiety were documented within 6 hours after first buprenorphine administration.</td><td>checkbox, Required<table>
<tr><td>1</td><td>postbup_anxious_1</td><td>Anxiety, irritability, or agitation</td></tr>
<tr><td>2</td><td>postbup_anxious_2</td><td>Nervousness</td></tr>
<tr><td>3</td><td>postbup_anxious_3</td><td>Tremor</td></tr>
<tr><td>4</td><td>postbup_anxious_4</td><td>Restlessness</td></tr>
<tr><td>0</td><td>postbup_anxious_0</td><td>None of the above</td></tr>
</table></td></tr>
<tr><td></td><td>320</td><td>anxiety_time_yn

Show the field ONLY if: [postbup_anxious(1)] = '1'</td><td>Did the provider document the time(s) at which anxiety, irritability, or agitation were noted?</td><td>yesno, Required<table>
<tr><td>1</td><td>Yes</td></tr>
<tr><td>0</td><td>No</td></tr>
</table></td></tr>
<tr><td></td><td>321</td><td>anxiety_num

Show the field ONLY if: [anxiety_time_yn] = '1'</td><td>How many instances of anxiety, irritability, or agitation were documented with timestamps within 6 hours after first buprenorphine administration?</td><td>radio, Required<table>
<tr><td>0</td><td>0</td></tr>
<tr><td>1</td><td>1</td></tr>
<tr><td>2</td><td>2</td></tr>
<tr><td>3</td><td>3</td></tr>
<tr><td>4</td><td>4</td></tr>
<tr><td>5</td><td>5</td></tr>
</table></td></tr>
<tr><td></td><td>322</td><td>anxiety_time1

Show the field ONLY if: [anxiety_num] = '1' or [anxiety_num] = '2' or [anxiety_num] = '3' or [anxiety_num] = '4' or [anxiety_num] = '5'</td><td>Record the date and time of the first instance of anxiety, irritability, or agitation.</td><td>text (datetime_mdy), Required</td></tr>
<tr><td></td><td>323</td><td>anxiety_time2

Show the field ONLY if: [anxiety_num] = '2' or [anxiety_num] = '3' or [anxiety_num] = '4' or [anxiety_num] = '5'</td><td>Record the date and time of the second instance of anxiety, irritability, or agitation.</td><td>text (datetime_mdy), Required</td></tr>
</table>

© 2021 Herring AA et al. *JAMA Network Open.*

	324	**anxiety_time3**<br>**Show the field ONLY if: [anxiety_num] = '3' or [anxiety_num] = '4' or [anxiety_num] = '5'**	**Record the date and time of the third instance of anxiety, irritability, or agitation.**	**text (datetime_mdy), Required**
	325	**anxiety_time4**<br>**Show the field ONLY if: [anxiety_num] = '4' or [anxiety_num] = '5'**	**Record the date and time of the fourth instance of anxiety, irritability, or agitation.**	**text (datetime_mdy), Required**
	326	**anxiety_time5**<br>**Show the field ONLY if: [anxiety_num] = '5'**	**Record the date and time of the fifth instance of anxiety, irritability, or agitation.**	**text (datetime_mdy), Required**
	327	**nervous_time_yn**<br>**Show the field ONLY if: [postbup_anxious(2)] = '1'**	**Did the provider document the time(s) at which nervousness was noted?**	**yesno, Required**<br>1 Yes<br>0 No
	328	**nervous_num**<br>**Show the field ONLY if: [nervous_time_yn] = '1'**	**How many instances of nervousness were documented with timestamps within 6 hours after first buprenorphine administration?**	**radio, Required**<br>0 0<br>1 1<br>2 2<br>3 3<br>4 4<br>5 5
	329	**nervous_time1**<br>**Show the field ONLY if: [nervous_num] = '1' or [nervous_num] = '2' or [nervous_num] = '3' or [nervous_num] = '4' or [nervous_num] = '5'**	**Record the date and time of the first instance of nervousness.**	**text (datetime_mdy), Required**
	330	**nervous_time2**<br>**Show the field ONLY if: [nervous_num] = '2' or [nervous_num] = '3' or [nervous_num] = '4' or [nervous_num] = '5'**	**Record the date and time of the second instance of nervousness.**	**text (datetime_mdy), Required**
	331	**nervous_time3**<br>**Show the field ONLY if: [nervous_num] = '3' or [nervous_num] = '4' or [nervous_num] = '5'**	**Record the date and time of the third instance of nervousness.**	**text (datetime_mdy), Required**
	332	**nervous_time4**<br>**Show the field ONLY if: [nervous_num] = '4' or [nervous_num] = '5'**	**Record the date and time of the fourth instance of nervousness.**	**text (datetime_mdy), Required**

© 2021 Herring AA et al. *JAMA Network Open.*

	333	**nervous_time5**<br><br>**Show the field ONLY if: [nervous_num] = '5'**	**Record the date and time of the fifth instance of nervousness.**	**text (datetime_mdy), Required**
	334	**tremor_time_yn**<br><br>**Show the field ONLY if: [postbup_anxious(3)] = '1'**	**Did the provider document the time(s) at which tremor was noted?**	**yesno, Required**<br>1 Yes<br>0 No
	335	**tremor_num**<br><br>**Show the field ONLY if: [tremor_time_yn] = '1'**	**How many instances of tremor were documented with timestamps within 6 hours after first buprenorphine administration?**	**radio, Required**<br>0 0<br>1 1<br>2 2<br>3 3<br>4 4<br>5 5
	336	**tremor_time1**<br><br>**Show the field ONLY if: [tremor_num] = '1' or [tremor_num] = '2' or [tremor_num] = '3' or [tremor_num] = '4' or [tremor_num] = '5'**	**Record the date and time of the first instance of tremor.**<br>*Enter military time (xx:xx).*	**text (datetime_mdy), Required**
	337	**tremor_time2**<br><br>**Show the field ONLY if: [tremor_num] = '2' or [tremor_num] = '3' or [tremor_num] = '4' or [tremor_num] = '5'**	**Record the date and time of the second instance of tremor.**	**text (datetime_mdy), Required**
	338	**tremor_time3**<br><br>**Show the field ONLY if: [tremor_num] = '3' or [tremor_num] = '4' or [tremor_num] = '5'**	**Record the date and time of the third instance of tremor.**	**text (datetime_mdy), Required**
	339	**tremor_time4**<br><br>**Show the field ONLY if: [tremor_num] = '4' or [tremor_num] = '5'**	**Record the date and time of the fourth instance of tremor.**	**text (datetime_mdy), Required**
	340	**tremor_time5**<br><br>**Show the field ONLY if: [tremor_num] = '5'**	**Record the date and time of the fifth instance of tremor.**	**text (datetime_mdy), Required**
	341	**restless_time_yn**<br><br>**Show the field ONLY if: [postbup_anxious(4)] = '1'**	**Did the provider document the time(s) at which restlessness was noted?**	**yesno, Required**<br>1 Yes<br>0 No

© 2021 Herring AA et al. *JAMA Network Open.*

	342	**restless_num**<br><br>**Show the field ONLY if: [restless_time_yn] = '1'**	**How many instances of restlessness were documented with timestamps within 6 hours after first buprenorphine administration?**	**radio, Required**<br>0 0<br>1 1<br>2 2<br>3 3<br>4 4<br>5 5
	343	**restless_time1**<br><br>**Show the field ONLY if: [restless_num] = '1' or [restless_num] = '2' or [restless_num] = '3' or [restless_num] = '4' or [restless_num] = '5'**	**Record the date and time of the first instance of restlessness.**	**text (datetime_mdy), Required**
	344	**restless_time2**<br><br>**Show the field ONLY if: [restless_num] = '2' or [restless_num] = '3' or [restless_num] = '4' or [restless_num] = '5'**	**Record the date and time of the second instance of restlessness.**	**text (datetime_mdy), Required**
	345	**restless_time3**<br><br>**Show the field ONLY if: [restless_num] = '3' or [restless_num] = '4' or [restless_num] = '5'**	**Record the date and time of the third instance of restlessness.**	**text (datetime_mdy), Required**
	346	**restless_time4**<br><br>**Show the field ONLY if: [restless_num] = '4' or [restless_num] = '5'**	**Record the date and time of the fourth instance of restlessness.**	**text (datetime_mdy), Required**
	347	**restless_time5**<br><br>**Show the field ONLY if: [restless_num] = '5'**	**Record the date and time of the fifth instance of restlessness.**	**text (datetime_mdy), Required**

© 2021 Herring AA et al. *JAMA Network Open.*

	348	**postbup_withdrawal**<br><br>**Show the field ONLY if:**<br>**[postbup_ss] = '1'**	**Section Header:** *Post-Bup Withdrawal*<br><br>**Please review the entire clinical chart and note if any of the following signs or symptoms of withdrawal were documented within 6 hours after first buprenorphine administration.**	**checkbox, Required**<br>1 | postbup_withdrawal_1 | Yawning<br>2 | postbup_withdrawal_2 | Gooseflesh skin (chills/piloerecti<br>3 | postbup_withdrawal_3 | Pulse > 80<br>4 | postbup_withdrawal_4 | Sweating (diaphoresis)<br>5 | postbup_withdrawal_5 | Pupil size larger normal (dilation/mydria<br>6 | postbup_withdrawal_6 | Bone, joint ache body aches (arthralgias/mva<br>7 | postbup_withdrawal_7 | Runny nose or tearing (rhinorre lacrimation)<br>0 | postbup_withdrawal_0 | None of the abo
	349	**yawning_time_yn**<br><br>**Show the field ONLY if:**<br>**[postbup_withdrawal(1)] = '1'**	**Did the provider document the time(s) at which yawning was noted?**	**yesno, Required**<br>1 | Yes<br>0 | No
	350	**yawning_num**<br><br>**Show the field ONLY if:**<br>**[yawning_time_yn] = '1'**	**How many instances of yawning were documented with timestamps within 6 hours after first buprenorphine administration?**	**radio, Required**<br>0 | 0<br>1 | 1<br>2 | 2<br>3 | 3<br>4 | 4<br>5 | 5
	351	**yawning_time1**<br><br>**Show the field ONLY if:**<br>**[yawning_num] = '1' or [yawning_num] = '2' or [yawning_num] = '3' or [yawning_num] = '4' or [yawning_num] = '5'**	**Record the date and time of the first instance of yawning.**	**text (datetime_mdy), Required**
	352	**yawning_time2**<br><br>**Show the field ONLY if:**<br>**[yawning_num] = '2' or [yawning_num] = '3' or [yawning_num] = '4' or [yawning_num] = '5'**	**Record the date and time of the second instance of yawning.**	**text (datetime_mdy), Required**
	353	**yawning_time3**<br><br>**Show the field ONLY if:**<br>**[yawning_num] = '3' or [yawning_num] = '4' or [yawning_num] = '5'**	**Record the date and time of the third instance of yawning.**	**text (datetime_mdy), Required**

© 2021 Herring AA et al. *JAMA Network Open.*

	354	yawning_time4<br><br>Show the field ONLY if: [yawning_num] = '4' or [yawning_num] = '5'	Record the date and time of the fourth instance of yawning.	text (datetime_mdy), Required
	355	yawning_time5<br><br>Show the field ONLY if: [yawning_num] = '5'	Record the date and time of the fifth instance of yawning.	text (datetime_mdy), Required
	356	chills_time_yn<br><br>Show the field ONLY if: [postbup_withdrawal(2)] = '1'	Did the provider document the time(s) at which gooseflesh skin (chills/piloerection) was noted?	yesno, Required<br>1 Yes<br>0 No
	357	chills_num<br><br>Show the field ONLY if: [chills_time_yn] = '1'	How many instances of chills were documented with timestamps within 6 hours after first buprenorphine administration?	radio, Required<br>0 0<br>1 1<br>2 2<br>3 3<br>4 4<br>5 5
	358	chills_time1<br><br>Show the field ONLY if: [chills_num] = '1' or [chills_num] = '2' or [chills_num] = '3' or [chills_num] = '4' or [chills_num] = '5'	Record the date and time of the first instance of chills.	text (datetime_mdy), Required
	359	chills_time2<br><br>Show the field ONLY if: [chills_num] = '2' or [chills_num] = '3' or [chills_num] = '4' or [chills_num] = '5'	Record the date and time of the second instance of chills.	text (datetime_mdy), Required
	360	chills_time3<br><br>Show the field ONLY if: [chills_num] = '3' or [chills_num] = '4' or [chills_num] = '5'	Record the date and time of the third instance of chills.	text (datetime_mdy), Required
	361	chills_time4<br><br>Show the field ONLY if: [chills_num] = '4' or [chills_num] = '5'	Record the date and time of the fourth instance of chills.	text (datetime_mdy), Required
	362	chills_time5<br><br>Show the field ONLY if: [chills_num] = '5'	Record the date and time of the fifth instance of chills.	text (datetime_mdy), Required
	363	tachy_time_yn<br><br>Show the field ONLY if: [postbup_withdrawal(3)] = '1'	Did the provider document the time(s) at which a pulse > 80 bpm was noted?	yesno, Required<br>1 Yes<br>0 No

© 2021 Herring AA et al. *JAMA Network Open.*

	364	**tachy_num**<br><br>**Show the field ONLY if: [tachy_time_yn] = '1'**	**How many instances of a pulse > 80 bpm were documented with timestamps within 6 hours after first buprenorphine administration?**	**radio, Required**<br>0 0<br>1 1<br>2 2<br>3 3<br>4 4<br>5 5
	365	**tachy_time1**<br><br>**Show the field ONLY if: [tachy_num] = '1' or [tachy_num] = '2' or [tachy_num] = '3' or [tachy_num] = '4' or [tachy_num] = '5'**	**Record the date and time of the first instance of pulse > 80 bpm.**	**text (datetime_mdy), Required**
	366	**tachy_time2**<br><br>**Show the field ONLY if: [tachy_num] = '2' or [tachy_num] = '3' or [tachy_num] = '4' or [tachy_num] = '5'**	**Record the date and time of the second instance of pulse > 80 bpm.**	**text (datetime_mdy), Required**
	367	**tachy_time3**<br><br>**Show the field ONLY if: [tachy_num] = '3' or [tachy_num] = '4' or [tachy_num] = '5'**	**Record the date and time of the third instance of pulse > 80 bpm.**	**text (datetime_mdy), Required**
	368	**tachy_time4**<br><br>**Show the field ONLY if: [tachy_num] = '4' or [tachy_num] = '5'**	**Record the date and time of the fourth instance of pulse > 80 bpm.**	**text (datetime_mdy), Required**
	369	**tachy_time5**<br><br>**Show the field ONLY if: [tachy_num] = '5'**	**Record the date and time of the fifth instance of pulse > 80 bpm.**	**text (datetime_mdy), Required**
	370	**sweat_time_yn**<br><br>**Show the field ONLY if: [postbup_withdrawal(4)] = '1'**	**Did the provider document the time(s) at which sweating was noted?**	**yesno, Required**<br>1 Yes<br>0 No
	371	**sweat_num**<br><br>**Show the field ONLY if: [sweat_time_yn] = '1'**	**How many instances of sweating were documented with timestamps within 6 hours after first buprenorphine administration?**	**radio, Required**<br>0 0<br>1 1<br>2 2<br>3 3<br>4 4<br>5 5

	372	**sweat_time1**<br><br>**Show the field ONLY if: [sweat_num] = '1' or [sweat_num] = '2' or [sweat_num] = '3' or [sweat_num] = '4' or [sweat_num] = '5'**	**Record the date and time of the first instance of sweating.**	**text (datetime_mdy), Required**
	373	**sweat_time2**<br><br>**Show the field ONLY if: [sweat_num] = '2' or [sweat_num] = '3' or [sweat_num] = '4' or [sweat_num] = '5'**	**Record the date and time of the second instance of sweating.**	**text (datetime_mdy), Required**
	374	**sweat_time3**<br><br>**Show the field ONLY if: [sweat_num] = '3' or [sweat_num] = '4' or [sweat_num] = '5'**	**Record the date and time of the third instance of sweating.**	**text (datetime_mdy), Required**
	375	**sweat_time4**<br><br>**Show the field ONLY if: [sweat_num] = '4' or [sweat_num] = '5'**	**Record the date and time of the fourth instance of sweating.**	**text (datetime_mdy), Required**
	376	**sweat_time5**<br><br>**Show the field ONLY if: [sweat_num] = '5'**	**Record the date and time of the fifth instance of sweating.**	**text (datetime_mdy), Required**
	377	**dilation_time_yn**<br><br>**Show the field ONLY if: [postbup_withdrawal(5)] = '1'**	**Did the provider document the time(s) at which pupil dilation (mydriasis) was noted?**	**yesno, Required**<br>**1 Yes**<br>**0 No**
	378	**dilation_num**<br><br>**Show the field ONLY if: [dilation_time_yn] = '1'**	**How many instances of pupil dilation were documented with timestamps within 6 hours after first buprenorphine administration?**	**radio, Required**<br>**0 0**<br>**1 1**<br>**2 2**<br>**3 3**<br>**4 4**<br>**5 5**
	379	**dilation_time1**<br><br>**Show the field ONLY if: [dilation_num] = '1' or [dilation_num] = '2' or [dilation_num] = '3' or [dilation_num] = '4' or [dilation_num] = '5'**	**Record the date and time of the first instance of pupil dilation.**	**text (datetime_mdy), Required**

© 2021 Herring AA et al. *JAMA Network Open.*

	380	**postbup_dilate_mm_1**<br><br>**Show the field ONLY if: [dilation_num] = '1' or [dilation_num] = '2' or [dilation_num] = '3' or [dilation_num] = '4' or [dilation_num] = '5'**	**Pupil measurement listed?**	**yesno, Required**<br>1 Yes<br>0 No
	381	**postbup_dilate_size_1**<br><br>**Show the field ONLY if: [postbup_dilate_mm_1] = '1'**	**Please enter the pupil size measured (in mm)**	**text, Required**
	382	**dilation_time2**<br><br>**Show the field ONLY if: [dilation_num] = '2' or [dilation_num] = '3' or [dilation_num] = '4' or [dilation_num] = '5'**	**Record the date and time of the second instance of pupil dilation.**	**text (datetime_mdy), Required**
	383	**postbup_dilate_mm_2**<br><br>**Show the field ONLY if: [dilation_num] = '2' or [dilation_num] = '3' or [dilation_num] = '4' or [dilation_num] = '5'**	**Pupil measurement listed?**	**yesno, Required**<br>1 Yes<br>0 No
	384	**postbup_dilate_size_2**<br><br>**Show the field ONLY if: [postbup_dilate_mm_2] = '1'**	**Please enter the pupil size measured (in mm)**	**text, Required**
	385	**dilation_time3**<br><br>**Show the field ONLY if: [dilation_num] = '3' or [dilation_num] = '4' or [dilation_num] = '5'**	**Record the date and time of the third instance of pupil dilation.**	**text (datetime_mdy), Required**
	386	**postbup_dilate_mm_3**<br><br>**Show the field ONLY if: [dilation_num] = '3' or [dilation_num] = '4' or [dilation_num] = '5'**	**Pupil measurement listed?**	**yesno, Required**<br>1 Yes<br>0 No
	387	**postbup_dilate_size_3**<br><br>**Show the field ONLY if: [postbup_dilate_mm_3] = '1'**	**Please enter the pupil size measured (in mm)**	**text, Required**
	388	**dilation_time4**<br><br>**Show the field ONLY if: [dilation_num] = '4' or [dilation_num] = '5'**	**Record the date and time of the fourth instance of pupil dilation.**	**text (datetime_mdy), Required**
	389	**postbup_dilate_mm_4**<br><br>**Show the field ONLY if: [dilation_num] = '4' or [dilation_num] = '5'**	**Pupil measurement listed?**	**yesno, Required**<br>1 Yes<br>0 No

© 2021 Herring AA et al. *JAMA Network Open.*

	390	postbup_dilate_size_4<br><br>Show the field ONLY if: [postbup_dilate_mm_4] = '1'	Please enter the pupil size measured (in mm)	text, Required
	391	dilation_time5<br><br>Show the field ONLY if: [dilation_num] = '5'	Record the date and time of the fifth instance of pupil dilation.	text (datetime_mdy), Required
	392	postbup_dilate_mm_5<br><br>Show the field ONLY if: [dilation_num] = '5'	Pupil measurement listed?	yesno, Required<br>1 Yes<br>0 No
	393	postbup_dilate_size_5<br><br>Show the field ONLY if: [postbup_dilate_mm_5] = '1'	Please enter the pupil size measured (in mm)	text, Required
	394	aches_time_yn<br><br>Show the field ONLY if: [postbup_withdrawal(6)] = '1'	Did the provider document the time(s) at which bone, joint, or body aches were noted?	yesno, Required<br>1 Yes<br>0 No
	395	aches_num<br><br>Show the field ONLY if: [aches_time_yn] = '1'	How many instances of bone, joint, or body aches were documented with timestamps within 6 hours after first buprenorphine administration?	radio, Required<br>0 0<br>1 1<br>2 2<br>3 3<br>4 4<br>5 5
	396	aches_time1<br><br>Show the field ONLY if: [aches_num] = '1' or [aches_num] = '2' or [aches_num] = '3' or [aches_num] = '4' or [aches_num] = '5'	Record the date and time of the first instance of bone, joint, or body aches.	text (datetime_mdy), Required
	397	aches_time2<br><br>Show the field ONLY if: [aches_num] = '2' or [aches_num] = '3' or [aches_num] = '4' or [aches_num] = '5'	Record the date and time of the second instance of bone, joint, or body aches.	text (datetime_mdy), Required
	398	aches_time3<br><br>Show the field ONLY if: [aches_num] = '3' or [aches_num] = '4' or [aches_num] = '5'	Record the date and time of the third instance of bone, joint, or body aches.	text (datetime_mdy), Required
	399	aches_time4<br><br>Show the field ONLY if: [aches_num] = '4' or [aches_num] = '5'	Record the date and time of the fourth instance of bone, joint, or body aches.	text (datetime_mdy), Required

© 2021 Herring AA et al. *JAMA Network Open.*

	400	aches_time5<br><br>Show the field ONLY if: [aches_num] = '5'	Record the date and time of the fifth instance of bone, joint, or body aches.	text (datetime_mdy), Required
	401	teary_time_yn<br><br>Show the field ONLY if: [postbup_withdrawal(7)] = '1'	Did the provider document the time(s) at which a runny nose or tearing (rhinorrhea or lacrimation) were noted?	yesno, Required<br>1 Yes<br>0 No
	402	teary_num<br><br>Show the field ONLY if: [teary_time_yn] = '1'	How many instances of a runny nose or tearing were documented with timestamps within 6 hours after first buprenorphine administration?	radio, Required<br>0 0<br>1 1<br>2 2<br>3 3<br>4 4<br>5 5
	403	teary_time1<br><br>Show the field ONLY if: [teary_num] = '1' or [teary_num] = '2' or [teary_num] = '3' or [teary_num] = '4' or [teary_num] = '5'	Record the date and time of the first instance of a runny nose or tearing.	text (datetime_mdy), Required
	404	teary_time2<br><br>Show the field ONLY if: [teary_num] = '2' or [teary_num] = '3' or [teary_num] = '4' or [teary_num] = '5'	Record the date and time of the second instance of a runny nose or tearing.	text (datetime_mdy), Required
	405	teary_time3<br><br>Show the field ONLY if: [teary_num] = '3' or [teary_num] = '4' or [teary_num] = '5'	Record the date and time of the third instance of a runny nose or tearing.	text (datetime_mdy), Required
	406	teary_time4<br><br>Show the field ONLY if: [teary_num] = '4' or [teary_num] = '5'	Record the date and time of the fourth instance of a runny nose or tearing.	text (datetime_mdy), Required
	407	teary_time5<br><br>Show the field ONLY if: [teary_num] = '5'	Record the date and time of the fifth instance of a runny nose or tearing.	text (datetime_mdy), Required
	408	postbup_vitals<br><br>Show the field ONLY if: [postbup_ss] = '1'	Section Header: *Post-Bup Abnormal Vital Signs*<br><br>Please review the entire clinical chart and note if any of the following abnormal vital signs were documented within 6 hours after first buprenorphine administration.	checkbox, Required<br>1 postbup_vitals_1 Hypoxia<br>2 postbup_vitals_2 Hypercarbia<br>3 postbup_vitals_3 Hypotension<br>4 postbup_vitals_4 Bradycardia<br>0 postbup_vitals_0 None of the above

© 2021 Herring AA et al. *JAMA Network Open.*

	409	**hypoxia_time_yn**<br><br>**Show the field ONLY if: [postbup_vitals(1)] = '1'**	**Did the provider document the time(s) at which hypoxia was noted?**	**yesno, Required**<br>1 Yes<br>0 No
	410	**hypoxia_num**<br><br>**Show the field ONLY if: [hypoxia_time_yn] = '1'**	**How many instances of hypoxia were documented with timestamps within 6 hours after first buprenorphine administration?**	**radio, Required**<br>0 0<br>1 1<br>2 2<br>3 3<br>4 4<br>5 5
	411	**hypoxia_time1**<br><br>**Show the field ONLY if: [hypoxia_num] = '1' or [hypoxia_num] = '2' or [hypoxia_num] = '3' or [hypoxia_num] = '4' or [hypoxia_num] = '5'**	**Record the date and time of the first instance of hypoxia.**	**text (datetime_mdy), Required**
	412	**hypoxia_time2**<br><br>**Show the field ONLY if: [hypoxia_num] = '2' or [hypoxia_num] = '3' or [hypoxia_num] = '4' or [hypoxia_num] = '5'**	**Record the date and time of the second instance of hypoxia.**	**text (datetime_mdy), Required**
	413	**hypoxia_time3**<br><br>**Show the field ONLY if: [hypoxia_num] = '3' or [hypoxia_num] = '4' or [hypoxia_num] = '5'**	**Record the date and time of the third instance of hypoxia.**	**text (datetime_mdy), Required**
	414	**hypoxia_time4**<br><br>**Show the field ONLY if: [hypoxia_num] = '4' or [hypoxia_num] = '5'**	**Record the date and time of the fourth instance of hypoxia.**	**text (datetime_mdy), Required**
	415	**hypoxia_time5**<br><br>**Show the field ONLY if: [hypoxia_num] = '5'**	**Record the date and time of the fifth instance of hypoxia.**	**text (datetime_mdy), Required**
	416	**co2_time_yn**<br><br>**Show the field ONLY if: [postbup_vitals(2)] = '1'**	**Did the provider document the time(s) at which hypercarbia was noted?**	**yesno, Required**<br>1 Yes<br>0 No
	417	**co2_num**<br><br>**Show the field ONLY if: [co2_time_yn] = '1'**	**How many instances of hypercarbia were documented with timestamps within 6 hours after first buprenorphine administration?**	**radio, Required**<br>0 0<br>1 1<br>2 2<br>3 3<br>4 4<br>5 5

© 2021 Herring AA et al. *JAMA Network Open.*

	418	**co2_time1**<br>**Show the field ONLY if: [co2_num] = '1' or [co2_num] = '2' or [co2_num] = '3' or [co2_num] = '4' or [co2_num] = '5'**	**Record the date and time of the first instance of hypercarbia.**	**text (datetime_mdy), Required**
	419	**co2_time2**<br>**Show the field ONLY if: [co2_num] = '2' or [co2_num] = '3' or [co2_num] = '4' or [co2_num] = '5'**	**Record the date and time of the second instance of hypercarbia.**	**text (datetime_mdy), Required**
	420	**co2_time3**<br>**Show the field ONLY if: [co2_num] = '3' or [co2_num] = '4' or [co2_num] = '5'**	**Record the date and time of the third instance of hypercarbia.**	**text (datetime_mdy), Required**
	421	**co2_time4**<br>**Show the field ONLY if: [co2_num] = '4' or [co2_num] = '5'**	**Record the date and time of the fourth instance of hypercarbia.**	**text (datetime_mdy), Required**
	422	**co2_time5**<br>**Show the field ONLY if: [co2_num] = '5'**	**Record the date and time of the fifth instance of hypercarbia.**	**text (datetime_mdy), Required**
	423	**hypo_time_yn**<br>**Show the field ONLY if: [postbup_vitals(3)] = '1'**	**Did the provider document the time(s) at which hypotension was noted?**	**yesno, Required**<br>**1 Yes**<br>**0 No**
	424	**hypo_num**<br>**Show the field ONLY if: [hypo_time_yn] = '1'**	**How many instances of hypotension were documented with timestamps within 6 hours after first buprenorphine administration?**	**radio, Required**<br>**0 0**<br>**1 1**<br>**2 2**<br>**3 3**<br>**4 4**<br>**5 5**
	425	**hypo_time1**<br>**Show the field ONLY if: [hypo_num] = '1' or [hypo_num] = '2' or [hypo_num] = '3' or [hypo_num] = '4' or [hypo_num] = '5'**	**Record the date and time of the first instance of hypotension.**	**text (datetime_mdy), Required**
	426	**hypo_time2**<br>**Show the field ONLY if: [hypo_num] = '2' or [hypo_num] = '3' or [hypo_num] = '4' or [hypo_num] = '5'**	**Record the date and time of the second instance of hypotension.**	**text (datetime_mdy), Required**

© 2021 Herring AA et al. *JAMA Network Open.*

	427	**hypo_time3**<br>**Show the field ONLY if: [hypo_num] = '3' or [hypo_num] = '4' or [hypo_num] = '5'**	**Record the date and time of the third instance of hypotension.**	**text (datetime_mdy), Required**
	428	**hypo_time4**<br>**Show the field ONLY if: [hypo_num] = '4' or [hypo_num] = '5'**	**Record the date and time of the fourth instance of hypotension.**	**text (datetime_mdy), Required**
	429	**hypo_time5**<br>**Show the field ONLY if: [hypo_num] = '5'**	**Record the date and time of the fifth instance of hypotension.**	**text (datetime_mdy), Required**
	430	**brady_time_yn**<br>**Show the field ONLY if: [postbup_vitals(4)] = '1'**	**Did the provider document the time(s) at which bradycardia was noted?**	**yesno, Required**<br>1 Yes<br>0 No
	431	**brady_num**<br>**Show the field ONLY if: [brady_time_yn] = '1'**	**How many instances of bradycardia were documented with timestamps within 6 hours after first buprenorphine administration?**	**radio, Required**<br>0 0<br>1 1<br>2 2<br>3 3<br>4 4<br>5 5
	432	**brady_time1**<br>**Show the field ONLY if: [brady_num] = '1' or [brady_num] = '2' or [brady_num] = '3' or [brady_num] = '4' or [brady_num] = '5'**	**Record the date and time of the first instance of bradycardia.**	**text (datetime_mdy), Required**
	433	**brady_time2**<br>**Show the field ONLY if: [brady_num] = '2' or [brady_num] = '3' or [brady_num] = '4' or [brady_num] = '5'**	**Record the date and time of the second instance of bradycardia.**	**text (datetime_mdy), Required**
	434	**brady_time3**<br>**Show the field ONLY if: [brady_num] = '3' or [brady_num] = '4' or [brady_num] = '5'**	**Record the date and time of the third instance of bradycardia.**	**text (datetime_mdy), Required**
	435	**brady_time4**<br>**Show the field ONLY if: [brady_num] = '4' or [brady_num] = '5'**	**Record the date and time of the fourth instance of bradycardia.**	**text (datetime_mdy), Required**
	436	**brady_time5**<br>**Show the field ONLY if: [brady_num] = '5'**	**Record the date and time of the fifth instance of bradycardia.**	**text (datetime_mdy), Required**

© 2021 Herring AA et al. *JAMA Network Open.*

	437	**postbup_misc_sx**<br>**Show the field ONLY if: [postbup_ss] ='1'**	**Section Header:** *Post-Bup Miscellaneous Opioid Related Side Effects*<br>**Please review the entire clinical chart and note if any of the following miscellaneous opioid related side effects were documented within 6 hours after first buprenorphine administration.**	**checkbox, Required**<br>1 postbup_misc_sx_1 Diffculty passing urine<br>2 postbup_misc_sx_2 Itchiness<br>3 postbup_misc_sx_3 Dry mouth<br>4 postbup_misc_sx_4 Headache<br>5 postbup_misc_sx_5 Unable to walk<br>6 postbup_misc_sx_6 Unable to discharge<br>7 postbup_misc_sx_7 Pupil size smaller than normal (constriction/miosis<br>8 postbup_misc_sx_8 Pupil size return to normal<br>0 postbup_misc_sx_0 None of the above
	438	**urine_time_yn**<br>**Show the field ONLY if: [postbup_misc_sx(1)] = '1'**	**Did the provider document the time(s) at which diffculty urinating was noted?**	**yesno, Required**<br>1 Yes<br>0 No
	439	**urine_num**<br>**Show the field ONLY if: [urine_time_yn] = '1'**	**How many instances of diffculty urinating were documented with timestamps within 6 hours after first buprenorphine administration?**	**radio, Required**<br>0 0<br>1 1<br>2 2<br>3 3<br>4 4<br>5 5
	440	**urine_time1**<br>**Show the field ONLY if: [urine_num] = '1' or [urine_num] = '2' or [urine_num] = '3' or [urine_num] = '4' or [urine_num] = '5'**	**Record the date and time of the first instance of diffculty urinating.**	**text (datetime_mdy), Required**
	441	**urine_time2**<br>**Show the field ONLY if: [urine_num] = '2' or [urine_num] = '3' or [urine_num] = '4' or [urine_num] = '5'**	**Record the date and time of the second instance of diffculty urinating.**	**text (datetime_mdy), Required**
	442	**urine_time3**<br>**Show the field ONLY if: [urine_num] = '3' or [urine_num] = '4' or [urine_num] = '5'**	**Record the date and time of the third instance of diffculty urinating.**	**text (datetime_mdy), Required**
	443	**urine_time4**<br>**Show the field ONLY if: [urine_num] = '4' or [urine_num] = '5'**	**Record the date and time of the fourth instance of diffculty urinating.**	**text (datetime_mdy), Required**

© 2021 Herring AA et al. *JAMA Network Open.*

	444	**urine_time5**<br><br>**Show the field ONLY if: [urine_num] = '5'**	**Record the date and time of the fifth instance of diffculty urinating.**	**text (datetime_mdy), Required**
	445	**itchy_time_yn**<br><br>**Show the field ONLY if: [postbup_misc_sx(2)] = '1'**	**Did the provider document the time(s) at which itchiness was noted?**	**yesno, Required**<br>1 Yes<br>0 No
	446	**itchy_num**<br><br>**Show the field ONLY if: [itchy_time_yn] = '1'**	**How many instances of itchiness were documented with timestamps within 6 hours after first buprenorphine administration?**	**radio, Required**<br>0 0<br>1 1<br>2 2<br>3 3<br>4 4<br>5 5
	447	**itchy_time1**<br><br>**Show the field ONLY if: [itchy_num] = '1' or [itchy_num] = '2' or [itchy_num] = '3' or [itchy_num] = '4' or [itchy_num] = '5'**	**Record the date and time of the first instance of itchiness.**	**text (datetime_mdy), Required**
	448	**itchy_time2**<br><br>**Show the field ONLY if: [itchy_num] = '2' or [itchy_num] = '3' or [itchy_num] = '4' or [itchy_num] = '5'**	**Record the date and time of the second instance of itchiness.**	**text (datetime_mdy), Required**
	449	**itchy_time3**<br><br>**Show the field ONLY if: [itchy_num] = '3' or [itchy_num] = '4' or [itchy_num] = '5'**	**Record the date and time of the third instance of itchiness.**	**text (datetime_mdy), Required**
	450	**itchy_time4**<br><br>**Show the field ONLY if: [itchy_num] = '4' or [itchy_num] = '5'**	**Record the date and time of the fourth instance of itchiness.**	**text (datetime_mdy), Required**
	451	**itchy_time5**<br><br>**Show the field ONLY if: [itchy_num] = '5'**	**Record the date and time of the fifth instance of itchiness.**	**text (datetime_mdy), Required**
	452	**mouth_time_yn**<br><br>**Show the field ONLY if: [postbup_misc_sx(3)] = '1'**	**Did the provider document the time(s) at which dry mouth was noted?**	**yesno, Required**<br>1 Yes<br>0 No

© 2021 Herring AA et al. *JAMA Network Open.*

	453	**mouth_num**<br><br>**Show the field ONLY if: [mouth_time_yn] = '1'**	**How many instances of dry mouth were documented with timestamps within 6 hours after first buprenorphine administration?**	**radio, Required**<br>0 0<br>1 1<br>2 2<br>3 3<br>4 4<br>5 5
	454	**mouth_time1**<br><br>**Show the field ONLY if: [mouth_num] = '1' or [mouth_num] = '2' or [mouth_num] = '3' or [mouth_num] = '4' or [mouth_num] = '5'**	**Record the date and time of the first instance of dry mouth.**	**text (datetime_mdy), Required**
	455	**mouth_time2**<br><br>**Show the field ONLY if: [mouth_num] = '2' or [mouth_num] = '3' or [mouth_num] = '4' or [mouth_num] = '5'**	**Record the date and time of the second instance of dry mouth.**	**text (datetime_mdy), Required**
	456	**mouth_time3**<br><br>**Show the field ONLY if: [mouth_num] = '3' or [mouth_num] = '4' or [mouth_num] = '5'**	**Record the date and time of the third instance of dry mouth.**	**text (datetime_mdy), Required**
	457	**mouth_time4**<br><br>**Show the field ONLY if: [mouth_num] = '4' or [mouth_num] = '5'**	**Record the date and time of the fourth instance of dry mouth.**	**text (datetime_mdy), Required**
	458	**mouth_time5**<br><br>**Show the field ONLY if: [mouth_num] = '5'**	**Record the date and time of the fifth instance of dry mouth.**	**text (datetime_mdy), Required**
	459	**headache_time_yn**<br><br>**Show the field ONLY if: [postbup_misc_sx(4)] = '1'**	**Did the provider document the time(s) at which headache was noted?**	**yesno, Required**<br>1 Yes<br>0 No
	460	**headache_num**<br><br>**Show the field ONLY if: [headache_time_yn] = '1'**	**How many instances of headache were documented with timestamps within 6 hours after first buprenorphine administration?**	**radio, Required**<br>0 0<br>1 1<br>2 2<br>3 3<br>4 4<br>5 5

© 2021 Herring AA et al. *JAMA Network Open.*

	461	**headache_time1**<br>**Show the field ONLY if: [headache_num] = '1' or [headache_num] = '2' or [headache_num] = '3' or [headache_num] = '4' or [headache_num] = '5'**	**Record the date and time of the first instance of headache.**	**text (datetime_mdy), Required**
	462	**headache_time2**<br>**Show the field ONLY if: [headache_num] = '2' or [headache_num] = '3' or [headache_num] = '4' or [headache_num] = '5'**	**Record the date and time of the second instance of headache.**	**text (datetime_mdy), Required**
	463	**headache_time3**<br>**Show the field ONLY if: [headache_num] = '3' or [headache_num] = '4' or [headache_num] = '5'**	**Record the date and time of the third instance of headache.**	**text (datetime_mdy), Required**
	464	**headache_time4**<br>**Show the field ONLY if: [headache_num] = '4' or [headache_num] = '5'**	**Record the date and time of the fourth instance of headache.**	**text (datetime_mdy), Required**
	465	**headache_time5**<br>**Show the field ONLY if: [headache_num] = '5'**	**Record the date and time of the fifth instance of headache.**	**text (datetime_mdy), Required**
	466	**nowalk_time_yn**<br>**Show the field ONLY if: [postbup_misc_sx(5)] = '1'**	**Did the provider document the time(s) at which inability to walk was noted?**	**yesno, Required**<br>1 Yes<br>0 No
	467	**nowalk_num**<br>**Show the field ONLY if: [nowalk_time_yn] = '1'**	**How many instances of inability to walk were documented with timestamps within 6 hours after first buprenorphine administration?**	**radio, Required**<br>0 0<br>1 1<br>2 2<br>3 3<br>4 4<br>5 5
	468	**nowalk_time1**<br>**Show the field ONLY if: [nowalk_num] = '1' or [nowalk_num] = '2' or [nowalk_num] = '3' or [nowalk_num] = '4' or [nowalk_num] = '5'**	**Record the date and time of the first instance of inability to walk.**	**text (datetime_mdy), Required**

© 2021 Herring AA et al. *JAMA Network Open.*

	469	**nowalk_time2**<br><br>**Show the field ONLY if: [nowalk_num] = '2' or [nowalk_num] = '3' or [nowalk_num] = '4' or [nowalk_num] = '5'**	**Record the date and time of the second instance of inability to walk.**	**text (datetime_mdy), Required**
	470	**nowalk_time3**<br><br>**Show the field ONLY if: [nowalk_num] = '3' or [nowalk_num] = '4' or [nowalk_num] = '5'**	**Record the date and time of the third instance of inability to walk.**	**text (datetime_mdy), Required**
	471	**nowalk_time4**<br><br>**Show the field ONLY if: [nowalk_num] = '4' or [nowalk_num] = '5'**	**Record the date and time of the fourth instance of inability to walk.**	**text (datetime_mdy), Required**
	472	**nowalk_time5**<br><br>**Show the field ONLY if: [nowalk_num] = '5'**	**Record the date and time of the fifth instance of inability to walk.**	**text (datetime_mdy), Required**
	473	**nodc_time_yn**<br><br>**Show the field ONLY if: [postbup_misc_sx(6)] = '1'**	**Did the provider document the time(s) at which inability to discharge the patient was noted?**	**yesno, Required**<br>**1 Yes**<br>**0 No**
	474	**nodc_num**<br><br>**Show the field ONLY if: [nodc_time_yn] = '1'**	**How many instances of inability to discharge were documented with timestamps within 6 hours after first buprenorphine administration?**	**radio, Required**<br>**0 0**<br>**1 1**<br>**2 2**<br>**3 3**<br>**4 4**<br>**5 5**
	475	**nodc_time1**<br><br>**Show the field ONLY if: [nodc_num] = '1' or [nodc_num] = '2' or [nodc_num] = '3' or [nodc_num] = '4' or [nodc_num] = '5'**	**Record the date and time of the first instance of inability to discharge.**	**text (datetime_mdy), Required**
	476	**nodc_time2**<br><br>**Show the field ONLY if: [nodc_num] = '2' or [nodc_num] = '3' or [nodc_num] = '4' or [nodc_num] = '5'**	**Record the date and time of the second instance of inability to discharge.**	**text (datetime_mdy), Required**
	477	**nodc_time3**<br><br>**Show the field ONLY if: [nodc_num] = '3' or [nodc_num] = '4' or [nodc_num] = '5'**	**Record the date and time of the third instance of inability to discharge.**	**text (datetime_mdy), Required**

© 2021 Herring AA et al. *JAMA Network Open.*

	478	nodc_time4<br><br>Show the field ONLY if: [nodc_num] = '4' or [nodc_num] = '5'	Record the date and time of the fourth instance of inability to discharge.	text (datetime_mdy), Required
	479	nodc_time5<br><br>Show the field ONLY if: [nodc_num] = '5'	Record the date and time of the fifth instance of inability to discharge.	text (datetime_mdy), Required
	480	miosis_time_yn<br><br>Show the field ONLY if: [postbup_misc_sx(7)] = '1'	Did the provider document the time(s) at which pupil constriction was noted?	yesno, Required<br>1 Yes<br>0 No
	481	miosis_num<br><br>Show the field ONLY if: [miosis_time_yn] = '1'	How many instances of pupil constriction were documented with timestamps within 6 hours after first buprenorphine administration?	radio, Required<br>0 0<br>1 1<br>2 2<br>3 3<br>4 4<br>5 5
	482	miosis_time1<br><br>Show the field ONLY if: [miosis_num] = '1' or [miosis_num] = '2' or [miosis_num] = '3' or [miosis_num] = '4' or [miosis_num] = '5'	Record the date and time of the first instance of pupil constriction.	text (datetime_mdy), Required
	483	postbup_miosis_mm_1<br><br>Show the field ONLY if: [miosis_num] = '1' or [miosis_num] = '2' or [miosis_num] = '3' or [miosis_num] = '4' or [miosis_num] = '5'	Pupil measurement listed?	yesno, Required<br>1 Yes<br>0 No
	484	postbup_miosis_size_1<br><br>Show the field ONLY if: [postbup_miosis_mm_1] = '1'	Please enter the pupil size measured (in mm)	text, Required
	485	miosis_time2<br><br>Show the field ONLY if: [miosis_num] = '2' or [miosis_num] = '3' or [miosis_num] = '4' or [miosis_num] = '5'	Record the date and time of the second instance of pupil constriction.	text (datetime_mdy), Required
	486	postbup_miosis_mm_2<br><br>Show the field ONLY if: [miosis_num] = '2' or [miosis_num] = '3' or [miosis_num] = '4' or [miosis_num] = '5'	Pupil measurement listed?	yesno, Required<br>1 Yes<br>0 No

© 2021 Herring AA et al. *JAMA Network Open.*

	487	**postbup_miosis_size_2**<br><br>**Show the field ONLY if: [postbup_miosis_mm_2] = '1'**	**Please enter the pupil size measured (in mm)**	**text, Required**
	488	**miosis_time3**<br><br>**Show the field ONLY if: [miosis_num] = '3' or [miosis_num] = '4' or [miosis_num] = '5'**	**Record the date and time of the third instance of pupil constriction.**	**text (datetime_mdy), Required**
	489	**postbup_miosis_mm_3**<br><br>**Show the field ONLY if: [miosis_num] = '3' or [miosis_num] = '4' or [miosis_num] = '5'**	**Pupil measurement listed?**	**yesno, Required**<br>**1 Yes**<br>**0 No**
	490	**postbup_miosis_size_3**<br><br>**Show the field ONLY if: [postbup_miosis_mm_3] = '1'**	**Please enter the pupil size measured (in mm)**	**text, Required**
	491	**miosis_time4**<br><br>**Show the field ONLY if: [miosis_num] = '4' or [miosis_num] = '5'**	**Record the date and time of the fourth instance of pupil constriction.**	**text (datetime_mdy), Required**
	492	**postbup_miosis_mm_4**<br><br>**Show the field ONLY if: [miosis_num] = '4' or [miosis_num] = '5'**	**Pupil measurement listed?**	**yesno, Required**<br>**1 Yes**<br>**0 No**
	493	**postbup_miosis_size_4**<br><br>**Show the field ONLY if: [postbup_miosis_mm_4] = '1'**	**Please enter the pupil size measured (in mm)**	**text, Required**
	494	**miosis_time5**<br><br>**Show the field ONLY if: [miosis_num] = '5'**	**Record the date and time of the fifth instance of pupil constriction.**	**text (datetime_mdy), Required**
	495	**postbup_miosis_mm_5**<br><br>**Show the field ONLY if: [miosis_num] = '5'**	**Pupil measurement listed?**	**yesno, Required**<br>**1 Yes**<br>**0 No**
	496	**postbup_miosis_size_5**<br><br>**Show the field ONLY if: [postbup_miosis_mm_5] = '1'**	**Please enter the pupil size measured (in mm)**	**text, Required**
	497	**postbup_perrl_time_yn**<br><br>**Show the field ONLY if: [postbup_misc_sx(8)] = '1'**	**Did the provider document the time(s) at which return to normal pupil size was noted?**	**yesno, Required**<br>**1 Yes**<br>**0 No**

© 2021 Herring AA et al. *JAMA Network Open.*

	498	**perrl_num**<br><br>**Show the field ONLY if: [postbup_perrl_time_yn] = '1'**	**How many instances of return to normal pupil size were documented with timestamps within 6 hours after first buprenorphine administration?**	**radio, Required**<br>0 0<br>1 1<br>2 2<br>3 3<br>4 4<br>5 5
	499	**perrl_time1**<br><br>**Show the field ONLY if: [perrl_num] = '1' or [perrl_num] = '2' or [perrl_num] = '3' or [perrl_num] = '4' or [perrl_num] = '5'**	**Record the date and time of the first instance of return to normal pupil size.**	**text (datetime_mdy), Required**
	500	**postbup_perrl_mm_1**<br><br>**Show the field ONLY if: [perrl_num] = '1' or [perrl_num] = '2' or [perrl_num] = '3' or [perrl_num] = '4' or [perrl_num] = '5'**	**Pupil measurement listed?**	**yesno, Required**<br>1 Yes<br>0 No
	501	**postbup_perrl_size_1**<br><br>**Show the field ONLY if: [postbup_perrl_mm_1] = '1'**	**Please enter the pupil size measured (in mm)**	**text, Required**
	502	**perrl_time2**<br><br>**Show the field ONLY if: [perrl_num] = '2' or [perrl_num] = '3' or [perrl_num] = '4' or [perrl_num] = '5'**	**Record the date and time of the second instance of return to normal pupil size.**	**text (datetime_mdy), Required**
	503	**postbup_perrl_mm_2**<br><br>**Show the field ONLY if: [perrl_num] = '2' or [perrl_num] = '3' or [perrl_num] = '4' or [perrl_num] = '5'**	**Pupil measurement listed?**	**yesno, Required**<br>1 Yes<br>0 No
	504	**postbup_perrl_size_2**<br><br>**Show the field ONLY if: [postbup_perrl_mm_2] = '1'**	**Please enter the pupil size measured (in mm)**	**text, Required**
	505	**perrl_time3**<br><br>**Show the field ONLY if: [perrl_num] = '3' or [perrl_num] = '4' or [perrl_num] = '5'**	**Record the date and time of the third instance of return to normal pupil size.**	**text (datetime_mdy), Required**

	506	postbup_perrl_mm_3<br><br>Show the field ONLY if: [perrl_num] = '3' or [perrl_num] = '4' or [perrl_num] = '5'	Pupil measurement listed?	yesno, Required<br>1 Yes<br>0 No
	507	postbup_perrl_size_3<br><br>Show the field ONLY if: [postbup_perrl_mm_3] = '1'	Please enter the pupil size measured (in mm)	text, Required
	508	perrl_time4<br><br>Show the field ONLY if: [perrl_num] = '4' or [perrl_num] = '5'	Record the date and time of the fourth instance of return to normal pupil size.	text (datetime_mdy), Required
	509	postbup_perrl_mm_4<br><br>Show the field ONLY if: [perrl_num] = '4' or [perrl_num] = '5'	Pupil measurement listed?	yesno, Required<br>1 Yes<br>0 No
	510	postbup_perrl_size_4<br><br>Show the field ONLY if: [postbup_perrl_mm_4] = '1'	Please enter the pupil size measured (in mm)	text, Required
	511	perrl_time5<br><br>Show the field ONLY if: [perrl_num] = '5'	Record the date and time of the fifth instance of return to normal pupil size.	text (datetime_mdy), Required
	512	postbup_perrl_mm_5<br><br>Show the field ONLY if: [perrl_num] = '5'	Pupil measurement listed?	yesno, Required<br>1 Yes<br>0 No
	513	postbup_perrl_size_5<br><br>Show the field ONLY if: [postbup_perrl_mm_5] = '1'	Please enter the pupil size measured (in mm)	text, Required
	514	late_ss	Section Header: *Late-Developed Signs and Symptoms*<br>Did any of the above signs or symptoms newly develop after the first 6 hours following first buprenorphine administration until discharge?	yesno, Required<br>1 Yes<br>0 No
	515	late_sedation<br><br>Show the field ONLY if: [late_ss] = '1'	Section Header: *Late-Developed Sedation*<br>Which of the following signs or symptoms of sedation were documented after the first 6 hours following first buprenorphine administration until discharge?	checkbox, Required<br>1 late_sedation_1 Nodding<br>2 late_sedation_2 Sleepy, drowsy, diffculty staying awake<br>3 late_sedation_3 Feelings of general fatigue or weakness<br>0 late_sedation_0 None of the above

© 2021 Herring AA et al. *JAMA Network Open.*

	516	late_intox<br><br>Show the field ONLY if:<br>[late_ss] = '1'	**Section Header:** *Late-Developed Intoxication*<br><br>Which of the following signs or symptoms of intoxication were documented after the first 6 hours following first buprenorphine administration until discharge?	checkbox, Required<br>1 | late_intox_1 | Good mood<br>2 | late_intox_2 | Coasting (Spaced out)<br>3 | late_intox_3 | Pleasant Sick<br>4 | late_intox_4 | Drive (energy)<br>5 | late_intox_5 | High<br>6 | late_intox_6 | Drunken<br>7 | late_intox_7 | Soapbox (talkative)<br>8 | late_intox_8 | Rush<br>9 | late_intox_9 | Friendly<br>10 | late_intox_10 | Relaxed<br>11 | late_intox_11 | Diffculty concentrating<br>12 | late_intox_12 | Feeling lightheaded or dizzy<br>13 | late_intox_13 | Feeling confused<br>0 | late_intox_0 | None of the above
	517	late_gi<br><br>Show the field ONLY if:<br>[late_ss] = '1'	**Section Header:** *Late-Developed GI Symptoms*<br><br>Which of the following gastrointestinal signs or symptoms were documented after the first 6 hours following first buprenorphine administration until discharge?	checkbox, Required<br>1 | late_gi_1 | Constipation<br>2 | late_gi_2 | Turning of stomach/cramping/abdominal pain<br>3 | late_gi_3 | Nausea<br>4 | late_gi_4 | Vomiting<br>5 | late_gi_5 | Diarrhea<br>0 | late_gi_0 | None of the above
	518	late_anxious<br><br>Show the field ONLY if:<br>[late_ss] = '1'	**Section Header:** *Late-Developed Anxiety*<br><br>Which of the following signs or symptoms of anxiety were documented after the first 6 hours following first buprenorphine administration until discharge?	checkbox, Required<br>1 | late_anxious_1 | Anxiety, irritability, or agitation<br>2 | late_anxious_2 | Nervousness<br>3 | late_anxious_3 | Tremor<br>4 | late_anxious_4 | Restlessness<br>0 | late_anxious_0 | None of the above

	519	**late_withdrawal**<br><br>**Show the field ONLY if: [late_ss] = '1'**	**Section Header:** *Late-Developed Withdrawal*<br><br>**Which of the following signs or symptoms of withdrawal were documented after the first 6 hours following first buprenorphine administration until discharge?**	**checkbox, Required**<br>1 late_withdrawal_1 Yawning<br>2 late_withdrawal_2 Gooseflesh skin (chills/piloerection)<br>3 late_withdrawal_3 Pulse > 80<br>4 late_withdrawal_4 Sweating (diaphoresis)<br>5 late_withdrawal_5 Pupil size larger than normal (dilation/mydriasis)<br>6 late_withdrawal_6 Bone, joint aches, or body aches (arthralgias/myalgias)<br>7 late_withdrawal_7 Runny nose or tearing (rhinorrea or lacrimation)<br>0 late_withdrawal_0 None of the above
	520	**late_vitals**<br><br>**Show the field ONLY if: [late_ss] = '1'**	**Section Header:** *Late-Developed Abnormal Vital Signs*<br><br>**Which of the following abnormal vital signs were documented after the first 6 hours following first buprenorphine administration until discharge?**	**checkbox, Required**<br>1 late_vitals_1 Hypoxia<br>2 late_vitals_2 Hypercarbia<br>3 late_vitals_3 Hypotension<br>4 late_vitals_4 Bradycardia<br>0 late_vitals_0 None of the above
	521	**late_misc_sx**<br><br>**Show the field ONLY if: [late_ss] = '1'**	**Section Header:** *Late-Developed Miscellaneous Opioid Related Side Effects*<br><br>**Which of the following miscellaneous opioid related side effects were documented after the first 6 hours following first buprenorphine administration until discharge?**	**checkbox, Required**<br>1 late_misc_sx_1 Diffculty passing urine<br>2 late_misc_sx_2 Itchiness<br>3 late_misc_sx_3 Dry mouth<br>4 late_misc_sx_4 Headache<br>5 late_misc_sx_5 Unable to walk<br>6 late_misc_sx_6 Unable to discharge<br>7 late_misc_sx_7 Pupil size smaller than normal (constriction/miosis)<br>0 late_misc_sx_0 None of the above
	522	**ed_death**	**Section Header:** *Death*<br><br>**Did the ED chart indicate that the patient died?**	**yesno, Required**<br>1 Yes<br>0 No
	523	**other_meds**	**Section Header:** *Other E.D. Medications*<br><br>**Was the patient given any medications before bup or within the first six hours after bup?**	**yesno, Required**<br>1 Yes<br>0 No

© 2021 Herring AA et al. *JAMA Network Open.*

	524	**other_meds_num**<br>**Show the field ONLY if: [other_meds] = '1'**	**How many other unique medications were given?**	**radio, Required**<br>1 1<br>2 2<br>3 3<br>4 4<br>5 5<br>6 6<br>7 7<br>8 8<br>9 9<br>10 10
	525	**other_meds_1**<br>**Show the field ONLY if: [other_meds_num] = '1' or [other_meds_num] = '2' or [other_meds_num] = '3' or [other_meds_num] = '4' or [other_meds_num] = '5' or [other_meds_num] = '6' or [other_meds_num] = '7' or [other_meds_num] = '8' or [other_meds_num] = '9' or [other_meds_num] = '10'**	**Please name the (first) medication**	**text, Required**
	526	**other_meds_2**<br>**Show the field ONLY if: [other_meds_num] = '2' or [other_meds_num] = '3' or [other_meds_num] = '4' or [other_meds_num] = '5' or [other_meds_num] = '6' or [other_meds_num] = '7' or [other_meds_num] = '8' or [other_meds_num] = '9' or [other_meds_num] = '10'**	**Please name the second medication**	**text, Required**
	527	**other_meds_3**<br>**Show the field ONLY if: [other_meds_num] = '3' or [other_meds_num] = '4' or [other_meds_num] = '5' or [other_meds_num] = '6' or [other_meds_num] = '7' or [other_meds_num] = '8' or [other_meds_num] = '9' or [other_meds_num] = '10'**	**Please name the third medication**	**text, Required**

© 2021 Herring AA et al. *JAMA Network Open.*

	528	**other_meds_4**<br><br>**Show the field ONLY if: [other_meds_num] = '4' or [other_meds_num] = '5' or [other_meds_num] = '6' or [other_meds_num] = '7' or [other_meds_num] = '8' or [other_meds_num] = '9' or [other_meds_num] ='10'**	**Please name the fourth medication**	**text, Required**
	529	**other_meds_5**<br><br>**Show the field ONLY if: [other_meds_num] = '5' or [other_meds_num] = '6' or [other_meds_num] = '7' or [other_meds_num] = '8' or [other_meds_num] = '9' or [other_meds_num] ='10'**	**Please name the fifth medication**	**text, Required**
	530	**other_meds_6**<br><br>**Show the field ONLY if: [other_meds_num] = '6' or [other_meds_num] = '7' or [other_meds_num] = '8' or [other_meds_num] = '9' or [other_meds_num] ='10'**	**Please name the sixth medication**	**text, Required**
	531	**other_meds_7**<br><br>**Show the field ONLY if: [other_meds_num] = '7' or [other_meds_num] = '8' or [other_meds_num] ='9'or[other_meds_num] ='10'**	**Please name the seventh medication**	**text, Required**
	532	**other_meds_8**<br><br>**Show the field ONLY if: [other_meds_num] = '8' or [other_meds_num] = '9' or [other_meds_num] = '10'**	**Please name the eighth medication**	**text, Required**
	533	**other_meds_9**<br><br>**Show the field ONLY if: [other_meds_num] = '9' or [other_meds_num] = '10'**	**Please name the ninth medication**	**text, Required**
	534	**other_meds_10**<br><br>**Show the field ONLY if: [other_meds_num] = '1 0'**	**Please name the tenth medication**	**text, Required**

© 2021 Herring AA et al. *JAMA Network Open.*

<table>
<tr><td></td><td>535</td><td>ancillary_ed

Show the field ONLY if:
[other_meds] = '1'</td><td>Which of the following classes of medications was the patient given during their E.D. visit?</td><td>checkbox, Required
<table><tr><td>1</td><td>ancillary_ed_1</td><td>Anti-emetics</td></tr><tr><td>2</td><td>ancillary_ed_2</td><td>Anti-diarrheals</td></tr><tr><td>3</td><td>ancillary_ed_3</td><td>Clonidine</td></tr><tr><td>4</td><td>ancillary_ed_4</td><td>Non-opioid painkillers</td></tr><tr><td>5</td><td>ancillary_ed_5</td><td>Opioids (not including buprenorphine)</td></tr><tr><td>6</td><td>ancillary_ed_6</td><td>Gabapentin</td></tr><tr><td>7</td><td>ancillary_ed_7</td><td>Psychiatric medications</td></tr><tr><td>8</td><td>ancillary_ed_8</td><td>Benzodiazepines</td></tr><tr><td>0</td><td>ancillary_ed_0</td><td>None of the above</td></tr></table></td></tr>
<tr><td></td><td>536</td><td>md_hpi</td><td>Section Header: MD Notes
MD/MLP HPI from Wellsoft
Copy and paste from MD note.</td><td>notes, Required</td></tr>
<tr><td></td><td>537</td><td>pmh</td><td>Past medical history (check all that apply)</td><td>checkbox, Required
<table><tr><td>1</td><td>pmh_1</td><td>Congestive heart failure (CHF)</td></tr><tr><td>2</td><td>pmh_2</td><td>Chronic obstructive pulmonary disease (COPD)</td></tr><tr><td>3</td><td>pmh_3</td><td>Coronary artery disease (CAD)</td></tr><tr><td>4</td><td>pmh_4</td><td>Diabetes mellitus (DM)</td></tr><tr><td>5</td><td>pmh_5</td><td>Asthma</td></tr><tr><td>6</td><td>pmh_6</td><td>HIV (check if rapid test comes back positive)</td></tr><tr><td>7</td><td>pmh_7</td><td>Hepatitis C (if known; do not check if only Ab test positive)</td></tr><tr><td>8</td><td>pmh_8</td><td>Chronic kidney disease (CKD) or end-stage renal disease (ESRD)</td></tr><tr><td>9</td><td>pmh_9</td><td>Cirrhosis</td></tr><tr><td>10</td><td>pmh_10</td><td>Hypertension (HTN)</td></tr><tr><td>11</td><td>pmh_11</td><td>Cancer</td></tr><tr><td>12</td><td>pmh_12</td><td>Seizure</td></tr><tr><td>13</td><td>pmh_13</td><td>Other</td></tr><tr><td>0</td><td>pmh_0</td><td>None of the above</td></tr></table></td></tr>
<tr><td></td><td>538</td><td>pmh_oth

Show the field ONLY if:
[pmh(13)] = '1'</td><td>If "Other," please specify.</td><td>text, Required</td></tr>
</table>

© 2021 Herring AA et al. *JAMA Network Open.*

	539	sud_hx	Other past and present substance use (check all that apply).	checkbox, Required<br>1 sud_hx_1 Methamphetamines<br>2 sud_hx_2 Cocaine<br>3 sud_hx_3 Cannabis<br>4 sud_hx_4 Hallucinogens (including Ecstasy/MDMA)<br>5 sud_hx_5 LSD<br>6 sud_hx_6 Benzodiazepines<br>7 sud_hx_7 Alcohol<br>8 sud_hx_8 Tobacco<br>9 sud_hx_9 Other stimulant use NOS<br>10 sud_hx_10 Other (note any heavy usage)<br>0 sud_hx_0 None of the above
	540	sud_hx_oth<br><br>Show the field ONLY if: [sud_hx(10)] = '1'	If "Other," please specify.	text, Required
	541	psych_admit	Does the patient have a history of admission to an inpatient psychiatric unit?<br>*E.g., John George Pavillion*	yesno, Required<br>1 Yes<br>0 No
	542	psych_disorder	Any mention of a psychiatric disorder?	yesno, Required<br>1 Yes<br>0 No
	543	psych_hx<br><br>Show the field ONLY if: [psych_disorder] = '1'	Please choose from the following psychiatric disorders.<br>*If "Other," or not speciied, choose: "Psych NOS."*	checkbox, Required<br>1 psych_hx_1 Psych NOS<br>2 psych_hx_2 Depression<br>3 psych_hx_3 Bipolar disorder<br>4 psych_hx_4 Schizophrenia<br>5 psych_hx_5 Anxiety<br>6 psych_hx_6 Post-traumatic stress disorder (PTSD)

© 2021 Herring AA et al. *JAMA Network Open.*

544	ancillary_home	Which of the following classes of prescribed supportive medications does the patient currently take?	checkbox, Required<br>1 ancillary_home_1 Anti-emetics<br>2 ancillary_home_2 Anti-diarrheals<br>3 ancillary_home_3 Clonidine<br>4 ancillary_home_4 Non-opioid painkillers<br>6 ancillary_home_6 Gabapentin<br>7 ancillary_home_7 Psychiatric medications<br>8 ancillary_home_8 Benzodiazepines<br>0 ancillary_home_0 None of the above
545	last_illicit	Last illicit opioid use known?	radio, Required<br>1 Yes<br>0 No/Not applicable
546	last_opioid_type<br><br>Show the field ONLY if: [last_illicit] = '1'	Last opioid(s) used before ED visit<br>*Brand names listed in parantheses*	checkbox, Required<br>1 last_opioid_type_1 Heroin<br>2 last_opioid_type_2 Oxycodone (OxyContin, Percocet, Roxicodone, etc.)<br>3 last_opioid_type_3 Hydrocodone (Norco, Vicodin, etc.)<br>4 last_opioid_type_4 Fentanyl (Duragesic, Ionsys)<br>5 last_opioid_type_5 Hydromorphon (Dilaudid)<br>6 last_opioid_type_6 Morphine<br>7 last_opioid_type_7 Other<br>999 last_opioid_type_999 Unknown
547	last_heroin_mx<br><br>Show the field ONLY if: [last_opioid_type(1)] = '1'	**Section Header:** *Last Heroin Use*<br>Are there discrepant values documented for when the patient last used heroin?	yesno, Required<br>1 Yes<br>0 No
548	heroin_old<br><br>Show the field ONLY if: [last_heroin_mx] = '1'	Oldest heroin use documented<br>*Enter numerical value.*	text, Required
549	heroin_new<br><br>Show the field ONLY if: [last_heroin_mx] = '1'	Most recent heroin use documented<br>*Enter numerical value.*	text, Required
550	heroin_avg<br><br>Show the field ONLY if: [last_heroin_mx] = '1'	Average	calc, Required<br>Calculation: mean([heroin_old] , [heroin_new])

© 2021 Herring AA et al. *JAMA Network Open.*

	551	last_heroin_use<br><br>Show the field ONLY if: [last_heroin_mx] = '0'	**Last heroin use**<br>*Enter 999 if unknown. Use numerical value.*	text, Required
	552	last_heroin_unit<br><br>Show the field ONLY if: [last_opioid_type(1)] = '1'	**Unit of time**	radio, Required<br>1 Hours<br>2 Days<br>3 Weeks<br>4 Months<br>5 Years<br>999 Unknown/Unclear
	553	last_heroin_amt<br><br>Show the field ONLY if: [last_opioid_type(1)] = '1'	**Is the amount of last heroin use documented?**	yesno, Required<br>1 Yes<br>0 No
	554	last_heroin_amt_type<br><br>Show the field ONLY if: [last_heroin_amt] = '1'	**Amount type**	radio, Required<br>1 grams<br>2 $<br>3 bags<br>4 pills<br>5 milligrams<br>6 micrograms
	555	last_heroin_amt_spec<br><br>Show the field ONLY if: [last_heroin_amt] = '1'	**Please specify amount used**<br>*Enter numerical value. E.g., if "2 grams," enter "2." Enter "999" if unknown.*	text, Required
	556	last_heroin_route<br><br>Show the field ONLY if: [last_opioid_type(1)] = '1'	**Route of Administration**	checkbox, Required<br>1 last_heroin_route_1 Injection<br>2 last_heroin_route_2 Snort<br>3 last_heroin_route_3 Smoke<br>999 last_heroin_route_999 Unknown
	557	last_oxy_mx<br><br>Show the field ONLY if: [last_opioid_type(2)] = '1'	**Section Header:** *Last Oxycodone Use*<br>**Are there discrepant values documented for when the patient last used oxycodone?**	yesno, Required<br>1 Yes<br>0 No
	558	oxy_old<br><br>Show the field ONLY if: [last_oxy_mx] = '1'	**Oldest oxycodone use documented**<br>*Enter numerical value.*	text, Required
	559	oxy_new<br><br>Show the field ONLY if: [last_oxy_mx] = '1'	**Most recent oxycodone use documented**<br>*Enter numerical value.*	text, Required
	560	oxy_avg<br><br>Show the field ONLY if: [last_oxy_mx] = '1'	**Average**	calc, Required<br>Calculation: mean([oxy_old] , [oxy_new])

© 2021 Herring AA et al. *JAMA Network Open.*

	561	last_oxy_use<br><br>**Show the field ONLY if:**<br>**[last_oxy_mx] ='0'**	**Last oxycodone use**<br>*Enter 999 if unknown. Use numerical value.*	**text, Required**
	562	last_oxy_unit<br><br>**Show the field ONLY if:**<br>**[last_opioid_type(2)] = '1'**	**Unit of time**	**radio, Required**<br>1 Hours<br>2 Days<br>3 Weeks<br>4 Months<br>5 Years<br>999 Unknown/Unclear
	563	last_oxy_amt<br><br>**Show the field ONLY if:**<br>**[last_opioid_type(2)] = '1'**	**Is the amount of last illicit oxycodone use documented?**	**yesno, Required**<br>1 Yes<br>0 No
	564	last_oxy_amt_type<br><br>**Show the field ONLY if:**<br>**[last_oxy_amt] = '1'**	**Amount type**	**radio, Required**<br>1 grams<br>2 $<br>3 bags<br>4 pills<br>5 milligrams<br>6 micrograms
	565	last_oxy_amt_spec<br><br>**Show the field ONLY if:**<br>**[last_oxy_amt] = '1'**	**Please specify amount used**<br>*Enter numerical value. E.g., if "2 grams," enter "2." Enter "999" if unknown.*	**text, Required**
	566	last_oxy_route<br><br>**Show the field ONLY if:**<br>**[last_opioid_type(2)] = '1'**	**Route of Administration**	**checkbox, Required**<br>1 last_oxy_route_1 Injection<br>2 last_oxy_route_2 Snort<br>3 last_oxy_route_3 Smoke<br>4 last_oxy_route_4 Swallow<br>999 last_oxy_route_999 Unknown
	567	last_hyd_mx<br><br>**Show the field ONLY if:**<br>**[last_opioid_type(3)] = '1'**	**Section Header:** *Last Hydrocodone Use*<br><br>**Are there discrepant values documented for when the patient last used hydrocodone?**	**yesno, Required**<br>1 Yes<br>0 No
	568	hyd_old<br><br>**Show the field ONLY if:**<br>**[last_hyd_mx] ='1'**	**Oldest hydrocodone use documented**<br>*Enter numerical value.*	**text, Required**
	569	hyd_new<br><br>**Show the field ONLY if:**<br>**[last_hyd_mx] ='1'**	**Most recent hydrocodone use documented**<br>*Enter numerical value.*	**text, Required**
	570	hyd_avg<br><br>**Show the field ONLY if:**<br>**[last_hyd_mx] ='1'**	**Average**	**calc, Required**<br>**Calculation: mean([hyd_old] , [hyd_new])**

	571	**last_hyd_use**<br>**Show the field ONLY if: [last_hyd_mx] ='0'**	**Last hydrocodone use**<br>*Enter 999 if unknown. Use numerical value.*	**text, Required**
	572	**last_hyd_unit**<br>**Show the field ONLY if: [last_opioid_type(3)] = '1'**	**Unit of time**	**radio, Required**<br>1 Hours<br>2 Days<br>3 Weeks<br>4 Months<br>5 Years<br>999 Unknown/Unclear
	573	**last_hyd_amt**<br>**Show the field ONLY if: [last_opioid_type(3)] = '1'**	**Is the amount of last illicit hydrocodone use documented?**	**yesno, Required**<br>1 Yes<br>0 No
	574	**last_hyd_amt_type**<br>**Show the field ONLY if: [last_hyd_amt] = '1'**	**Amount type**	**radio, Required**<br>1 grams<br>2 $<br>3 bags<br>4 pills<br>5 milligrams<br>6 micrograms
	575	**last_hyd_amt_spec**<br>**Show the field ONLY if: [last_hyd_amt] = '1'**	**Please specify amount used**<br>*Enter numerical value. E.g., if "2 grams," enter "2." Enter "999" if unknown.*	**text, Required**
	576	**last_hyd_route**<br>**Show the field ONLY if: [last_opioid_type(3)] = '1'**	**Route of Administration**	**checkbox, Required**<br>1 last_hyd_route_1 Injection<br>2 last_hyd_route_2 Snort<br>3 last_hyd_route_3 Smoke<br>4 last_hyd_route_4 Swallow<br>999 last_hyd_route_999 Unknown
	577	**last_fent_mx**<br>**Show the field ONLY if: [last_opioid_type(4)] = '1'**	**Section Header:** *Last Fentanyl Use*<br>**Are there discrepant values documented for when the patient last used fentanyl?**	**yesno, Required**<br>1 Yes<br>0 No
	578	**fent_old**<br>**Show the field ONLY if: [last_fent_mx] = '1'**	**Oldest fentanyl use documented**<br>*Enter numerical value.*	**text, Required**
	579	**fent_new**<br>**Show the field ONLY if: [last_fent_mx] = '1'**	**Most recent fentanyl use documented**<br>*Enter numerical value.*	**text, Required**
	580	**fent_avg**<br>**Show the field ONLY if: [last_fent_mx] = '1'**	**Average**	**calc, Required**<br>**Calculation: mean([fent_old] , [fent_new])**

© 2021 Herring AA et al. *JAMA Network Open.*

	581	last_fent_use<br><br>**Show the field ONLY if: [last_fent_mx] = '0'**	**Last fentanyl use**<br>*Enter 999 if unknown. Use numerical value.*	text, Required
	582	last_fent_unit<br><br>**Show the field ONLY if: [last_opioid_type(4)] = '1'**	**Unit of time**	radio, Required<br>1 Hours<br>2 Days<br>3 Weeks<br>4 Months<br>5 Years<br>999 Unknown/Unclear
	583	last_fent_amt<br><br>**Show the field ONLY if: [last_opioid_type(4)] = '1'**	**Is the amount of last illicit fentanyl use documented?**	yesno, Required<br>1 Yes<br>0 No
	584	last_fent_amt_type<br><br>**Show the field ONLY if: [last_fent_amt] = '1'**	**Amount type**	radio, Required<br>1 grams<br>2 $<br>3 bags<br>4 pills<br>5 milligrams<br>6 micrograms
	585	last_fent_amt_spec<br><br>**Show the field ONLY if: [last_fent_amt] = '1'**	**Please specify amount used**<br>*Enter numerical value. E.g., if "2 grams," enter "2." Enter "999" if unknown.*	text, Required
	586	last_fent_route<br><br>**Show the field ONLY if: [last_opioid_type(4)] = '1'**	**Route of Administration**	checkbox, Required<br>1 last_fent_route_1 Injection<br>2 last_fent_route_2 Snort<br>3 last_fent_route_3 Smoke<br>4 last_fent_route_4 Swallow<br>5 last_fent_route_5 Patch<br>999 last_fent_route_999 Unknown
	587	last_hm_mx<br><br>**Show the field ONLY if: [last_opioid_type(5)] = '1'**	**Section Header:** *Last Hydromorphone Use*<br><br>**Are there discrepant values documented for when the patient last used hydromorphone?**	yesno, Required<br>1 Yes<br>0 No
	588	hm_old<br><br>**Show the field ONLY if: [last_hm_mx] = '1'**	**Oldest hydromorphone use documented**<br>*Enter numerical value.*	text, Required
	589	hm_new<br><br>**Show the field ONLY if: [last_hm_mx] = '1'**	**Most recent hydromorphone use documented**<br>*Enter numerical value.*	text, Required

© 2021 Herring AA et al. *JAMA Network Open.*

	590	**hm_avg**<br><br>**Show the field ONLY if: [last_hm_mx] = '1'**	**Average**	**calc, Required**<br>**Calculation: mean([hm_old] , [hm_new])**
	591	**last_hm_use**<br><br>**Show the field ONLY if: [last_hm_mx] ='0'**	**Last hydromorphone use**<br>*Enter 999 if unknown. Use numerical value.*	**text, Required**
	592	**last_hm_unit**<br><br>**Show the field ONLY if: [last_opioid_type(5)] = '1'**	**Unit of time**	**radio, Required**<br>1 Hours<br>2 Days<br>3 Weeks<br>4 Months<br>5 Years<br>999 Unknown/Unclear
	593	**last_hm_amt**<br><br>**Show the field ONLY if: [last_opioid_type(5)] = '1'**	**Is the amount of last illicit hydromorphone use documented?**	**yesno, Required**<br>1 Yes<br>0 No
	594	**last_hm_amt_type**<br><br>**Show the field ONLY if: [last_hm_amt] = '1'**	**Amount type**	**radio, Required**<br>1 grams<br>2 $<br>3 bags<br>4 pills<br>5 milligrams<br>6 micrograms
	595	**last_hm_amt_spec**<br><br>**Show the field ONLY if: [last_hm_amt] ='1'**	**Please specify amount used**<br>*Enter numerical value. E.g., if "2 grams," enter "2." Enter "999" if unknown.*	**text, Required**
	596	**last_hm_route**<br><br>**Show the field ONLY if: [last_opioid_type(5)] = '1'**	**Route of Administration**	**checkbox, Required**<br>1 last_hm_route_1 Injection<br>2 last_hm_route_2 Snort<br>3 last_hm_route_3 Smoke<br>4 last_hm_route_4 Swallow<br>999 last_hm_route_999 Unknown
	597	**last_mor_mx**<br><br>**Show the field ONLY if: [last_opioid_type(6)] = '1'**	**Section Header:** *Last Morphine Use*<br>**Are there discrepant values documented for when the patient last used morphine?**	**yesno, Required**<br>1 Yes<br>0 No
	598	**mor_old**<br><br>**Show the field ONLY if: [last_mor_mx] ='1'**	**Oldest morphine use documented**<br>*Enter numerical value.*	**text, Required**
	599	**mor_new**<br><br>**Show the field ONLY if: [last_mor_mx] ='1'**	**Most recent morphine use documented**<br>*Enter numerical value.*	**text, Required**

© 2021 Herring AA et al. *JAMA Network Open.*

	600	mor_avg<br><br>Show the field ONLY if:<br>[last_mor_mx] = '1'	Average	calc, Required<br>Calculation: mean([mor_old] , [mor_new])
	601	last_mor_use<br><br>Show the field ONLY if:<br>[last_mor_mx] ='0'	Last morphine use<br>*Enter 999 if unknown. Use numerical value.*	text, Required
	602	last_mor_unit<br><br>Show the field ONLY if:<br>[last_opioid_type(6)] = '1'	Unit of time	radio, Required<br>1 Hours<br>2 Days<br>3 Weeks<br>4 Months<br>5 Years<br>999 Unknown/Unclear
	603	last_mor_amt<br><br>Show the field ONLY if:<br>[last_opioid_type(6)] = '1'	Is the amount of last illicit morphine use documented?	yesno, Required<br>1 Yes<br>0 No
	604	last_mor_amt_type<br><br>Show the field ONLY if:<br>[last_mor_amt] = '1'	Amount type	radio, Required<br>1 grams<br>2 $<br>3 bags<br>4 pills<br>5 milligrams<br>6 micrograms
	605	last_mor_amt_spec<br><br>Show the field ONLY if:<br>[last_mor_amt] = '1'	Please specify amount used<br>*Enter numerical value. E.g., if "2 grams," enter "2." Enter "999" if unknown.*	text, Required
	606	last_mor_route<br><br>Show the field ONLY if:<br>[last_opioid_type(6)] = '1'	Route of Administration	checkbox, Required<br>1 last_mor_route_1 Injection<br>2 last_mor_route_2 Snort<br>3 last_mor_route_3 Smoke<br>4 last_mor_route_4 Swallow<br>999 last_mor_route_999 Unknown
	607	last_opioid_oth<br><br>Show the field ONLY if:<br>[last_opioid_type(7)] = '1'	**Section Header:** *Last [last_opioid_oth] Use*<br>If "Other" opioid was selected, please specify.	text, Required
	608	last_oth_mx<br><br>Show the field ONLY if:<br>[last_opioid_type(7)] = '1'	Are there discrepant values documented for when the patient last used [last_opioid_oth]?	yesno, Required<br>1 Yes<br>0 No

© 2021 Herring AA et al. *JAMA Network Open.*

	609	oth_old<br>Show the field ONLY if: [last_oth_mx] ='1'	**Oldest use documented**<br>*Enter numerical value.*	text, Required
	610	oth_new<br>Show the field ONLY if: [last_oth_mx] = '1'	**Most recent use documented**<br>*Enter numerical value.*	text, Required
	611	oth_avg<br>Show the field ONLY if: [last_oth_mx] = '1'	**Average**	calc, Required<br>Calculation: m ean([oth_old] , [oth_new])
	612	last_oth_use<br>Show the field ONLY if: [last_oth_mx] ='0'	**Last use**<br>*Enter 999 if unknown. Use numerical value.*	text, Required
	613	last_oth_unit<br>Show the field ONLY if: [last_opioid_type(7)] = '1'	**Unit of time**	radio, Required<br>1 Hours<br>2 Days<br>3 Weeks<br>4 Months<br>5 Years<br>999 Unknown/Unclear
	614	last_oth_amt<br>Show the field ONLY if: [last_opioid_type(7)] = '1'	**Is the amount of last illicit use documented for [last_opioid_oth]?**	yesno, Required<br>1 Yes<br>0 No
	615	last_oth_amt_type<br>Show the field ONLY if: [last_oth_amt] = '1'	**Amount type**	radio, Required<br>1 grams<br>2 $<br>3 bags<br>4 pills<br>5 milligrams<br>6 micrograms
	616	last_oth_amt_spec<br>Show the field ONLY if: [last_oth_amt] = '1'	**Please specify amount used**<br>*Enter numerical value. E.g., if "2 grams," enter "2." Enter "999" if unknown.*	text, Required
	617	last_oth_route<br>Show the field ONLY if: [last_opioid_type(7)] = '1'	**Route of Administration**	checkbox, Required
	618	last_unk_mx<br>Show the field ONLY if: [last_opioid_type(999)] = '1'	**Section Header:** *Last Unspeciied Opioid Use*<br>**Are there discrepant values documented for when the patient last used this unknown/unspecified opioid?**	

© 2021 Herring AA et al. *JAMA Network Open.*

4	last_oth_route_4	Swallow
999	last_oth_route_999	Unknown

yesno, Required

1	Yes
0	No

© 2021 Herring AA et al. *JAMA Network Open.*

	619	unk_old<br><br>Show the field ONLY if:<br>[last_unk_mx] = '1'	**Oldest use documented**<br>*Enter numerical value.*	text, Required
	620	unk_new<br><br>Show the field ONLY if:<br>[last_unk_mx] = '1'	**Most recent use documented**<br>*Enter numerical value.*	text, Required
	621	unk_avg<br><br>Show the field ONLY if:<br>[last_unk_mx] = '1'	**Average**	calc, Required<br>Calculation: mean([unk_old] , [unk_new])
	622	last_unk_use<br><br>Show the field ONLY if:<br>[last_unk_mx] = '0'	**Last use**<br>*Enter 999 if unknown. Use numerical value.*	text, Required
	623	last_unk_unit<br><br>Show the field ONLY if:<br>[last_opioid_type(999)] = '1'	**Unit of time**	radio, Required<br>1 Hours<br>2 Days<br>3 Weeks<br>4 Months<br>5 Years<br>999 Unknown/Unclear
	624	od	Section Header: *Overdose*<br>**Any mention of substance overdose in this chart? ******<br>*Includes non-opioid substances (e.g., stimulants, alcohol, etc.)*	radio, Required<br>1 Yes<br>0 No
	625	od_spec<br><br>Show the field ONLY if:<br>[od] = '1'	**Please specify the overdose:**	checkbox, Required<br>1 od_spec_1 Opioid<br>2 od_spec_2 Intoxication/overdose from other substance<br>999 od_spec_999 Unknown
	626	od_intox<br><br>Show the field ONLY if:<br>[od_spec(2)] = '1'	**Which of the following substances were mentioned in the overdose documentation (check all that apply):**	checkbox, Required<br>1 od_intox_1 Alcohol<br>2 od_intox_2 Benzodiazepines<br>3 od_intox_3 Methamphetamines<br>4 od_intox_4 Cocaine<br>5 od_intox_5 Other
	627	od_oth<br><br>Show the field ONLY if:<br>[od_intox(5)] = '1'	**If "Other," please specify.**	text, Required
	628	mat_notes	Section Header: *MAT Notes*<br>**Wellsoft notes by MAT coordinator (Christian Hailozian), if any.**<br>*Copy and paste from Other Notes.*	notes

© 2021 Herring AA et al. *JAMA Network Open.*

629	previous_bup	**Section Header:** *Buprenorphine Treatment History *****<br>**Has the patient ever been exposed to buprenorphine?**	**radio, Required**<br>1 Yes<br>0 No/Unclear
630	bup_setting<br><br>Show the field ONLY if:<br>[previous_bup] ='1'	Setting of exposure	**checkbox, Required**<br>1 bup_setting_ 1 Healthcare setting<br>2 bup_setting_ 2 Street<br>999 bup_setting 999 Unknown/Unclear
631	bup_form<br>Show the field ONLY if:<br>[previous_bup] = '1'	Formulation of most recent buprenorphine treatment (e.g., Suboxone, Subutex, etc.)	**radio, Required**<br>1 Buprenorphine/Naloxone<br>2 Buprenorphine<br>999 Unknown
632	prev_bup_amt<br>Show the field ONLY if:<br>[previous_bup] = '1'	Amount/strength of buprenorphine treatment known?	**yesno, Required**<br>1 Yes<br>0 No
633	prev_bup_freq<br>Show the field ONLY if:<br>[previous_bup] = '1'	Frequency of buprenorphine treatment known?	**yesno, Required**<br>1 Yes<br>0 No
634	prev_bup_mg<br>Show the field ONLY if:<br>[prev_bup_amt] = '1' an d [prev_bup_freq] = '1'	Strength of buprenorphine treatment (in mg)<br>*E.g., if patient takes 8mg buprenorphine TID, enter "8."*	**text, Required**
635	prev_bup_mg_tot<br>Show the field ONLY if:<br>[prev_bup_amt] = '1' an d [prev_bup_freq] = '0'	Total amount of buprenorphine treatment (in mg)	**text, Required**
636	bup_tx_route<br>Show the field ONLY if:<br>[previous_bup] = '1'	Route of most recent buprenorphine treatment	**radio, Required**<br>1 SL<br>2 BUCC<br>3 TD<br>4 IJ<br>5 IV<br>6 IM<br>7 ID<br>8 SQ<br>9 Other<br>999 Unknown
637	bup_tx_route_oth<br>Show the field ONLY if:<br>[bup_tx_route] ='9'	If "Other," please specify.	**text, Required**

© 2021 Herring AA et al. *JAMA Network Open.*

	638	**bup_freq**<br><br>**Show the field ONLY if: [prev_bup_freq] = '1'**	**Frequency of most recent treatment**	**radio, Required**<br>1 QD<br>2 Every other day<br>3 BID<br>4 TID<br>5 QID<br>6 QH<br>7 QHS<br>8 Q4h<br>9 Q4-6h<br>10 QWK<br>11 Other
	639	**bup_tx_freq_oth**<br><br>**Show the field ONLY if: [bup_freq] ='11'**	**If "Other," please specify.**	**text, Required**
	640	**bup_length_doc**<br><br>**Show the field ONLY if: [previous_bup] = '1'**	**Is there documentation of how long the patient has been engaged in treatment with buprenorphine?**	**yesno, Required**<br>1 Yes<br>0 No
	641	**bup_length_mx**<br><br>**Show the field ONLY if: [bup_length_doc] = '1'**	**Are there discrepant accounts in the chart for the length of time that this patient was engaged in treatment?**	**yesno, Required**<br>1 Yes<br>0 No
	642	**bup_length_max**<br><br>**Show the field ONLY if: [bup_length_mx] = '1'**	**Greatest length of treatment documented**<br>*Enter numerical value. E.g., if "5 months," enter "5."*	**text, Required**
	643	**bup_length_min**<br><br>**Show the field ONLY if: [bup_length_mx] = '1'**	**Shortest length of treatment documented**<br>*Enter numerical value. E.g., if "5 months," enter "5."*	**text, Required**
	644	**bup_length_avg**<br><br>**Show the field ONLY if: [bup_length_mx] = '1'**	**Average length of treatment**	**calc, Required**<br>**Calculation: mean([bup_length_max] , [bup_length_min])**
	645	**bup_length**<br><br>**Show the field ONLY if: [bup_length_mx] = '0'**	**Length of time of most recent treatment engagement**<br>*If 5 years, enter "5." Enter "999" if unknown.*	**text, Required**
	646	**bup_unit**<br><br>**Show the field ONLY if: [bup_length_doc] = '1'**	**Unit of time**	**radio, Required**<br>1 Days<br>2 Weeks<br>3 Months<br>4 Years<br>999 Unknown

© 2021 Herring AA et al. *JAMA Network Open.*

	647	methadone	**Section Header:** *Methadone Treatment History *****<br>**Any previous exposure to methadone?**	radio, Required<br>1 Yes<br>0 No/Unclear
	648	met_setting<br><br>Show the field ONLY if: [methadone] = '1'	Setting of exposure	checkbox, Required<br>1 met_setting_1 Healthcare setting<br>2 met_setting_2 Street<br>999 met_setting_999 Unknown/Unclear
	649	any_current_opioid	**Section Header:** *Current Opioid Use *****<br>**Any documentation of current opioid use?**	yesno, Required<br>1 Yes<br>0 No
	650	primary_opioid<br><br>Show the field ONLY if: [any_current_opioid] = '1'	Primary type of opioid used?	radio, Required<br>1 Heroin<br>2 Oxycodone (OxyContin, Percocet, etc.)<br>3 Hydrocodone (Norco, Vicodin, etc.)<br>4 Fentanyl (Duragesic, Ionsys)<br>5 Hydromorphone (Dilaudid)<br>6 Morphine<br>7 Other
	651	primary_opioid_oth<br><br>Show the field ONLY if: [primary_opioid] = '7'	If "Other," please specify.	text, Required
	652	curr_opioid_type<br><br>Show the field ONLY if: [any_current_opioid] = '1'	**Other opioid(s) currently used?**<br>*Brand names listed in parantheses*	checkbox, Required<br>1 curr_opioid_type_1 Heroin<br>2 curr_opioid_type_2 Oxycodone (OxyContin, Percocet, Roxicodone, etc.)<br>3 curr_opioid_type_3 Hydrocodone (Norco, Vicodin, etc.)<br>4 curr_opioid_type_4 Fentanyl (Duragesic, Ionsys)<br>5 curr_opioid_type_5 Hydromorphone (Dilaudid)<br>6 curr_opioid_type_6 Morphine<br>7 curr_opioid_type_7 Other<br>0 curr_opioid_type_0 None of the above
	653	curr_opioid_oth<br><br>Show the field ONLY if: [curr_opioid_type(7)] = '1'	If "Other," please specify.	text, Required

© 2021 Herring AA et al. *JAMA Network Open.*

	654 her<br><br>Show the field ONLY if: [primary_opioid] = '1' or [curr_opioid_type(1)] = '1'	**Section Header:** *Opioid Use Details Check the box if "Yes"*<br>Heroin	checkbox<br>1 her_1 Typical amount known?<br>2 her_2 Route known?<br>3 her_3 Frequency known?<br>4 her_4 Did the patient/provider specify how long the patient has been using this opioid?<br>5 her_5 Age of first use?
	655 oxy<br><br>Show the field ONLY if: [primary_opioid] = '2' or [curr_opioid_type(2)] = '1'	Oxycodone	checkbox<br>1 oxy_1 Typical amount known?<br>2 oxy_2 Route known?<br>3 oxy_3 Frequency known?<br>4 oxy_4 Did the patient/provider specify how long the patient has been using this opioid?<br>5 oxy_5 Age of first use?
	656 hyd<br><br>Show the field ONLY if: [primary_opioid] = '3' or [curr_opioid_type(3)] = '1'	Hydrocodone	checkbox<br>1 hyd_1 Typical amount known?<br>2 hyd_2 Route known?<br>3 hyd_3 Frequency known?<br>4 hyd_4 Did the patient/provider specify how long the patient has been using this opioid?<br>5 hyd_5 Age of first use?
	657 fent<br><br>Show the field ONLY if: [primary_opioid] = '4' or [curr_opioid_type(4)] = '1'	Fentanyl	checkbox<br>1 fent_1 Typical amount known?<br>2 fent_2 Route known?<br>3 fent_3 Frequency known?<br>4 fent_4 Did the patient/provider specify how long the patient has been using this opioid?<br>5 fent_5 Age of first use?
	658 hm<br><br>Show the field ONLY if: [primary_opioid] = '5' or [curr_opioid_type(5)] = '1'	Hydromorphone	checkbox<br>1 hm_1 Typical amount known?<br>2 hm_2 Route known?<br>3 hm_3 Frequency known?<br>4 hm_4 Did the patient/provider specify how long the patient has been using this opioid?<br>5 hm_5 Age of first use?

© 2021 Herring AA et al. *JAMA Network Open.*

	659	**mor**<br><br>**Show the field ONLY if: [primary_opioid] = '6' or [curr_opioid_type(6)] = '1'**	**Morphine**	**checkbox**<br>1 mor_1 Typical amount known?<br>2 mor_2 Route known?<br>3 mor_3 Frequency known?<br>4 mor_4 Did the patient/provider specify how long the patient has been using this opioid?<br>5 mor_5 Age of first use?
	660	**oth_primary**<br><br>**Show the field ONLY if: [primary_opioid] = '7'**	**[primary_opioid_oth]**	**checkbox**<br>1 oth_primary_1 Typical amount known?<br>2 oth_primary_2 Route known?<br>3 oth_primary_3 Frequency known?<br>4 oth_primary_4 Did the patient/provider specify how long the patient has been using this opioid?<br>5 oth_primary_5 Age of first use?
	661	**oth_other**<br><br>**Show the field ONLY if: [curr_opioid_type(7)] = '1'**	**[curr_opioid_oth]**	**checkbox**<br>1 oth_other_1 Typical amount known?<br>2 oth_other_2 Route known?<br>3 oth_other_3 Frequency known?<br>4 oth_other_4 Did the patient/provider specify how long the patient has been using this opioid?<br>5 oth_other_5 Age of first use?
	662	**her_amt_type**<br><br>**Show the field ONLY if: [her(1)] = '1'**	**Section Header:** *Current Heroin Use*<br>**Heroin amount type**<br>*if 10 grams, select "grams."*	**checkbox, Required**<br>1 her_amt_type_1 grams<br>2 her_amt_type_2 $<br>3 her_amt_type_3 bags
	663	**her_gram**<br><br>**Show the field ONLY if: [her_amt_type(1)] = '1'**	**Maximum grams of heroin documented in the chart**<br>*Enter numerical value. Use decimal value if fraction given.*	**text, Required**
	664	**her_dollar**<br><br>**Show the field ONLY if: [her_amt_type(2)] = '1'**	**Maximum dollar amount of heroin documented in the chart.**<br>*Enter numerical value.*	**text, Required**
	665	**her_bag**<br><br>**Show the field ONLY if: [her_amt_type(3)] = '1'**	**Maximum number of bags of heroin documented in the chart.**<br>*Enter numerical value.*	**text, Required**

	666	her_route<br><br>Show the field ONLY if:<br>[her(2)] = '1'	Heroin route of administration	checkbox, Required<br>1 her_route_1 Injection<br>2 her_route_2 Snort<br>3 her_route_3 Smoke
	667	her_freq<br><br>Show the field ONLY if:<br>[her(3)] = '1'	Heroin frequency	radio, Required<br>1 Multiple times per day<br>2 Daily<br>3 Weekly<br>4 Monthly<br>999 Unknown
	668	her_length_mx<br><br>Show the field ONLY if:<br>[her(4)] = '1'	Are there discrepant accounts for length of use of heroin documented in the chart?	yesno, Required<br>1 Yes<br>0 No
	669	her_length_max<br><br>Show the field ONLY if:<br>[her_length_mx] = '1'	Maximum length of use documented<br><br>*Enter numerical value.*	text, Required
	670	her_length_min<br><br>Show the field ONLY if:<br>[her_length_mx] = '1'	Minimum length of use documented<br>*Enter numerical value.*	text, Required
	671	her_length_avg<br><br>Show the field ONLY if:<br>[her_length_mx] = '1'	Average length of use	calc, Required<br>Calculation: mean([her_length_max] , [her_length_min])
	672	her_length_1<br><br>Show the field ONLY if:<br>[her_length_mx] = '0'	Length of use<br>*If 5 years, enter "5." Enter "999" if unknown.*	text, Required
	673	her_length_2<br><br>Show the field ONLY if:<br>[her(4)] = '1'	Unit of time<br>*If 5 years, select "Years."*	radio, Required<br>1 Days<br>2 Weeks<br>3 Months<br>4 Years<br>999 Unknown
	674	her_age<br><br>Show the field ONLY if:<br>[her(5)] = '1'	Age of first use	text, Required
	675	oxy_use<br><br>Show the field ONLY if:<br>[primary_opioid] = '2' or [curr_opioid_type(2)] = '1'	**Section Header:** *Current Oxycodone Use*<br>Is this use of oxycodone prescribed, illicit, or both?	radio, Required<br>1 Prescribed<br>2 Illicit<br>3 Both<br>999 Unknown/Unclear

© 2021 Herring AA et al. *JAMA Network Open.*

	676	**oxy_amt_type**<br><br>**Show the field ONLY if: [oxy(1)] = '1'**	**Oxycodone amount type**<br>*if 10 grams, select "grams."*	**checkbox, Required**<br>1 oxy_amt_type_1 milligrams<br>2 oxy_amt_type_2 $<br>3 oxy_amt_type_3 pills
	677	**oxy_mg**<br><br>**Show the field ONLY if: [oxy_amt_type(1)] = '1'**	**Maximum milligram amount of oxycodone documented in the chart**<br>*Enter numerical value. Use decimal value if fraction given.*	**text, Required**
	678	**oxy_dollar**<br><br>**Show the field ONLY if: [oxy_amt_type(2)] = '1'**	**Maximum dollar amount of oxycodone documented in the chart**<br>*Enter numerical value.*	**text, Required**
	679	**oxy_pills**<br><br>**Show the field ONLY if: [oxy_amt_type(3)] = '1'**	**Maximum number of oxycodone pills documented in the chart**<br>*Enter numerical value.*	**text, Required**
	680	**oxy_route**<br><br>**Show the field ONLY if: [oxy(2)] = '1'**	**Oxycodone route of administration**	**checkbox, Required**<br>1 oxy_route_1 Injection<br>2 oxy_route_2 Snort<br>3 oxy_route_3 Smoke<br>4 oxy_route_4 Swallow
	681	**oxy_freq**<br><br>**Show the field ONLY if: [oxy(3)] = '1'**	**Oxycodone frequency**	**radio, Required**<br>1 Multiple times per day<br>2 Daily<br>3 Weekly<br>4 Monthly
	682	**oxy_length_mx**<br><br>**Show the field ONLY if: [oxy(4)] = '1'**	**Are there discrepant accounts for length of use of oxycodone documented in the chart?**	**yesno, Required**<br>1 Yes<br>0 No
	683	**oxy_length_max**<br><br>**Show the field ONLY if: [oxy_length_mx] = '1'**	**Maximum length of use documented**<br>*Enter numerical value.*	**text, Required**
	684	**oxy_length_min**<br><br>**Show the field ONLY if: [oxy_length_mx] = '1'**	**Minimum length of use documented**<br>*Enter numerical value.*	**text, Required**
	685	**oxy_length_avg**<br><br>**Show the field ONLY if: [oxy_length_mx] = '1'**	**Average length of use**	**calc, Required**<br>**Calculation: mean([oxy_length_max] , [oxy_length_min])**
	686	**oxy_length_1**<br><br>**Show the field ONLY if: [oxy_length_mx] = '0'**	**Length of use**<br>*If 5 years, enter "5." Enter "999" if unknown.*	**text, Required**

© 2021 Herring AA et al. *JAMA Network Open.*

	687	**oxy_length_2**<br><br>**Show the field ONLY if: [oxy(4)] = '1'**	**Unit of time**<br>*If 5 years, select "Years."*	**radio, Required**<br>1 Days<br>2 Weeks<br>3 Months<br>4 Years<br>999 Unknown
	688	**oxy_age**<br><br>**Show the field ONLY if: [oxy(5)] = '1'**	**Age of first use**	**text, Required**
	689	**hyd_use**<br><br>**Show the field ONLY if: [primary_opioid] = '3' or [curr_opioid_type(3)] = '1'**	**Section Header:** *Current Hydrocodone Use*<br>**Is this use of hydrocodone prescribed, illicit, or both?**	**radio, Required**<br>1 Prescribed<br>2 Illicit<br>3 Both<br>999 Unknown/Unclear
	690	**hyd_amt_type**<br><br>**Show the field ONLY if: [hyd(1)] = '1'**	**Hydrocodone amount type**<br>*if 10 grams, select "grams."*	**checkbox, Required**<br>1 hyd_amt_type_1 milligrams<br>2 hyd_amt_type_2 $<br>3 hyd_amt_type_3 pills
	691	**hyd_mg**<br><br>**Show the field ONLY if: [hyd_amt_type(1)] = '1'**	**Maximum milligram amount of hydrocodone documented in the chart**<br>*Enter numerical value. Use decimal value if fraction given.*	**text, Required**
	692	**hyd_max_2**<br><br>**Show the field ONLY if: [hyd_amt_type(2)] = '1'**	**Maximum dollar amount of hydrocodone documented in the chart**<br>*Enter numerical value.*	**text, Required**
	693	**hyd_max_3**<br><br>**Show the field ONLY if: [hyd_amt_type(3)] = '1'**	**Maximum number of hydrocodone pills documented in the chart**<br>*Enter numerical value.*	**text, Required**
	694	**hyd_route**<br><br>**Show the field ONLY if: [hyd(2)] = '1'**	**Hydrocodone route of administration**	**checkbox, Required**<br>1 hyd_route_1 Injection<br>2 hyd_route_2 Snort<br>3 hyd_route_3 Smoke<br>4 hyd_route_4 Swallow
	695	**hyd_freq**<br><br>**Show the field ONLY if: [hyd(3)] = '1'**	**Hydrocodone frequency**	**radio, Required**<br>1 Multiple times per day<br>2 Daily<br>3 Weekly<br>4 Monthly
	696	**hyd_length_mx**<br><br>**Show the field ONLY if: [hyd(4)] = '1'**	**Are there discrepant accounts for length of use of hydrocodone documented in the chart?**	**yesno, Required**<br>1 Yes<br>0 No

© 2021 Herring AA et al. *JAMA Network Open.*

	697	**hyd_length_max**<br><br>**Show the field ONLY if: [hyd_length_mx] = '1'**	**Maximum length of use documented**<br>*Enter numerical value.*	**text, Required**
	698	**hyd_length_min**<br><br>**Show the field ONLY if: [hyd_length_mx] = '1'**	**Minimum length of use documented**<br>*Enter numerical value.*	**text, Required**
	699	**hyd_length_avg**<br><br>**Show the field ONLY if: [hyd_length_mx] = '1'**	**Average length of use**	**calc, Required**<br>**Calculation: mean([hyd_length_max] , [hyd_length_min])**
	700	**hyd_length_1**<br><br>**Show the field ONLY if: [hyd_length_mx] = '0'**	**Length of use**<br>*If 5 years, enter "5." Enter "999" if unknown.*	**text, Required**
	701	**hyd_length_2**<br><br>**Show the field ONLY if: [hyd(4)] = '1'**	**Unit of time**<br>*If 5 years, select "Years."*	**radio, Required**<br>1 Days<br>2 Weeks<br>3 Months<br>4 Years<br>999 Unknown
	702	**hyd_age**<br><br>**Show the field ONLY if: [hyd(5)] = '1'**	**Age of first use**	**text, Required**
	703	**fent_use**<br><br>**Show the field ONLY if: [primary_opioid] = '4' or [curr_opioid_type(4)] = '1'**	**Section Header:** *Current Fentanyl Use*<br>**Is this use of fentanyl prescribed, illicit, or both?**	**radio, Required**<br>1 Prescribed<br>2 Illicit<br>3 Both<br>999 Unknown/Unclear
	704	**fent_amt_type**<br><br>**Show the field ONLY if: [fent(1)] = '1'**	**Fentanyl amount type**<br>*if 10 grams, select "grams."*	**checkbox, Required**<br>1 fent_amt_type_1 micrograms<br>2 fent_amt_type_2 $<br>3 fent_amt_type_3 grams
	705	**fent_mcg**<br><br>**Show the field ONLY if: [fent_amt_type(1)] = '1'**	**Maximum microgram amount of fentanyl documented in the chart**<br>*Enter numerical value. Use decimal value if fraction given.*	**text, Required**
	706	**fent_dollar**<br><br>**Show the field ONLY if: [fent_amt_type(2)] = '1'**	**Maximum dollar amount of fentanyl documented in the chart**<br>*Enter numerical value.*	**text, Required**
	707	**fent_gram**<br><br>**Show the field ONLY if: [fent_amt_type(3)] = '1'**	**Maximum gram amount of fentanyl documented in the chart**<br>*Enter numerical value.*	**text, Required**

© 2021 Herring AA et al. *JAMA Network Open.*

	708	fent_route<br>Show the field ONLY if: [fent(2)] = '1'	Fentanyl route of administration	checkbox, Required<br>1 fent_route_1 Injection<br>2 fent_route_2 Snort<br>3 fent_route_3 Smoke<br>4 fent_route_4 Swallow<br>5 fent_route_5 Patch
	709	fent_freq<br>Show the field ONLY if: [fent(3)] = '1'	Fentanyl frequency	radio, Required<br>1 Multiple times per day<br>2 Daily<br>3 Weekly<br>4 Monthly
	710	fent_length_mx<br>Show the field ONLY if: [fent(4)] = '1'	Are there discrepant accounts for length of use of fentanyl documented in the chart?	yesno, Required<br>1 Yes<br>0 No
	711	fent_length_max<br>Show the field ONLY if: [fent_length_mx] = '1'	Maximum length of use documented<br>*Enter numerical value.*	text, Required
	712	fent_length_min<br>Show the field ONLY if: [fent_length_mx] = '1'	Minimum length of use documented<br>*Enter numerical value.*	text, Required
	713	fent_length_avg<br>Show the field ONLY if: [fent_length_mx] = '1'	Average length of use	calc, Required<br>Calculation: mean([fent_length_max] , [fent_length_min])
	714	fent_length_1<br>Show the field ONLY if: [fent_length_mx] = '0'	Length of use<br>*If 5 years, enter "5." Enter "999" if unknown.*	text, Required
	715	fent_length_2<br>Show the field ONLY if: [fent(4)] = '1'	Unit of time<br>*If 5 years, select "Years."*	radio, Required<br>1 Days<br>2 Weeks<br>3 Months<br>4 Years<br>999 Unknown
	716	fent_age<br>Show the field ONLY if: [fent(5)] = '1'	Age of first use	text, Required
	717	hm_use<br>Show the field ONLY if: [primary_opioid] = '5' or [curr_opioid_type(5)] = '1'	Section Header: *Current Hydromorphone Use*<br>Is this use of hydromorphone prescribed, illicit, or both?	radio, Required<br>1 Prescribed<br>2 Illicit<br>3 Both<br>999 Unknown/Unclear

© 2021 Herring AA et al. *JAMA Network Open.*

	718	**hm_amt_type**<br><br>**Show the field ONLY if: [hm(1)] = '1'**	**Hydromorphone amount type**<br>*if 10 grams, select "grams."*	**checkbox, Required**<br>1 hm_amt_type_1 milligrams<br>2 hm_amt_type_2 $<br>3 hm_amt_type_3 pills
	719	**hm_mg**<br><br>**Show the field ONLY if: [hm_amt_type(1)] = '1'**	**Maximum milligram amount of hydromorphone documented in the chart**<br>*Enter numerical value. Use decimal value if fraction given.*	**text, Required**
	720	**hm_dollar**<br><br>**Show the field ONLY if: [hm_amt_type(2)] = '1'**	**Maximum dollar amount of hydromorphone documented in the chart**<br>*Enter numerical value.*	**text, Required**
	721	**hm_pills**<br><br>**Show the field ONLY if: [hm_amt_type(3)] = '1'**	**Maximum number of hydromorphone pills documented in the chart**<br>*Enter numerical value.*	**text, Required**
	722	**hm_route**<br><br>**Show the field ONLY if: [hm(2)] = '1'**	**Hydromorphone route of administration**	**checkbox, Required**<br>1 hm_route_1 Injection<br>2 hm_route_2 Snort<br>3 hm_route_3 Smoke<br>4 hm_route_4 Swallow
	723	**hm_freq**<br><br>**Show the field ONLY if: [hm(3)] = '1'**	**Hydromorphone frequency**	**radio, Required**<br>1 Multiple times per day<br>2 Daily<br>3 Weekly<br>4 Monthly
	724	**hm_length_mx**<br><br>**Show the field ONLY if: [hm(4)] = '1'**	**Are there discrepant accounts for length of use of hydromorphone documented in the chart?**	**yesno, Required**<br>1 Yes<br>0 No
	725	**hm_length_max**<br><br>**Show the field ONLY if: [hm_length_mx] = '1'**	**Maximum length of use documented**<br>*Enter numerical value.*	**text, Required**
	726	**hm_length_min**<br><br>**Show the field ONLY if: [hm_length_mx] = '1'**	**Minimum length of use documented**<br>*Enter numerical value.*	**text, Required**
	727	**hm_length_avg**<br><br>**Show the field ONLY if: [hm_length_mx] = '1'**	**Average length of use**	**calc, Required**<br>**Calculation: mean([hm_length_max] , [hm_length_min])**
	728	**hm_length_1**<br><br>**Show the field ONLY if: [hm_length_mx] = '0'**	**Length of use**<br>*If 5 years, enter "5." Enter "999" if unknown.*	**text, Required**

© 2021 Herring AA et al. *JAMA Network Open.*

	729	hm_length_2<br><br>**Show the field ONLY if:**<br>**[hm(4)] = '1'**	**Unit of time**<br>*If 5 years, select "Years."*	**radio, Required**<br>1 Days<br>2 Weeks<br>3 Months<br>4 Years<br>999 Unknown
	730	hm_age<br><br>**Show the field ONLY if:**<br>**[hm(5)] = '1'**	**Age of first use**	**text, Required**
	731	mor_use<br><br>**Show the field ONLY if:**<br>**[primary_opioid] = '6' or [curr_opioid_type(6)] = '1'**	**Section Header:** *Current Morphine Use*<br>**Is this use of morphine prescribed, illicit, or both?**	**radio, Required**<br>1 Prescribed<br>2 Illicit<br>3 Both<br>999 Unknown/Unclear
	732	mor_amt_type<br><br>**Show the field ONLY if:**<br>**[mor(1)] = '1'**	**Morphine amount type**<br>*if 10 grams, select "grams."*	**checkbox, Required**<br>1 mor_amt_type_1 milligrams<br>2 mor_amt_type_2 $<br>3 mor_amt_type_3 pills
	733	mor_mg<br><br>**Show the field ONLY if:**<br>**[mor_amt_type(1)] = '1'**	**Maximum milligram amount of morphine documented in the chart**<br>*Enter numerical value. Use decimal value if fraction given.*	**text, Required**
	734	mor_dollar<br><br>**Show the field ONLY if:**<br>**[mor_amt_type(2)] = '1'**	**Maximum dollar amount of morphine documented in the chart**<br>*Enter numerical value.*	**text, Required**
	735	mor_pills<br><br>**Show the field ONLY if:**<br>**[mor_amt_type(3)] = '1'**	**Maximum number of morphine pills documented in the chart**<br>*Enter numerical value.*	**text, Required**
	736	mor_route<br><br>**Show the field ONLY if:**<br>**[mor(2)] = '1'**	**Morphine route of administration**	**checkbox, Required**<br>1 mor_route_1 Injection<br>2 mor_route_2 Snort<br>3 mor_route_3 Smoke<br>4 mor_route_4 Swallow<br>999 mor_route_999 Unknown
	737	mor_freq<br><br>**Show the field ONLY if:**<br>**[mor(3)] = '1'**	**Morphine frequency**	**radio, Required**<br>1 Multiple times per day<br>2 Daily<br>3 Weekly<br>4 Monthly<br>999 Unknown

© 2021 Herring AA et al. *JAMA Network Open.*

	738	**mor_length_mx**<br><br>**Show the field ONLY if: [mor(4)] = '1'**	**Are there discrepant accounts for length of use of morphine documented in the chart?**	**yesno, Required**<br>1 Yes<br>0 No
	739	**mor_length_max**<br><br>**Show the field ONLY if: [mor_length_mx] = '1'**	**Maximum length of use documented**<br>*Enter numerical value.*	**text, Required**
	740	**mor_length_min**<br><br>**Show the field ONLY if: [mor_length_mx] = '1'**	**Minimum length of use documented**<br>*Enter numerical value.*	**text, Required**
	741	**mor_length_avg**<br><br>**Show the field ONLY if: [hm_length_mx] = '1'**	**Average length of use**	**calc, Required**<br>**Calculation: mean([mor_length_max] , [mor_length_min])**
	742	**mor_length_1**<br><br>**Show the field ONLY if: [mor_length_mx] = '0'**	**Length of use**<br>*If 5 years, enter "5." Enter "999" if unknown.*	**text, Required**
	743	**mor_length_2**<br><br>**Show the field ONLY if: [mor(4)] = '1'**	**Unit of time**<br>*If 5 years, select "Years."*	**radio, Required**<br>1 Days<br>2 Weeks<br>3 Months<br>4 Years<br>999 Unknown
	744	**mor_age**<br><br>**Show the field ONLY if: [mor(5)] = '1'**	**Age of first use**	**text, Required**
	745	**oth_primary_use**<br><br>**Show the field ONLY if: [primary_opioid] = '7'**	**Section Header:** *Current [primary_opioid_oth] Use*<br>**Is this use of [primary_opioid_oth] prescribed, illicit, or both?**	**radio, Required**<br>1 Prescribed<br>2 Illicit<br>3 Both<br>999 Unknown/Unclear
	746	**oth_primary_amt_type**<br><br>**Show the field ONLY if: [oth_primary(1)] = '1'**	**Amount Type**<br>*if 10 grams, select "grams."*	**checkbox, Required**<br>1 oth_primary_amt_type_1 grams<br>2 oth_primary_amt_type_2 $<br>3 oth_primary_amt_type_3 bags<br>4 oth_primary_amt_type_4 pills<br>5 oth_primary_amt_type_5 milligrams<br>6 oth_primary_amt_type_6 micrograms
	747	**oth_primary_gram**<br><br>**Show the field ONLY if: [oth_primary_amt_type (1)] = '1'**	**Maximum gram amount documented in the chart**<br>*Enter numerical value.*	**text, Required**

© 2021 Herring AA et al. *JAMA Network Open.*

	748	**oth_primary_dollar**<br><br>**Show the field ONLY if: [oth_primary_amt_type (2)] = '1'**	**Maximum dollar amount documented in the chart**<br>*Enter numerical value.*	**text, Required**
	749	**oth_primary_bag**<br><br>**Show the field ONLY if: [oth_primary_amt_type (3)] = '1'**	**Maximum bag amount documented in the chart**<br>*Enter numerical value.*	**text, Required**
	750	**oth_primary_pills**<br><br>**Show the field ONLY if: [oth_primary_amt_type (4)] = '1'**	**Maximum number of pills documented in the chart**<br>*Enter numerical value.*	**text, Required**
	751	**oth_primary_mg**<br><br>**Show the field ONLY if: [oth_primary_amt_type (5)] = '1'**	**Maximum milligram amount documented in the chart**<br>*Enter numerical value. Use decimal value if fraction given.*	**text, Required**
	752	**oth_primary_mcg**<br><br>**Show the field ONLY if: [oth_primary_amt_type (6)] = '1'**	**Maximum microgram amount documented in the chart**<br>*Enter numerical value. Use decimal value if fraction given.*	**text, Required**
	753	**oth_primary_route**<br><br>**Show the field ONLY if: [oth_primary(2)] = '1'**	**Route of administration**	**checkbox, Required**<br>1 oth_primary_route_1 Injection<br>2 oth_primary_route_2 Snort<br>3 oth_primary_route_3 Smoke<br>4 oth_primary_route_4 Swallow
	754	**oth_primary_freq**<br><br>**Show the field ONLY if: [oth_primary(3)] = '1'**	**Frequency**	**radio, Required**<br>1 Multiple times per day<br>2 Daily<br>3 Weekly<br>4 Monthly<br>999 Unknown
	755	**oth_primary_length_mx**<br><br>**Show the field ONLY if: [oth_primary(4)] = '1'**	**Are there discrepant accounts for length of use of [primary_opioid_oth] documented in the chart?**	**yesno, Required**<br>1 Yes<br>0 No
	756	**oth_primary_length_max**<br><br>**Show the field ONLY if: [oth_primary_length_mx] = '1'**	**Maximum length of use documented**<br>*Enter numerical value.*	**text, Required**
	757	**oth_primary_length_min**<br><br>**Show the field ONLY if: [oth_primary_length_mx] = '1'**	**Minimum length of use documented**<br>*Enter numerical value.*	**text, Required**

© 2021 Herring AA et al. *JAMA Network Open.*

	758	**oth_primary_length_avg**<br><br>**Show the field ONLY if: [oth_primary_length_mx] = '1'**	**Average length of use**	**calc, Required**<br>**Calculation: mean([oth_primary_length_max] , [oth_primary_length_min])**
	759	**oth_primary_length_1**<br><br>**Show the field ONLY if: [oth_primary_length_mx] = '0'**	**Length of use**<br>*If 5 years, enter "5." Enter "999" if unknown.*	**text, Required**
	760	**oth_primary_length_2**<br><br>**Show the field ONLY if: [oth_primary(4)] = '1'**	**Unit of time**<br>*If 5 years, select "Years."*	**radio, Required**<br>1 Days<br>2 Weeks<br>3 Months<br>4 Years<br>999 Unknown
	761	**oth_primary_age**<br><br>**Show the field ONLY if: [oth_primary(5)] = '1'**	**Age of first use**	**text, Required**
	762	**oth_other_use**<br><br>**Show the field ONLY if: [curr_opioid_type(7)] = '1'**	**Section Header:** *Current [curr_opioid_oth] Use*<br>**Is this use of [curr_opioid_oth] prescribed, illicit, or both?**	**radio, Required**<br>1 Prescribed<br>2 Illicit<br>3 Both<br>999 Unknown/Unclear
	763	**oth_other_amt_type**<br><br>**Show the field ONLY if: [oth_other(1)] = '1'**	**Amount Type**<br>*if 10 grams, select "grams."*	**checkbox, Required**<br>1 oth_other_amt_type_1 grams<br>2 oth_other_amt_type_2 $<br>3 oth_other_amt_type_3 bags<br>4 oth_other_amt_type_4 pills<br>5 oth_other_amt_type_5 milligrams<br>6 oth_other_amt_type_6 micrograms
	764	**oth_other_gram**<br><br>**Show the field ONLY if: [oth_other_amt_type(1)] = '1'**	**Maximum gram amount documented in the chart**<br>*Enter numerical value.*	**text, Required**
	765	**oth_other_dollar**<br><br>**Show the field ONLY if: [oth_other_amt_type(2)] = '1'**	**Maximum dollar amount documented in the chart**<br>*Enter numerical value.*	**text, Required**
	766	**oth_other_bag**<br><br>**Show the field ONLY if: [oth_other_amt_type(3)] = '1'**	**Maximum bag amount documented in the chart**<br>*Enter numerical value.*	**text, Required**

© 2021 Herring AA et al. *JAMA Network Open.*

	767	**oth_other_pills**<br><br>**Show the field ONLY if: [oth_other_amt_type(4)] = '1'**	**Maximum number of pills documented in the chart**<br>*Enter numerical value.*	**text, Required**
	768	**oth_other_mg**<br><br>**Show the field ONLY if: [oth_other_amt_type(5)] = '1'**	**Maximum milligram amount documented in the chart**<br>*Enter numerical value. Use decimal value if fraction given.*	**text, Required**
	769	**oth_other_mcg**<br><br>**Show the field ONLY if: [oth_other_amt_type(6)] = '1'**	**Maximum microgram amount documented in the chart**<br>*Enter numerical value. Use decimal value if fraction given.*	**text, Required**
	770	**oth_other_route**<br><br>**Show the field ONLY if: [oth_other(2)] = '1'**	**Route of administration**	**checkbox, Required**<br>1 | oth_other_route_1 | Injection<br>2 | oth_other_route_2 | Snort<br>3 | oth_other_route_3 | Smoke<br>4 | oth_other_route_4 | Swallow
	771	**oth_other_freq**<br><br>**Show the field ONLY if: [oth_other(3)] = '1'**	**Frequency**	**radio, Required**<br>1 | Multiple times per day<br>2 | Daily<br>3 | Weekly<br>4 | Monthly<br>999 | Unknown
	772	**oth_other_length_mx**<br><br>**Show the field ONLY if: [oth_other(4)] = '1'**	**Are there discrepant accounts for length of use of [curr_opioid_oth] documented in the chart?**	**yesno, Required**<br>1 | Yes<br>0 | No
	773	**oth_other_length_max**<br><br>**Show the field ONLY if: [oth_other_length_mx] = '1'**	**Maximum length of use documented**<br>*Enter numerical value.*	**text, Required**
	774	**oth_other_length_min**<br><br>**Show the field ONLY if: [oth_other_length_mx] = '1'**	**Minimum length of use documented**<br>*Enter numerical value.*	**text, Required**
	775	**oth_other_length_avg**<br><br>**Show the field ONLY if: [oth_other_length_mx] = '1'**	**Average length of use**	**calc, Required**<br>**Calculation: mean([oth_other_length_max] , [oth_other_length_min])**
	776	**oth_other_length_1**<br><br>**Show the field ONLY if: [oth_other_length_mx] = '0'**	**Length of use**<br>*If 5 years, enter "5." Enter "999" if unknown.*	**text, Required**

© 2021 Herring AA et al. *JAMA Network Open.*

	777	oth_other_length_2<br><br>Show the field ONLY if: [oth_other(4)] = '1'	**Unit of time**<br>*If 5 years, select "Years."*	**radio, Required**<br>1 Days<br>2 Weeks<br>3 Months<br>4 Years<br>999 Unknown
	778	oth_other_age<br><br>Show the field ONLY if: [oth_other(5)] = '1'	**Age of first use**	**text, Required**
	779	first_opioid_age	**Section Header:** *Substance Use History *****<br>**Age of first opioid exposure?**<br>*Enter "999" if unknown*	**text, Required**
	780	first_opioid_type	**Opioid(s) first used?**<br>*Brand names listed in parantheses*	**checkbox, Required**<br>1 first_opioid_type_1 Oxycodone (OxyContin, Percocet, Roxicodone, etc.)<br>2 first_opioid_type_2 Hydrocodone (Norco, Vicodn etc.)<br>3 first_opioid_type_3 Fentanyl (Duragesic, Ionsys)<br>4 first_opioid_type_4 Hydromorph on (Dilaudid)<br>5 first_opioid_type_5 Methadone<br>6 first_opioid_type_6 Buprenorphine (Suboxone, etc.<br>7 first_opioid_type_7 Morphine<br>8 first_opioid_type_8 Heroin<br>9 first_opioid_type_9 Other<br>999 first_opioid_type_999 Unknown
	781	opioid_type_oth<br><br>Show the field ONLY if: [first_opioid_type(9)] = '1'	**If "Other," please specify.**	**text, Required**

© 2021 Herring AA et al. *JAMA Network Open.*

782	prev_opioid_type	**Previously used opioid(s)?**<br>*Brand names listed in parantheses*	**checkbox, Required**<br>1 prev_opioid_type_1 Oxycodone (OxyContin, Percocet, Roxicodone, etc.)<br>2 prev_opioid_type_2 Hydrocodone (Norco, Vicodin etc.)<br>3 prev_opioid_type_3 Fentanyl (Duragesic, Ionsys)<br>4 prev_opioid_type_4 Hydromorpho (Dilaudid)<br>5 prev_opioid_type_5 Methadone<br>6 prev_opioid_type_6 Buprenorphine (Suboxone, etc<br>7 prev_opioid_type_7 Morphine<br>8 prev_opioid_type_8 Heroin<br>9 prev_opioid_type_9 Other<br>999 prev_opioid_type_999 None of the above
783	prev_opioid_oth<br><br>Show the field ONLY if: [prev_opioid_type(9)] = '1'	**If "Other," please specify.**	**text, Required**
784	preop_sud	**Any documentation of substance use BEFORE opioids?**	**yesno, Required**<br>1 Yes<br>0 No
785	preop_sud_hx<br><br>Show the field ONLY if: [preop_sud] = '1'	**Other substance use (check all that apply)**	**checkbox, Required**<br>1 preop_sud_hx_1 Methamphetamin<br>2 preop_sud_hx_2 Cocaine<br>3 preop_sud_hx_3 Cannabis<br>4 preop_sud_hx_4 Hallucinogens (including Ecstasy/MDMA)<br>5 preop_sud_hx_5 LSD<br>6 preop_sud_hx_6 Benzodiazepines<br>7 preop_sud_hx_7 Alcohol<br>8 preop_sud_hx_8 Tobacco<br>9 preop_sud_hx_9 Other (note any heavy usage)<br>999 preop_sud_hx_999 Unknown/Unclear
786	preop_sud_hx_oth<br><br>Show the field ONLY if: [preop_sud_hx(9)] = '1'	**If "Other," please specify.**	**text, Required**

© 2021 Herring AA et al. *JAMA Network Open.*

	787	**preop_meth_age**<br><br>**Show the field ONLY if: [preop_sud_hx(1)] = '1'**	**Age of methamphetamine exposure**<br>*Enter 999 if unknown.*	**text, Required**
	788	**preop_cocaine_age**<br><br>**Show the field ONLY if: [preop_sud_hx(2)] = '1'**	**Age of cocaine exposure**<br>*Enter 999 if unknown.*	**text, Required**
	789	**preop_mj_age**<br><br>**Show the field ONLY if: [preop_sud_hx(3)] = '1'**	**Age of marijuana exposure**<br>*Enter 999 if unknown.*	**text, Required**
	790	**preop_psych_age**<br><br>**Show the field ONLY if: [preop_sud_hx(4)] = '1'**	**Age of hallucinogen exposure**<br>*Enter 999 if unknown.*	**text, Required**
	791	**preop_lsd_age**<br><br>**Show the field ONLY if: [preop_sud_hx(5)] = '1'**	**Age of LSD exposure**<br>*Enter 999 if unknown.*	**text, Required**
	792	**preop_benzo_age**<br><br>**Show the field ONLY if: [preop_sud_hx(6)] = '1'**	**Age of benzodiazepine exposure**<br>*Enter 999 if unknown.*	**text, Required**
	793	**preop_etoh_age**<br><br>**Show the field ONLY if: [preop_sud_hx(7)] = '1'**	**Age of alcohol exposure**<br>*Enter 999 if unknown.*	**text, Required**
	794	**preop_cigs_age**<br><br>**Show the field ONLY if: [preop_sud_hx(8)] = '1'**	**Age of tobacco exposure**<br>*Enter 999 if unknown.*	**text, Required**
	795	**preop_oth_age**<br><br>**Show the field ONLY if: [preop_sud_hx(9)] = '1'**	**Age of [preop_sud_hx_oth] exposure**<br>*Enter 999 if unknown.*	**text, Required**
	796	**any_chronic_pain**	**Section Header:** *Chronic Pain ****<br><br>**Any documentation of chronic pain?**	**yesno, Required**<br>1 Yes<br>0 No
	797	**chronic_pain_spec**<br><br>**Show the field ONLY if: [any_chronic_pain] = '1'**	**Is the quality or location of chronic pain specified?**	**yesno, Required**<br>1 Yes<br>0 No
	798	**chronic_pain_describe**<br><br>**Show the field ONLY if: [chronic_pain_spec] = '1'**	**Name/describe the location and quality.**	**text, Required**
	799	**cse**	**Section Header:** *Clinically Signiicant Events*<br><br>**Did any clinically significant events occur during this encounter that warrant further review?**<br>*Includes: Clinician-determined precipitated withdrawal, Grade 4 or 5 adverse event*	**yesno**<br>1 Yes<br>0 No
	800	**cse_spec**<br><br>**Show the field ONLY if: [cse] = '1'**	**Which of the following clinically significant events occurred?**	**checkbox**<br>1 cse_spec_1 Precipitated withdrawal (clinician determined)<br>2 cse_spec_2 Life threatening event

	801	attribution_ppt<br><br>Show the field ONLY if:<br>[cse_spec(1)] = '1'	Degree of relatedness to buprenorphine administration (investigator only)	radio<br>1 Unrelated<br>2 Possibly Related<br>3 Probably Related<br>4 Definitely Related
	802	attribution_sae<br><br>Show the field ONLY if:<br>[cse_spec(2)] = '1'	Degree of relatedness to buprenorphine administration (investigator only)	radio<br>1 Unrelated<br>2 Possibly Related<br>3 Probably Related<br>4 Definitely Related
	803	provider_notes_and_ed_buprenorphine_complete	Section Header: *Form Status*<br><br>Complete?	dropdown<br>0 Incomplete<br>1 Unverified<br>2 Complete

Instrument: ED Bridge Intake Form Page 1 **(ed_bridge_intake_form_page_1)**

	804	intake	Section Header: *Intake Form*<br><br>Does the patient have an intake form corresponding to this specific ED visit?	radio, Required<br>1 Yes<br>0 No
	805	any_intake<br><br>Show the field ONLY if:<br>[intake] = '0'	Does the patient have an intake form completed within 30 days after this ED visit?	yesno, Required<br>1 Yes<br>0 No
	806	intake_ed<br><br>Show the field ONLY if:<br>[any_intake] = '1'	Does this intake form belong to another specific ED visit?	yesno, Required<br>1 Yes<br>0 No
	807	ref_md<br><br>Show the field ONLY if:<br>[intake] = '1' OR [intake_ed] = '0'	Section Header: *Patient Information and ED Visit*<br><br>Referring ED provider, as listed on intake sheet<br>*Enter "N/A" if none listed. Please enter as: Last name, First name.*	text, Required
	808	if_no_ins<br><br>Show the field ONLY if:<br>([intake] = '1' or [intake_ed] = '0') and [insurance_1(8)] = '1'	If the patient does not have insurance, was a plan made for obtaining insurance?<br>*See intake form for plan/expected status.*	radio, Required<br>1 Yes<br>0 No<br>999 N/A or not specified on ED Bridge intake form
	809	primary_care<br><br>Show the field ONLY if:<br>[intake] = '1' OR [intake_ed] = '0'	Does the patient have a primary care home?<br>*From intake form.*	radio, Required<br>1 Yes<br>0 No<br>999 Not specified on ED Bridge intake form

	810	mental_health<br><br>Show the field ONLY if:<br>[intake] = '1' OR [intake_ed] = '0'	Section Header: *Other Concerns*<br><br>**Does the patient have a mental health provider?**<br>*From intake sheet.*	radio, Required<br>1 Yes<br>0 No<br>999 Not specified on ED Bridge intake form
	811	trauma<br><br>Show the field ONLY if:<br>[intake] = '1' OR [intake_ed] = '0'	**Were any traumatic events documented on the ED Bridge intake form?**<br>*From intake sheet. Can include physical, emotional, psychological, or other trauma.*	yesno, Required<br>1 Yes<br>0 No
	812	trauma_spec<br><br>Show the field ONLY if:<br>[trauma] = '1'	**Please specify the traumatic event(s).**<br>*E.g. , death of loved one, car accident, etc.*	text, Required
	813	housing<br><br>Show the field ONLY if:<br>[intake] = '1' OR [intake_ed] = '0'	**Current housing situation**	checkbox, Required<br>1 housing_1 Stably housed (own or rent house or apartment, shared housing, etc.)<br>2 housing_2 Marginally/unstably housed (including couch surfing)<br>3 housing_3 Shelter<br>4 housing_4 Street or vehicle<br>5 housing_5 Sobering center (short term, e.g., Cherry Hill)<br>6 housing_6 Sober living facility/halfway house (Teen Challenge, Orchid House, etc.)<br>7 housing_7 Homeless NOS<br>8 housing_8 Other<br>999 housing_999 Unknown
	814	housing_oth<br><br>Show the field ONLY if:<br>[housing(8)] = '1'	**If "Other," please specify.**	text, Required
	815	housing_plan<br><br>Show the field ONLY if:<br>[intake] = '1' OR [intake_ed] = '0'	**If the patient does not currently have housing, was a plan made to obtain housing?**	radio, Required<br>1 Yes<br>0 No<br>999 N/A or not specified on ED Bridge intake form
	816	ed_bridge_intake_form_page_1_complete	Section Header: *Form Status*<br><br>**Complete?**	dropdown<br>0 Incomplete<br>1 Unverified<br>2 Complete

Instrument: ED Bridge Intake Form Page 2 **(ed_bridge_intake_form_page_2)**

	817	intake_2<br>Show the field ONLY if: [intake] = '1' OR [intake_ed] = '0'	Was the second page of the ED Bridge intake form filled out?	yesno, Required<br>1 Yes<br>0 No
	818	psychosocial<br>Show the field ONLY if: [intake_2] = '1'	Section Header: *Psychosocial*<br>Was a psychosocial support person contacted during the ED visit?	radio, Required<br>1 Yes<br>999 Not specified on ED Bridge intake form.
	819	psychosocial_plan<br>Show the field ONLY if: [intake_2] = '1'	Is there a plan for ongoing psychosocial recovery support?	radio, Required<br>1 Yes<br>999 Not specified on ED Bridge intake form.
	820	psychosocial_referral<br>Show the field ONLY if: [intake_2] = '1'	Was an appointment made for psychosocial support?	radio, Required<br>1 Yes<br>999 Not specified on ED Bridge intake form.
	821	mental<br>Show the field ONLY if: [intake_2] = '1'	Section Header: *Mental Health Needs*<br>Any mental health concerns?	radio, Required<br>1 Yes<br>999 Not specified on ED Bridge intake form.
	822	mental_health_ref<br>Show the field ONLY if: [intake_2] = '1'	Was a mental health provider contacted?<br>*From intake sheet.*	radio, Required<br>1 Yes<br>999 Not specified on ED Bridge intake form
	823	mh_plan<br>Show the field ONLY if: [intake_2] = '1'	Is there a psychiatric medication plan?	radio, Required<br>1 Yes<br>999 Not specified on ED Bridge intake form
	824	mh_appt<br>Show the field ONLY if: [intake_2] = '1'	Was an appointment made for mental health concerns?	radio, Required<br>1 Yes<br>999 Not specified on ED Bridge intake form
	825	dv<br>Show the field ONLY if: [intake_2] = '1'	Section Header: *Social*<br>Was the patient screened for domestic violence (DV)?	radio, Required<br>1 Yes<br>999 Not specified on ED Bridge intake form.
	826	dv_notify<br>Show the field ONLY if: [dv] = '1'	If DV confirmed, provider notified?	radio, Required<br>1 Yes<br>999 Not specified on ED Bridge intake form.

	#	Variable / Field Name	Field Label	Field Attributes
	827	social_plan<br><br>Show the field ONLY if: [intake_2] = '1'	Is there a plan for social support?	radio, Required<br>1 Yes<br>999 Not specified on ED Bridge intake form.
	828	mat_plan<br><br>Show the field ONLY if: [intake_2] = '1'	Section Header: *Substance Use Treatment*<br><br>What is the plan for treatment?	checkbox, Required<br>1 mat_plan_1 ED Bridge<br>2 mat_plan_2 BIC<br>3 mat_plan_3 K6/K7<br>4 mat_plan_4 Outside PCP<br>5 mat_plan_5 Out-of-county MAT<br>6 mat_plan_6 Methadone clinic<br>999 mat_plan_999 Not specified on the intake form
	829	ed_bridge_intake_form_page_2_complete	Section Header: *Form Status*<br><br>Complete?	dropdown<br>0 Incomplete<br>1 Unverified<br>2 Complete

Instrument: Follow-Up (followup)

	#	Variable / Field Name	Field Label	Field Attributes
	830	any_contact<br><br>Show the field ONLY if: [intake] = '1' OR [intake_ed] = '0'	Section Header: *Follow-up information provided by MAT coordinator (Christian Hailozian)*<br><br>Was any contact made with the patient within 30 days of their initial ED visit?	radio, Required<br>1 Yes (in-person)<br>2 Yes (phone/text)<br>3 Yes (both)<br>0 No
	831	ed_bridge<br><br>Show the field ONLY if: [intake] = '1' OR [intake_ed] = '0'	Did the patient accept an ED bridge follow-up appointment?	radio, Required<br>1 Yes<br>0 No
	832	engaged<br><br>Show the field ONLY if: [intake] = '1' OR [intake_ed] = '0'	Did the patient attend any healthcare appointments for their opioid use disorder within 30 days of their initial ED visit?	radio, Required<br>1 Yes<br>0 No
	833	outcome<br><br>Show the field ONLY if: [intake] = '1' OR [intake_ed] = '0'	What was this patient's outcome after one month?	checkbox, Required<br>1 outcome_1 Lost to follow-up<br>2 outcome_2 Still in SUD clinic<br>3 outcome_3 Transferred to BIC<br>4 outcome_4 Transferred to primary care<br>5 outcome_5 Transferred to out-of-county MAT<br>6 outcome_6 Transferred to methadone clinic<br>7 outcome_7 Other

	834	**outcome_other**<br><br>**Show the field ONLY if: [outcome(7)] = '1'**	**If "Other," please specify**	**text, Required**
	835	**followup_complete**	**Section Header:** *Form Status*<br><br>**Complete?**	**dropdown**<br>0 Incomplete<br>1 Unverified<br>2 Complete
**Instrument:** Admission Discharge Summary **(admission_discharge_summary)**				
	836	**admit_sedation**<br><br>**Show the field ONLY if: [disp] = '2'**	**Section Header:** *Admit Sedation*<br><br>**Which of the following signs or symptoms of sedation were documented in the discharge summary notes?**	**checkbox, Required**<br>1 admit_sedation_1 Nodding<br>2 admit_sedation_2 Sleepy, drowsy, diffculty staying awake<br>3 admit_sedation_3 Feelings of general fatigue or weakness<br>0 admit_sedation_0 None of the above
	837	**admit_intox**<br><br>**Show the field ONLY if: [disp] = '2'**	**Section Header:** *Admit Intoxication*<br><br>**Which of the following signs or symptoms of intoxication were documented in the discharge summary notes?**	**checkbox, Required**<br>1 admit_intox_1 Good mood<br>2 admit_intox_2 Coasting (Spaced out)<br>3 admit_intox_3 Pleasant Sick<br>4 admit_intox_4 Drive (energy)<br>5 admit_intox_5 High<br>6 admit_intox_6 Drunken<br>7 admit_intox_7 Soapbox (talkative)<br>8 admit_intox_8 Rush<br>9 admit_intox_9 Friendly<br>10 admit_intox_10 Relaxed<br>11 admit_intox_11 Diffculty concentrating<br>12 admit_intox_12 Feeling lightheaded or dizzy<br>13 admit_intox_13 Feeling confused<br>0 admit_intox_0 None of the above

	**838**	**admit_gi**<br><br>**Show the field ONLY if:**<br>**[disp] = '2'**	**Section Header:** *Admit GI Symptoms*<br><br>**Which of the following gastrointestinal signs or symptoms were documented in the discharge summary notes?**	**checkbox, Required**<br>1 | admit_gi_1 | Constipation<br>2 | admit_gi_2 | Turning of stomach<br>3 | admit_gi_3 | Nausea<br>4 | admit_gi_4 | Vomiting<br>5 | admit_gi_5 | Diarrhea<br>0 | admit_gi_0 | None of the above
	**839**	**admit_anxious**<br><br>**Show the field ONLY if:**<br>**[disp] = '2'**	**Section Header:** *Admit Anxiety*<br><br>**Which of the following signs or symptoms of anxiety were documented in the discharge summary notes?**	**checkbox, Required**<br>1 | admit_anxious_1 | Anxiety, irritability, or agitation<br>2 | admit_anxious_2 | Nervousness<br>3 | admit_anxious_3 | Tremor<br>4 | admit_anxious_4 | Restlessness<br>0 | admit_anxious_0 | None of the above
	**840**	**admit_withdrawal**<br><br>**Show the field ONLY if:**<br>**[disp] = '2'**	**Section Header:** *Admit Withdrawal*<br><br>**Which of the following signs or symptoms of withdrawal were documented in the discharge summary notes?**	**checkbox, Required**<br>1 | admit_withdrawal 1 | Yawning<br>2 | admit_withdrawal 2 | Gooseflesh skin (chills/piloerection)<br>3 | admit_withdrawal 3 | Pulse >80<br>4 | admit_withdrawal 4 | Sweating (diaphoresis)<br>5 | admit_withdrawal 5 | Pupil size largerth normal (dilation/mydriasis<br>6 | admit_withdrawal 6 | Bone, joint aches, o body aches (arthralgias/myalgi<br>7 | admit_withdrawal 7 | Runny nose or tearing (rhinorrea lacrimation)<br>0 | admit_withdrawal 0 | None of the above
	**841**	**admit_vitals**<br><br>**Show the field ONLY if:**<br>**[disp] = '2'**	**Section Header:** *Admit Abnormal Vitals*<br><br>**Which of the following abnormal vital signs were documented in the discharge summary notes?**	**checkbox, Required**<br>1 | admit_vitals_1 | Hypoxia<br>2 | admit_vitals_2 | Hypercarbia<br>3 | admit_vitals_3 | Hypotension<br>4 | admit_vitals_4 | Bradycardia<br>0 | admit_vitals_0 | None of the above

	842	admit_misc_sx<br><br>Show the field ONLY if: [disp] = '2'	**Section Header:** *Admit Miscellaneous Opioid-Related Side Effects*<br><br>Which of the following miscellaneous opioid related side effects were documented in the discharge summary notes?	checkbox, Required<br>1 admit_misc_sx_1 Diffculty passing urine<br>2 admit_misc_sx_2 Itchiness<br>3 admit_misc_sx_3 Dry mouth<br>4 admit_misc_sx_4 Headache<br>5 admit_misc_sx_5 Unable to walk<br>6 admit_misc_sx_6 Unable to discharge<br>7 admit_misc_sx_7 Pupil size smaller than normal (constriction/miosis)<br>0 admit_misc_sx_0 None of the above
	843	admit_death<br><br>Show the field ONLY if: [disp] = '2'	**Section Header:** *Admit Death*<br><br>Did the discharge summary notes indicate that the patient died?	yesno, Required<br>1 Yes<br>0 No
	844	admission_discharge_summary_complete	**Section Header:** *Form Status*<br><br>Complete?	dropdown<br>0 Incomplete<br>1 Unverified<br>2 Complete
**Instrument:** Pre-Bup Chart Scoring **(prebup_chart_scoring)**				
	845	agonist_1_pre	**Section Header:** *Opiate 32 (16 Agonist)*<br><br>Nodding	calc<br>Calculation: if([prebup_sedation(1)] = 1, 1, 0)
	846	agonist_2_pre	Sleepy	calc<br>Calculation: if([prebup_sedation(2)] = 1, 1, 0)
	847	agonist_3_pre	Good mood	calc<br>Calculation: if([prebup_intox(1)] = 1, 1, 0)
	848	agonist_4_pre	Coasting	calc<br>Calculation: if([prebup_intox(2)] = 1, 1, 0)
	849	agonist_5_pre	Pleasant Sick	calc<br>Calculation: if([prebup_intox(3)] = 1, 1, 0)
	850	agonist_6_pre	Drive	calc<br>Calculation: if([prebup_intox(4)] = 1, 1, 0)
	851	agonist_7_pre	High	calc<br>Calculation: if([prebup_intox(5)] = 1, 1, 0)
	852	agonist_8_pre	Drunken	calc<br>Calculation: if([prebup_intox(6)] = 1, 1, 0)
	853	agonist_9_pre	Soapbox	calc<br>Calculation: if([prebup_intox(7)] = 1, 1, 0)
	854	agonist_10_pre	Rush	calc<br>Calculation: if([prebup_intox(8)] = 1, 1, 0)
	855	agonist_11_pre	Friendly	calc<br>Calculation: if([prebup_intox(9)] = 1, 1, 0)
	856	agonist_12_pre	Relaxed	calc<br>Calculation: if([prebup_intox(10)] = 1, 1, 0)

	857	agonist_13_pre	Turning of stomach	calc<br>Calculation: if([prebup_gi(2)] = 1, 1, 0)
	858	agonist_14_pre	Nervousness	calc<br>Calculation: if([prebup_anxious(2)] = 1, 1, 0)
	859	agonist_15_pre	Itchy	calc<br>Calculation: if([prebup_misc_sx(2)] = 1, 1, 0)
	860	agonist_16_pre	Dry mouth	calc<br>Calculation: if([prebup_misc_sx(3)] = 1, 1, 0)
	861	sum_agonist_pre	Sum Agonist 16	calc<br>Calculation: sum([agonist_1_pre], [agonist_2_pre], [agonist_3_pre], [agonist_4_pre], [agonist_5_pre], [agonist_6_pre], [agonist_7_pre], [agonist_8_pre], [agonist_9_pre], [agonist_10_pre], [agonist_11_pre], [agonist_12_pre], [agonist_13_pre], [agonist_14_pre], [agonist_15_pre], [agonist_16_pre])
	862	antagonist_1_pre	**Section Header:** *Opiate 32 (Antagonist 9)*<br>Yawning	calc<br>Calculation: if([prebup_withdrawal(1)] = 1, 1, 0)
	863	antagonist_2_pre	Restlessness	calc<br>Calculation: if([prebup_anxious(4)] = 1, 1, 0)
	864	antagonist_3_pre	Tearing or Runny Nose	calc<br>Calculation: if([prebup_withdrawal(7)] = 1, 1, 0)
	865	antagonist_4_pre	Turning of stomach/cramping/abdominal pain	calc<br>Calculation: if([prebup_gi(2)] = 1, 1, 0)
	866	antagonist_5_pre	Anxiety, irritability, or agitation	calc<br>Calculation: if([prebup_anxious(1)] = 1, 1, 0)
	867	antagonist_6_pre	Chills or gooseflesh skin	calc<br>Calculation: if([prebup_withdrawal(2)] = 1, 1, 0)
	868	antagonist_7_pre	Sweating	calc<br>Calculation: if([prebup_withdrawal(4)] = 1, 1, 0)
	869	antagonist_8_pre	Bone, joint, or body aches	calc<br>Calculation: if([prebup_withdrawal(6)] = 1, 1, 0)
	870	antagonist_9_pre	Feelings of general fatigue or weakness	calc<br>Calculation: if([prebup_sedation(3)] = 1, 1, 0)
	871	sum_antagonist_pre	Sum antagonist 9	calc<br>Calculation: sum([antagonist_1_pre], [antagonist_2_pre], [antagonist_3_pre], [antagonist_4_pre], [antagonist_5_pre], [antagonist_6_pre], [antagonist_7_pre], [antagonist_8_pre], [antagonist_9_pre])
	872	orsds_1_pre	**Section Header:** *OR-SDS*<br>Nausea	calc<br>Calculation: if([prebup_gi(3)] = 1, 1, 0)
	873	orsds_2_pre	Vomiting	calc<br>Calculation: if([prebup_gi(4)] = 1, 1, 0)

	874	orsds_3_pre	Constipation	calc<br>Calculation: if([prebup_gi(1)] = 1, 1, 0)
	875	orsds_4_pre	Diffculty passing urine	calc<br>Calculation: if([prebup_misc_sx(1)] = 1, 1, 0)
	876	orsds_5_pre	Diffculty concentrating	calc<br>Calculation: if([prebup_intox(11)] = 1, 1, 0)
	877	orsds_6_pre	Drowsiness/diffculty staying awake	calc<br>Calculation: if([prebup_sedation(2)] = 1, 1, 0)
	878	orsds_7_pre	Feeling lightheaded or dizzy	calc<br>Calculation: if([prebup_intox(12)] = 1, 1, 0)
	879	orsds_8_pre	Feeling confused	calc<br>Calculation: if([prebup_intox(13)] = 1, 1, 0)
	880	orsds_9_pre	Feelings of general fatigue or weakness	calc<br>Calculation: if([prebup_sedation(3)] = 1, 1, 0)
	881	orsds_10_pre	Itchiness	calc<br>Calculation: if([prebup_misc_sx(2)] = 1, 1, 0)
	882	orsds_11_pre	Dry mouth	calc<br>Calculation: if([prebup_misc_sx(3)] = 1, 1, 0)
	883	orsds_12_pre	Headache	calc<br>Calculation: if([prebup_misc_sx(4)] = 1, 1, 0)
	884	sum_orsds_pre	Sum OR-SDS	calc<br>Calculation: sum([orsds_1_pre], [orsds_2_pre], [orsds_3_pre], [orsds_4_pre], [orsds_5_pre], [orsds_6_pre], [orsds_7_pre], [orsds_8_pre], [orsds_9_pre], [orsds_10_pre], [orsds_11_pre], [orsds_12_pre])
	885	cows_1_pre	Section Header: *COWS*<br>Pulse > 80	calc<br>Calculation: if([prebup_withdrawal(3)] = 1, 1, 0)
	886	cows_2_pre	Sweating	calc<br>Calculation: if([prebup_withdrawal(4)] = 1, 1, 0)
	887	cows_3_pre	Restlessness	calc<br>Calculation: if([prebup_anxious(4)] = 1, 1, 0)
	888	cows_4_pre	Pupil size larger than normal	calc<br>Calculation: if([prebup_withdrawal(5)] = 1, 1, 0)
	889	cows_5_pre	Bone, joint, or body aches	calc<br>Calculation: if([prebup_withdrawal(6)] = 1, 1, 0)
	890	cows_6_pre	GI Upset - stomach cramps, nausea, vomiting, diarrhea	calc<br>Calculation: (if([prebup_gi(2)] = 1, 1, 0)) or (if([prebup_gi(3)] = 1, 1, 0)) or (if([prebup_gi(4)] = 1, 1, 0)) or (if([prebup_gi(5)] = 1, 1, 0))
	891	cows_7_pre	Tremor	calc<br>Calculation: if([prebup_anxious(3)] = 1, 1, 0)
	892	cows_8_pre	Yawning	calc<br>Calculation: if([prebup_withdrawal(1)] = 1, 1, 0)

© 2021 Herring AA et al. *JAMA Network Open.*

	893	cows_9_pre	Anxiety, irritability, or agitation	calc<br>Calculation: if([prebup_anxious(1)] = 1, 1, 0)
	894	cows_10_pre	Gooseflesh skin	calc<br>Calculation: if([prebup_withdrawal(2)] = 1, 1, 0)
	895	sum_cows_pre	Sum COWS	calc<br>Calculation: sum([cows_1_pre], [cows_2_pre], [cows_3_pre], [cows_4_pre], [cows_5_pre], [cows_6_pre], [cows_7_pre], [cows_8_pre], [cows_9_pre], [cows_10_pre])
	896	prebup_chart_scoring_complete	Section Header: *Form Status*<br>Complete?	dropdown<br>0 Incomplete<br>1 Unverified<br>2 Complete
**Instrument: Post-Bup Chart Scoring (postbup_chart_scoring)**				
	897	agonist_1_post	Section Header: *Opiate 32 (16 Agonist)*<br>Nodding	calc<br>Calculation: if([postbup_sedation(1)] = 1, 1, 0)
	898	agonist_2_post	Sleepy	calc<br>Calculation: if([postbup_sedation(2)] = 1, 1, 0)
	899	agonist_3_post	Good mood	calc<br>Calculation: if([postbup_intox(1)] = 1, 1, 0)
	900	agonist_4_post	Coasting	calc<br>Calculation: if([postbup_intox(2)] = 1, 1, 0)
	901	agonist_5_post	Pleasant Sick	calc<br>Calculation: if([postbup_intox(3)] = 1, 1, 0)
	902	agonist_6_post	Drive	calc<br>Calculation: if([postbup_intox(4)] = 1, 1, 0)
	903	agonist_7_post	High	calc<br>Calculation: if([postbup_intox(5)] = 1, 1, 0)
	904	agonist_8_post	Drunken	calc<br>Calculation: if([postbup_intox(6)] = 1, 1, 0)
	905	agonist_9_post	Soapbox	calc<br>Calculation: if([postbup_intox(7)] = 1, 1, 0)
	906	agonist_10_post	Rush	calc<br>Calculation: if([postbup_intox(8)] = 1, 1, 0)
	907	agonist_11_post	Friendly	calc<br>Calculation: if([postbup_intox(9)] = 1, 1, 0)
	908	agonist_12_post	Relaxed	calc<br>Calculation: if([postbup_intox(10)] = 1, 1, 0)
	909	agonist_13_post	Turning of stomach	calc<br>Calculation: if([postbup_gi(2)] = 1, 1, 0)
	910	agonist_14_post	Nervousness	calc<br>Calculation: if([postbup_anxious(2)] = 1, 1, 0)
	911	agonist_15_post	Itchy	calc<br>Calculation: if([postbup_misc_sx(2)] = 1, 1, 0)

	912	agonist_16_post	Dry mouth	calc<br>Calculation: if([postbup_misc_sx(3)] = 1, 1, 0)
	913	sum_agonist_post	Sum Agonist 16	calc<br>Calculation: sum([agonist_1_post], [agonist_2_post], [agonist_3_post], [agonist_4_post], [agonist_5_post], [agonist_6_post], [agonist_7_post], [agonist_8_post], [agonist_9_post], [agonist_10_post], [agonist_11_post], [agonist_12_post], [agonist_13_post], [agonist_14_post], [agonist_15_post], [agonist_16_post])
	914	antagonist_1_post	**Section Header:** *Opiate 32 (Antagonist 9)*<br>Yawning	calc<br>Calculation: if([postbup_withdrawal(1)] = 1, 1, 0)
	915	antagonist_2_post	Restlessness	calc<br>Calculation: if([postbup_anxious(4)] = 1, 1, 0)
	916	antagonist_3_post	Tearing or Runny Nose	calc<br>Calculation: if([postbup_withdrawal(7)] = 1, 1, 0)
	917	antagonist_4_post	Turning of stomach/cramping/abdominal pain	calc<br>Calculation: if([postbup_gi(2)] = 1, 1, 0)
	918	antagonist_5_post	Anxiety, irritability, or agitation	calc<br>Calculation: if([postbup_anxious(1)] = 1, 1, 0)
	919	antagonist_6_post	Chills or gooseflesh skin	calc<br>Calculation: if([postbup_withdrawal(2)] = 1, 1, 0)
	920	antagonist_7_post	Sweating	calc<br>Calculation: if([postbup_withdrawal(4)] = 1, 1, 0)
	921	antagonist_8_post	Bone, joint, or body aches	calc<br>Calculation: if([postbup_withdrawal(6)] = 1, 1, 0)
	922	antagonist_9_post	Feelings of general fatigue or weakness	calc<br>Calculation: if([postbup_sedation(3)] = 1, 1, 0)
	923	sum_antagonist_post	Sum antagonist 9	calc<br>Calculation: sum([antagonist_1_post], [antagonist_2_post], [antagonist_3_post], [antagonist_4_post], [antagonist_5_post], [antagonist_6_post], [antagonist_7_post], [antagonist_8_post], [antagonist_9_post])
	924	orsds_1_post	**Section Header:** *OR-SDS*<br>Nausea	calc<br>Calculation: if([postbup_gi(3)] = 1, 1, 0)
	925	orsds_2_post	Vomiting	calc<br>Calculation: if([postbup_gi(4)] = 1, 1, 0)
	926	orsds_3_post	Constipation	calc<br>Calculation: if([postbup_gi(1)] = 1, 1, 0)
	927	orsds_4_post	Diffculty passing urine	calc<br>Calculation: if([postbup_misc_sx(1)] = 1, 1, 0)
	928	orsds_5_post	Diffculty concentrating	calc<br>Calculation: if([postbup_intox(11)] = 1, 1, 0)

	929	orsds_6_post	Drowsiness/diffculty staying awake	calc<br>Calculation: if([postbup_sedation(2)] = 1, 1, 0)
	930	orsds_7_post	Feeling lightheaded or dizzy	calc<br>Calculation: if([postbup_intox(12)] = 1, 1, 0)
	931	orsds_8_post	Feeling confused	calc<br>Calculation: if([postbup_intox(13)] = 1, 1, 0)
	932	orsds_9_post	Feelings of general fatigue or weakness	calc<br>Calculation: if([postbup_sedation(3)] = 1, 1, 0)
	933	orsds_10_post	Itchiness	calc<br>Calculation: if([postbup_misc_sx(2)] = 1, 1, 0)
	934	orsds_11_post	Dry mouth	calc<br>Calculation: if([postbup_misc_sx(3)] = 1, 1, 0)
	935	orsds_12_post	Headache	calc<br>Calculation: if([postbup_misc_sx(4)] = 1, 1, 0)
	936	sum_orsds_post	Sum OR-SDS	calc<br>Calculation: sum([orsds_1_post], [orsds_2_post], [orsds_3_post], [orsds_4_post], [orsds_5_post], [orsds_6_post], [orsds_7_post], [orsds_8_post], [orsds_9_post], [orsds_10_post], [orsds_11_post], [orsds_12_post])
	937	cows_1_post	Section Header: *COWS*<br>Pulse > 80	calc<br>Calculation: if([postbup_withdrawal(3)] = 1, 1, 0)
	938	cows_2_post	Sweating	calc<br>Calculation: if([postbup_withdrawal(4)] = 1, 1, 0)
	939	cows_3_post	Restlessness	calc<br>Calculation: if([postbup_anxious(4)] = 1, 1, 0)
	940	cows_4_post	Pupil size larger than normal	calc<br>Calculation: if([postbup_withdrawal(5)] = 1, 1, 0)
	941	cows_5_post	Bone, joint, or body aches	calc<br>Calculation: if([postbup_withdrawal(6)] = 1, 1, 0)
	942	cows_6_post	GI Upset - stomach cramps, nausea, vomiting, diarrhea	calc<br>Calculation: if([postbup_gi(2)] = 1, 1, 0) or if([postbup_gi(3)] = 1, 1, 0) or if([postbup_gi(4)] = 1, 1, 0) or if([postbup_gi(5)] = 1, 1, 0)
	943	cows_7_post	Tremor	calc<br>Calculation: if([postbup_anxious(3)] = 1, 1, 0)
	944	cows_8_post	Yawning	calc<br>Calculation: if([postbup_withdrawal(1)] = 1, 1, 0)
	945	cows_9_post	Anxiety, irritability, or agitation	calc<br>Calculation: if([postbup_anxious(1)] = 1, 1, 0)

	946	cows_10_post	Gooseflesh skin	calc<br>Calculation: if([postbup_withdrawal(2)] = 1, 1, 0)
	947	sum_cows_post	Sum COWS	calc<br>Calculation: sum([cows_1_post], [cows_2_post], [cows_3_post], [cows_4_post], [cows_5_post], [cows_6_post], [cows_7_post], [cows_8_post], [cows_9_post], [cows_10_post])
	948	admit_post	**Section Header:** *Serious Adverse Events*<br>Hospitalization	calc<br>Calculation: if([disp] = 2, 1, 0)
	949	threat_post	CTCAE Grade 4 (Life-threatening consequences; urgent intervention indicated)	calc<br>Calculation: if([cse_spec(2)] = 1, 1, 0)
	950	death_post	CTCAE Grade 5 (Death from AE)	calc<br>Calculation: if([ed_death] = 1, 1, 0)
	951	sum_sae_post	Sum SAE	calc<br>Calculation: sum([admit_post], [threat_post], [death_post])
	952	postbup_chart_scoring_complete	**Section Header:** *Form Status*<br>Complete?	dropdown<br>0 Incomplete<br>1 Unverified<br>2 Complete
**Instrument:** Return Visit(s) (return_visits)				
	953	return_yn	Did the patient return to the emergency department within 10 days of this visit?	yesno<br>1 Yes<br>0 No
	954	return_num<br>Show the field ONLY if: [return_yn] = '1'	How many times did the patient return within 10 days of this visit?	radio<br>0 0<br>1 1<br>2 2<br>3 3<br>4 4<br>5 5<br>6 6<br>7 7
	955	datetime_return_1<br>Show the field ONLY if: [return_num] = '1' or [return_num] = '2' or [return_num] = '3' or [return_num] = '4' or [return_num] = '5' or [return_num] = '6' or [return_num] = '7'	**Section Header:** *First Return Visit*<br>Date and time of arrival	text (datetime_mdy)

	956	return_redcap_1<br>Show the field ONLY if: [return_num] = '1' or [return_num] = '2' or [return_num] = '3' or [return_num] = '4' or [return_num] = '5' or [return_num] = '6' or [return_num] = '7'	Does this encounter coincide with an existing visit in REDCap?	yesno<br>1 Yes<br>0 No
	957	return_redcap_spec_1<br>Show the field ONLY if: [return_redcap_1] = '1'	Please enter the visit number	text
	958	return_od_1<br>Show the field ONLY if: [return_num] = '1' or [return_num] = '2' or [return_num] = '3' or [return_num] = '4' or [return_num] = '5' or [return_num] = '6' or [return_num] = '7'	Did the patient present to the ED for opioid overdose?	yesno<br>1 Yes<br>0 No
	959	return_substance_yn_1<br>Show the field ONLY if: [return_num] = '1' or [return_num] = '2' or [return_num] = '3' or [return_num] = '4' or [return_num] = '5' or [return_num] = '6' or [return_num] = '7'	Did the patient or provider report any substance use between the index ED visit and this return visit?	yesno<br>1 Yes<br>0 No
	960	return_substance_1<br>Show the field ONLY if: [return_substance_yn_1] = '1'	Which substance(s) did the patient use?	checkbox<br>1 return_substance_1 1 Opioids (not including buprenorph<br>2 return_substance_1 2 Methamphe<br>3 return_substance_1_ 3 Cocaine<br>4 return_substance_1 4 Cannabis<br>5 return_substance_1 5 Hallucinoge (including Ecstasy/MD<br>6 return_substance_1_ 6 LSD<br>7 return_substance_1 7 Benzodiaze<br>8 return_substance_1 8 Alcohol<br>9 return_substance_1_ 9 Tobacco<br>10 return_substance_1_ 10 Other stimu NOS<br>11 return_substance_1_ 11 Other (note heavy usage<br>999 return_substance_1_999 None of the

	961	return_substance_oth_1<br><br>Show the field ONLY if: [return_substance_1(1 1)] = '1'	If "Other," please specify.	text
	962	return_opioid_1<br><br>Show the field ONLY if: [return_substance_1(1)] = '1'	Which opioid(s) did the patient use?	checkbox<br>1 return_opioid_1_1 Heroin<br>2 return_opioid_1_2 Oxycodone (OxyContin, Percocet, etc.)<br>3 return_opioid_1_3 Hydrocodone (Norco, Vicodin, etc.)<br>4 return_opioid_1_4 Fentanyl (Duragesic, Ionsys)<br>5 return_opioid_1_5 Hydromorphone (Dilaudid)<br>6 return_opioid_1_6 Morphine<br>7 return_opioid_1_7 Other
	963	return_opioid_oth_1<br><br>Show the field ONLY if: [return_opioid_1(7)] = '1'	If "Other," please specify.	text
	964	return_utox_1<br><br>Show the field ONLY if: [return_num] = '1' or [re turn_num] = '2' or [retur n_num] = '3' or [return_ num] = '4' or [return_nu m] = '5' or [return_num] = '6' or [return_num] = '7'	Was a urine toxicology screen performed for this visit?	yesno<br>1 Yes<br>0 No
	965	return_utox_spec_1<br><br>Show the field ONLY if: [return_utox_1] = '1'	Please select all drugs shown to be positive in the urine toxicology analysis:	checkbox<br>1 return_utox_spec_1_1 Amphetami<br>2 return_utox_spec_1_2 Barbiturates<br>3 return_utox_spec_1_3 Cocaine<br>4 return_utox_spec_1_4 Methaqualo<br>5 return_utox_spec_1_5 Methadone<br>6 return_utox_spec_1_6 Methamphe<br>7 return_utox_spec_1_7 Opiates<br>8 return_utox_spec_1_8 Phencyclidin<br>9 return_utox_spec_1_9 Marijuana<br>10 return_utox_spec_1_10 Benzodiazep<br>11 return_utox_spec_1_11 Propoxyphe<br>12 return_utox_spec_1_12 Amphetami<br>13 return_utox_spec_1_13 Other<br>999 return_utox_spec_1 999 None of the

	966	**return_utox_oth_1**<br><br>**Show the field ONLY if: [return_utox_spec_1(1 3)] = '1'**	**If "Other," please specify**	**text**
	967	**return_craving_1**<br><br>**Show the field ONLY if: [return_num] = '1' or [return_num] = '2' or [return_num] = '3' or [return_num] = '4' or [return_num] = '5' or [return_num] = '6' or [return_num] = '7'**	**Did the patient or provider mention opioid cravings during the visit?**	**yesno**<br>1 Yes<br>0 No
	968	**return_wd_1**<br><br>**Show the field ONLY if: [return_num] = '1' or [return_num] = '2' or [return_num] = '3' or [return_num] = '4' or [return_num] = '5' or [return_num] = '6' or [return_num] = '7'**	**Did the patient complaint about or exhibit any signs or symptoms of withdrawal during this visit?**<br>*If the patient received buprenorphine during this visit, only select signs/symptoms documented BEFORE buprenorphine administration.*	**yesno**<br>1 Yes<br>0 No
	969	**return_sedation_1**<br><br>**Show the field ONLY if: [return_wd_1] = '1'**	**Section Header:** *Return Sedation 1*<br><br>**Which of the following signs or symptoms of sedation were documented?**	**checkbox**<br>1 return_sedation_1_1 Nodding<br>2 return_sedation_1_2 Sleepy, drowsy, diffculty staying awake<br>3 return_sedation_1_3 Feelings of general fatigue or weakness<br>0 return_sedation_1_0 None of the above

	970	**return_intox_1**<br><br>**Show the field ONLY if:**<br>**[return_wd_1] = '1'**	**Section Header:** *Return Intoxication 1*<br><br>**Which of the following signs or symptoms of intoxication were documented?**	**checkbox**<br>1 return_intox_1_1 Good mood<br>2 return_intox_1_2 Coasting (Spaced out)<br>3 return_intox_1_3 Pleasant Sick<br>4 return_intox_1_4 Drive (energy)<br>5 return_intox_1_5 High<br>6 return_intox_1_6 Drunken<br>7 return_intox_1_7 Soapbox (talkative)<br>8 return_intox_1_8 Rush<br>9 return_intox_1_9 Friendly<br>10 return_intox_1_10 Relaxed<br>11 return_intox_1_11 Diffculty concentrating<br>12 return_intox_1_12 Feeling lightheaded or dizzy<br>13 return_intox_1_13 Feeling confused<br>0 return_intox_1_0 None of the above
	971	**return_gi_1**<br><br>**Show the field ONLY if:**<br>**[return_wd_1] = '1'**	**Section Header:** *Return GI 1*<br><br>**Which of the following gastrointestinal signs or symptoms were documented?**	**checkbox**<br>1 return_gi_1_1 Constipation<br>2 return_gi_1_2 Turning of stomach/cramping/abdo pain<br>3 return_gi_1_3 Nausea<br>4 return_gi_1_4 Vomiting<br>5 return_gi_1_5 Diarrhea<br>0 return_gi_1_0 None of the above
	972	**return_anxiety_1**<br><br>**Show the field ONLY if:**<br>**[return_wd_1] = '1'**	**Section Header:** *Return Anxiety 1*<br><br>**Which of the following signs or symptoms of anxiety were documented?**	**checkbox**<br>1 return_anxiety_1_1 Anxiety, irritability, or agitation<br>2 return_anxiety_1_2 Nervousness<br>3 return_anxiety_1_3 Tremor<br>4 return_anxiety_1_4 Restlessness<br>0 return_anxiety_1_0 None of the above

	973	**return_withdrawal_1**<br><br>**Show the field ONLY if:**<br>**[return_wd_1] = '1'**	**Section Header:** *Return Withdrawal 1*<br><br>**Which of the following signs or symptoms of withdrawal were documented?**	**checkbox**<br>1 return_withdrawal_1_1 Yawning<br>2 return_withdrawal_1_2 Gooseflesh skin (chills/piloerecti<br>3 return_withdrawal_1_3 Pulse > 80<br>4 return_withdrawal_1_4 Sweating (diaphoresis)<br>5 return_withdrawal_1_5 Pupil size larger normal (dilation/mydria<br>6 return_withdrawal_1_6 Bone, joint ache body aches (arthralgias/mya<br>7 return_withdrawal_1_7 Runny nose or tearing (rhino or lacrimation)<br>0 return_withdrawal_1_0 None of the abo
	974	**return_vitals_1**<br><br>**Show the field ONLY if:**<br>**[return_wd_1] = '1'**	**Section Header:** *Return Abnormal Vitals 1*<br><br>**Which of the following abnormal vital signs were documented?**	**checkbox**<br>1 return_vitals_1_1 Hypoxia<br>2 return_vitals_1_2 Hypercarbia<br>3 return_vitals_1_3 Hypotension<br>4 return_vitals_1_4 Bradycardia<br>0 return_vitals_1_0 None of the above
	975	**return_misc_1**<br><br>**Show the field ONLY if:**<br>**[return_wd_1] = '1'**	**Section Header:** *Return Miscellaneous 1*<br><br>**Which of the following miscellaneous opioid related side effects were documented?**	**checkbox**<br>1 return_misc_1_1 Diffculty passing urine<br>2 return_misc_1_2 Itchiness<br>3 return_misc_1_3 Dry mouth<br>4 return_misc_1_4 Headache<br>5 return_misc_1_5 Unable to walk<br>6 return_misc_1_6 Unable to discharge<br>7 return_misc_1_7 Pupil size smaller than normal (constriction/miosis)<br>0 return_misc_1_0 None of the above
	976	**datetime_return_2**<br><br>**Show the field ONLY if:**<br>**[return_num] = '2' or [return_num] = '3' or [return_num] = '4' or [return_num] = '5' or [return_num] = '6' or [return_num] = '7'**	**Section Header:** *Second Return Visit*<br><br>**Date and time of arrival**	**text (datetime_mdy)**

	977	return_redcap_2<br><br>Show the field ONLY if: [return_num] = '2' or [return_num] = '3' or [return_num] = '4' or [return_num] = '5' or [return_num] = '6' or [return_num] = '7'	Does this encounter coincide with an existing visit in REDCap?	yesno<br>1 Yes<br>0 No
	978	return_redcap_spec_2<br><br>Show the field ONLY if: [return_redcap_2] = '1'	Please enter the visit number	text
	979	return_od_2<br><br>Show the field ONLY if: [return_num] = '2' or [return_num] = '3' or [return_num] = '4' or [return_num] = '5' or [return_num] = '6' or [return_num] = '7'	Did the patient present to the ED for opioid overdose?	yesno<br>1 Yes<br>0 No
	980	return_substance_yn_2<br><br>Show the field ONLY if: [return_num] = '2' or [return_num] = '3' or [return_num] = '4' or [return_num] = '5' or [return_num] = '6' or [return_num] = '7'	Did the patient or provider report any<br><br>substance use between the index ED visit and this return visit?	yesno<br>1 Yes<br>0 No
	981	return_substance_2<br><br>Show the field ONLY if: [return_substance_yn_2] = '1'	Which substance(s) did the patient use?	checkbox<br>1 return_substance_2 1 Opioids (not including buprenorph<br>2 return_substance_2 2 Methamphe<br>3 return_substance_2_ 3 Cocaine<br>4 return_substance_2 4 Cannabis<br>5 return_substance_2 5 Hallucinoge (including Ecstasy/MD<br>6 return_substance_2_ 6 LSD<br>7 return_substance_2 7 Benzodiaze<br>8 return_substance_2 8 Alcohol<br>9 return_substance_2_ 9 Tobacco<br>10 return_substance_2_ 10 Other stimu NOS<br>11 return_substance_2_ 11 Other (note heavy usage<br>999 return_substance_2 999 None of the

	982	return_substance_oth_2<br><br>Show the field ONLY if: [return_substance_2(11)] = '1'	If "Other," please specify.	text
	983	return_opioid_2<br><br>Show the field ONLY if: [return_substance_2(1)] = '1'	Which opioid(s) did the patient use?	checkbox<br>1 return_opioid_2_1 Heroin<br>2 return_opioid_2_2 Oxycodone (OxyContin, Percocet, etc.)<br>3 return_opioid_2_3 Hydrocodone (Norco, Vicodin, etc.)<br>4 return_opioid_2_4 Fentanyl (Duragesic, Ionsys)<br>5 return_opioid_2_5 Hydromorphone (Dilaudid)<br>6 return_opioid_2_6 Morphine<br>7 return_opioid_2_7 Other
	984	return_opioid_oth_2<br><br>Show the field ONLY if: [return_opioid_2(7)] = '1'	If "Other," please specify.	text
	985	return_utox_2<br><br>Show the field ONLY if: [return_num] = '2' or [return_num] = '3' or [return_num] = '4' or [return_num] = '5' or [return_num] = '6' or [return_num] = '7'	Was a urine toxicology screen performed for this visit?	yesno<br>1 Yes<br>0 No
	986	return_utox_spec_2<br><br>Show the field ONLY if: [return_utox_2] = '1'	Please select all drugs shown to be positive in the urine toxicology analysis:	checkbox<br>1 return_utox_spec_2_1 Amphetami<br>2 return_utox_spec_2_2 Barbiturates<br>3 return_utox_spec_2_3 Cocaine<br>4 return_utox_spec_2_4 Methaqualo<br>5 return_utox_spec_2_5 Methadone<br>6 return_utox_spec_2_6 Methamphe<br>7 return_utox_spec_2_7 Opiates<br>8 return_utox_spec_2_8 Phencyclidin<br>9 return_utox_spec_2_9 Marijuana<br>10 return_utox_spec_2_10 Benzodiazep<br>11 return_utox_spec_2_11 Propoxyphe<br>12 return_utox_spec_2_12 Amphetami<br>13 return_utox_spec_2_13 Other<br>999 return_utox_spec_2 999 None of the

	987	**return_utox_oth_2**<br><br>**Show the field ONLY if: [return_utox_spec_2(13)] = '1'**	**If "Other," please specify**	**text**
	988	**return_craving_2**<br><br>**Show the field ONLY if: [return_num] = '2' or [return_num] = '3' or [return_num] = '4' or [return_num] = '5' or [return_num] = '6' or [return_num] = '7'**	**Did the patient or provider mention opioid cravings during the visit?**	**yesno**<br>1 Yes<br>0 No
	989	**return_wd_2**<br><br>**Show the field ONLY if: [return_num] = '2' or [return_num] = '3' or [return_num] = '4' or [return_num] = '5' or [return_num] = '6' or [return_num] = '7'**	**Did the patient complaint about or exhibit any signs or symptoms of withdrawal during this visit?**<br>*If the patient received buprenorphine during this visit, only select signs/symptoms documented BEFORE buprenorphine administration.*	**yesno**<br>1 Yes<br>0 No
	990	**return_sedation_2**<br><br>**Show the field ONLY if: [return_wd_2] = '1'**	**Section Header:** *Return Sedation 2*<br><br>**Which of the following signs or symptoms of sedation were documented?**	**checkbox**<br>1 return_sedation_2_1 Nodding<br>2 return_sedation_2_2 Sleepy, drowsy, diffculty staying awake<br>3 return_sedation_2_3 Feelings of general fatigue or weakness<br>0 return_sedation_2_0 None of the above

	991	**return_intox_2**<br><br>**Show the field ONLY if: [return_wd_2] = '1'**	**Section Header:** *Return Intoxication 2*<br><br>**Which of the following signs or symptoms of intoxication were documented?**	**checkbox**<br>1 **return_intox_2_1** **Good mood**<br>2 **return_intox_2_2** **Coasting (Spaced out)**<br>3 **return_intox_2_3** **Pleasant Sick**<br>4 **return_intox_2_4** **Drive (energy)**<br>5 **return_intox_2_5** **High**<br>6 **return_intox_2_6** **Drunken**<br>7 **return_intox_2_7** **Soapbox (talkative)**<br>8 **return_intox_2_8** **Rush**<br>9 **return_intox_2_9** **Friendly**<br>10 **return_intox_2_10** **Relaxed**<br>11 **return_intox_2_11** **Diffculty concentrating**<br>12 **return_intox_2_12** **Feeling lightheaded or dizzy**<br>13 **return_intox_2_13** **Feeling confused**<br>0 **return_intox_2_0** **None of the above**
	992	**return_gi_2**<br><br>**Show the field ONLY if: [return_wd_2] = '1'**	**Section Header:** *Return GI 2*<br><br>**Which of the following gastrointestinal signs or symptoms were documented?**	**checkbox**<br>1 **return_gi_2_1** **Constipation**<br>2 **return_gi_2_2** **Turning of stomach/cramping/abdo pain**<br>3 **return_gi_2_3** **Nausea**<br>4 **return_gi_2_4** **Vomiting**<br>5 **return_gi_2_5** **Diarrhea**<br>0 **return_gi_2_0** **None of the above**
	993	**return_anxiety_2**<br><br>**Show the field ONLY if: [return_wd_2] = '1'**	**Section Header:** *Return Anxiety 2*<br><br>**Which of the following signs or symptoms of anxiety were documented?**	**checkbox**<br>1 **return_anxiety_2_1** **Anxiety, irritability, or agitation**<br>2 **return_anxiety_2_2** **Nervousness**<br>3 **return_anxiety_2_3** **Tremor**<br>4 **return_anxiety_2_4** **Restlessness**<br>0 **return_anxiety_2_0** **None of the above**

	994	**return_withdrawal_2**<br><br>**Show the field ONLY if: [return_wd_2] = '1'**	**Section Header:** *Return Withdrawal 2*<br><br>**Which of the following signs or symptoms of withdrawal were documented?**	**checkbox**<br>1 return_withdrawal_2_1 Yawning<br>2 return_withdrawal_2_2 Gooseflesh skin (chills/piloerecti<br>3 return_withdrawal_2_3 Pulse > 80<br>4 return_withdrawal_2_4 Sweating (diaphoresis)<br>5 return_withdrawal_2_5 Pupil size larger normal (dilation/mydra<br>6 return_withdrawal_2_6 Bone, joint ache body aches (arthralgias/mya<br>7 return_withdrawal_2_7 Runny nose or tearing (rhinor or lacrimation)<br>0 return_withdrawal_2_0 None of the abo
	995	**return_vitals_2**<br><br>**Show the field ONLY if: [return_wd_2] = '1'**	**Section Header:** *Return Abnormal Vitals 2*<br><br>**Which of the following abnormal vital signs were documented?**	**checkbox**<br>1 return_vitals_2_1 Hypoxia<br>2 return_vitals_2_2 Hypercarbia<br>3 return_vitals_2_3 Hypotension<br>4 return_vitals_2_4 Bradycardia<br>0 return_vitals_2_0 None of the above
	996	**return_misc_2**<br><br>**Show the field ONLY if: [return_wd_2] = '1'**	**Section Header:** *Return Miscellaneous 2*<br><br>**Which of the following miscellaneous opioid related side effects were documented?**	**checkbox**<br>1 return_misc_2_1 Diffculty passing urine<br>2 return_misc_2_2 Itchiness<br>3 return_misc_2_3 Dry mouth<br>4 return_misc_2_4 Headache<br>5 return_misc_2_5 Unable to walk<br>6 return_misc_2_6 Unable to discharge<br>7 return_misc_2_7 Pupil size smaller than normal (constriction/miosis)<br>0 return_misc_2_0 None of the above
	997	**datetime_return_3**<br><br>**Show the field ONLY if: [return_num] = '3' or [return_num] = '4' or [return_num] = '5' or [return_num] = '6' or [return_num] = '7'**	**Section Header:** *Third Return Visit*<br><br>**Date and time of arrival**	**text (datetime_mdy)**

	998	return_redcap_3<br>Show the field ONLY if: [return_num] = '3' or [return_num] = '4' or [return_num] = '5' or [return_num] = '6' or [return_num] = '7'	Does this encounter coincide with an existing visit in REDCap?	yesno<br>1 Yes<br>0 No
	999	return_redcap_spec_3<br>Show the field ONLY if: [return_redcap_3] = '1'	Please enter the visit number	text
	1000	return_od_3<br>Show the field ONLY if: [return_num] = '3' or [return_num] = '4' or [return_num] = '5' or [return_num] = '6' or [return_num] = '7'	Did the patient present to the ED for opioid overdose?	yesno<br>1 Yes<br>0 No
	1001	return_illicit_yn_3<br>Show the field ONLY if: [return_num] = '3' or [return_num] = '4' or [return_num] = '5' or [return_num] = '6' or [return_num] = '7'	Did the patient or provider report any substance use between the index ED visit and this return visit?	yesno<br>1 Yes<br>0 No
	1002	return_substance_3<br>Show the field ONLY if: [return_illicit_yn_3] = '1'	Which substance(s) did the patient use?	checkbox<br>1 return_substance_3 1 Opioids (not including buprenorph<br>2 return_substance_3 2 Methamphe<br>3 return_substance_3_ 3 Cocaine<br>4 return_substance_3 4 Cannabis<br>5 return_substance_3 5 Hallucinoge (including Ecstasy/MD<br>6 return_substance_3_ 6 LSD<br>7 return_substance_3 7 Benzodiaze<br>8 return_substance_3 8 Alcohol<br>9 return_substance_3_ 9 Tobacco<br>10 return_substance_3_ 10 Other stimu NOS<br>11 return_substance_3_ 11 Other (note heavy usage<br>999 return_substance_3_999 None of the
	1003	return_substance_oth_3<br>Show the field ONLY if: [return_substance_3(11)] = '1'	If "Other," please specify.	text

	**1004 return_opioid_3**<br>**Show the field ONLY if: [return_substance_3(1)] = '1'**	**Which opioid(s) did the patient use?**	**checkbox**<br>1 return_opioid_3_1 Heroin<br>2 return_opioid_3_2 Oxycodone (OxyContin, Percocet, etc.)<br>3 return_opioid_3_3 Hydrocodone (Norco, Vicodin, etc.)<br>4 return_opioid_3_4 Fentanyl (Duragesic, Ionsys)<br>5 return_opioid_3_5 Hydromorphone (Dilaudid)<br>6 return_opioid_3_6 Morphine<br>7 return_opioid_3_7 Other
	**1005 return_opioid_oth_3**<br>**Show the field ONLY if: [return_opioid_3(7)] = '1'**	**If "Other," please specify.**	**text**
	**1006 return_utox_3**<br>**Show the field ONLY if: [return_num] = '3' or [return_num] = '4' or [return_num] = '5' or [return_num] = '6' or [return_num] = '7'**	**Was a urine toxicology screen performed for this visit?**	**yesno**<br>1 Yes<br>0 No
	**1007 return_utox_spec_3**<br>**Show the field ONLY if: [return_utox_3] = '1'**	**Please select all drugs shown to be positive in the urine toxicology analysis:**	**checkbox**<br>1 return_utox_spec_3_1 Amphetami<br>2 return_utox_spec_3_2 Barbiturates<br>3 return_utox_spec_3_ 3 Cocaine<br>4 return_utox_spec_3_4 Methaqualo<br>5 return_utox_spec_3_5 Methadone<br>6 return_utox_spec_3_6 Methamphe<br>7 return_utox_spec_3_7 Opiates<br>8 return_utox_spec_3_8 Phencyclidin<br>9 return_utox_spec_3_9 Marijuana<br>10 return_utox_spec_3_ 10 Benzodiazep<br>11 return_utox_spec_3_ 11 Propoxyphe<br>12 return_utox_spec_3_ 12 Amphetami<br>13 return_utox_spec_3_ 13 Other<br>999 return_utox_spec_3 999 None of the
	**1008 return_utox_oth_3**<br>**Show the field ONLY if: [return_utox_spec_3(13)] = '1'**	**If "Other," please specify**	**text**

1009	return_craving_3<br><br>**Show the field ONLY if:** [return_num] = '3' or [return_num] = '4' or [return_num] = '5' or [return_num] = '6' or [return_num] = '7'	**Did the patient or provider mention opioid cravings during the visit?**	**yesno**<br>1 Yes<br>0 No
1010	return_wd_3<br><br>**Show the field ONLY if:** [return_num] = '3' or [return_num] = '4' or [return_num] = '5' or [return_num] = '6' or [return_num] = '7'	**Did the patient complaint about or exhibit any signs or symptoms of withdrawal during this visit?**<br>*If the patient received buprenorphine during this visit, only select signs/symptoms documented BEFORE buprenorphine administration.*	**yesno**<br>1 Yes<br>0 No
1011	return_sedation_3<br><br>**Show the field ONLY if:** [return_wd_3] = '1'	**Section Header:** *Return Sedation 3*<br><br>**Which of the following signs or symptoms of sedation were documented?**	**checkbox**<br>1 return_sedation_3_1 Nodding<br>2 return_sedation_3_2 Sleepy, drowsy, diffculty staying awake<br>3 return_sedation_3_3 Feelings of general fatigue or weakness<br>0 return_sedation_3_0 None of the above
1012	return_intox_3<br><br>**Show the field ONLY if:** [return_wd_3] = '1'	**Section Header:** *Return Intoxication 3*<br><br>**Which of the following signs or symptoms of intoxication were documented?**	**checkbox**<br>1 return_intox_3_1 Good mood<br>2 return_intox_3_2 Coasting (Spaced out)<br>3 return_intox_3_3 Pleasant Sick<br>4 return_intox_3_4 Drive (energy)<br>5 return_intox_3_5 High<br>6 return_intox_3_6 Drunken<br>7 return_intox_3_7 Soapbox (talkative)<br>8 return_intox_3_8 Rush<br>9 return_intox_3_9 Friendly<br>10 return_intox_3_10 Relaxed<br>11 return_intox_3_11 Diffculty concentrating<br>12 return_intox_3_12 Feeling lightheaded or dizzy<br>13 return_intox_3_13 Feeling confused<br>0 return_intox_3_0 None of the above

	1013	**return_gi_3**<br><br>**Show the field ONLY if:**<br>**[return_wd_3] ='1'**	**Section Header:** *Return GI 3*<br><br>**Which of the following gastrointestinal signs or symptoms were documented?**	**checkbox**<br>1 return_gi_3 1 Constipation<br>2 return_gi_3 2 Turning of stomach/cramping/abdo pain<br>3 return_gi_3 3 Nausea<br>4 return_gi_3_ 4 Vomiting<br>5 return_gi_3_ 5 Diarrhea<br>0 return_gi_3 0 None of the above
	1014	**return_anxiety_3**<br><br>**Show the field ONLY if:**<br>**[return_wd_3] ='1'**	**Section Header:** *Return Anxiety 3*<br><br>**Which of the following signs or symptoms of anxiety were documented?**	**checkbox**<br>1 return_anxiety_3_1 Anxiety, irritability, or agitation<br>2 return_anxiety_3_2 Nervousness<br>3 return_anxiety_3_3 Tremor<br>4 return_anxiety_3_4 Restlessness<br>0 return_anxiety_3_0 None of the above
	1015	**return_withdrawal_3**<br><br>**Show the field ONLY if:**<br>**[return_wd_3] ='1'**	**Section Header:** *Return Withdrawal 3*<br><br>**Which of the following signs or symptoms of withdrawal were documented?**	**checkbox**<br>1 return_withdrawal_3 1 Yawning<br>2 return_withdrawal_3 2 Gooseflesh skin (chills/piloerecti<br>3 return_withdrawal_3 3 Pulse > 80<br>4 return_withdrawal_3 4 Sweating (diaphoresis)<br>5 return_withdrawal_3 5 Pupil size larger normal (dilation/mydria<br>6 return_withdrawal_3 6 Bone, joint ache body aches (arthralgias/mya<br>7 return_withdrawal_3 7 Runny nose or tearing (rhinorr or lacrimation)<br>0 return_withdrawal_3 0 None of the abo
	1016	**return_vitals_3**<br><br>**Show the field ONLY if:**<br>**[return_wd_3] ='1'**	**Section Header:** *Return Abnormal Vitals 3*<br><br>**Which of the following abnormal vital signs were documented?**	**checkbox**<br>1 return_vitals_3_1 Hypoxia<br>2 return_vitals_3_2 Hypercarbia<br>3 return_vitals_3_3 Hypotension<br>4 return_vitals_3_4 Bradycardia<br>0 return_vitals_3_0 None of the above

	**1017 return_misc_3**<br>**Show the field ONLY if: [return_wd_3] ='1'**	**Section Header:** *Return Miscellaneous 3*<br>**Which of the following miscellaneous opioid related side effects were documented?**	**checkbox**<br>1 return_misc_3 1 Diffculty passing urine<br>2 return_misc_3_2 Itchiness<br>3 return_misc_3_3 Dry mouth<br>4 return_misc_3_4 Headache<br>5 return_misc_3_5 Unable to walk<br>6 return_misc_3_6 Unable to discharge<br>7 return_misc_3_7 Pupil size smaller than normal (constriction/miosis)<br>0 return_misc_3_0 None of the above
	**1018 datetime_return_4**<br>**Show the field ONLY if: [return_num] = '4' or [return_num] = '5' or [return_num] = '6' or [return_num] ='7'**	**Section Header:** *Fourth Return Visit*<br>**Date and time of arrival**	**text (datetime_mdy)**
	**1019 return_redcap_4**<br>**Show the field ONLY if: [return_num] = '4' or [return_num] = '5' or [return_num] = '6' or [return_num] ='7'**	**Does this encounter coincide with an existing visit in REDCap?**	**yesno**<br>1 Yes<br>0 No
	**1020 return_redcap_spec_4**<br>**Show the field ONLY if: [return_redcap_4] ='1'**	**Please enter the visit number**	**text**
	**1021 return_od_4**<br>**Show the field ONLY if: [return_num] = '4' or [return_num] = '5' or [return_num] = '6' or [return_num] ='7'**	**Did the patient present to the ED for opioid overdose?**	**yesno**<br>1 Yes<br>0 No
	**1022 return_illicit_yn_4**<br>**Show the field ONLY if: [return_num] = '4' or [return_num] = '5' or [return_num] = '6' or [return_num] ='7'**	**Did the patient or provider report any substance use between the index ED visit and this return visit?**	**yesno**<br>1 Yes<br>0 No

	1023 return_substance_4<br><br>Show the field ONLY if:<br>[return_illicit_yn_4] = '1'	Which substance(s) did the patient use?	checkbox<br>1 return_substance_4 1 Opioids (not including buprenorph<br>2 return_substance_4 2 Methamphe<br>3 return_substance_4_ 3 Cocaine<br>4 return_substance_4 4 Cannabis<br>5 return_substance_4 5 Hallucinoge (including Ecstasy/MD<br>6 return_substance_4_ 6 LSD<br>7 return_substance_4 7 Benzodiaze<br>8 return_substance_4 8 Alcohol<br>9 return_substance_4_ 9 Tobacco<br>10 return_substance_4_ 10 Other stimu NOS<br>11 return_substance_4_ 11 Other (note heavy usage<br>999 return_substance_4 999 None of the
	1024 return_substance_oth_4<br><br>Show the field ONLY if:<br>[return_substance_4(11)] = '1'	If "Other," please specify.	text
	1025 return_opioid_4<br><br>Show the field ONLY if:<br>[return_substance_4(1)] = '1'	Which opioid(s) did the patient use?	checkbox<br>1 return_opioid_4_1 Heroin<br>2 return_opioid_4_2 Oxycodone (OxyContin, Percocet, etc.)<br>3 return_opioid_4_3 Hydrocodone (Norco, Vicodin, etc.)<br>4 return_opioid_4_4 Fentanyl (Duragesic, Ionsys)<br>5 return_opioid_4_5 Hydromorphone (Dilaudid)<br>6 return_opioid_4_6 Morphine<br>7 return_opioid_4_7 Other
	1026 return_opioid_oth_4<br><br>Show the field ONLY if:<br>[return_opioid_4(7)] = '1'	If "Other," please specify.	text
	1027 return_utox_4<br><br>Show the field ONLY if:<br>[return_num] = '4' or [return_num] = '5' or [return_num] = '6' or [return_num] = '7'	Was a urine toxicology screen performed for this visit?	yesno<br>1 Yes<br>0 No

1028	return_utox_spec_4<br><br>Show the field ONLY if:<br>[return_utox_4] = '1'	Please select all drugs shown to be positive in the urine toxicology analysis:	checkbox<br>1 return_utox_spec_4 1 Amphetami<br>2 return_utox_spec_4 2 Barbiturates<br>3 return_utox_spec_4_ 3 Cocaine<br>4 return_utox_spec_4 4 Methaqualo<br>5 return_utox_spec_4 5 Methadone<br>6 return_utox_spec_4 6 Methamphe<br>7 return_utox_spec_4 7 Opiates<br>8 return_utox_spec_4 8 Phencyclidin<br>9 return_utox_spec_4 9 Marijuana<br>10 return_utox_spec_4_ 10 Benzodiazep<br>11 return_utox_spec_4_ 11 Propoxyphe<br>12 return_utox_spec_4_ 12 Amphetami<br>13 return_utox_spec_4_ 13 Other<br>999 return_utox_spec_4 999 None of the
1029	return_utox_oth_4<br><br>Show the field ONLY if:<br>[return_utox_spec_4(13)] = '1'	If "Other," please specify	text
1030	return_craving_4<br><br>Show the field ONLY if:<br>[return_num] = '4' or [return_num] = '5' or [return_num] = '6' or [return_num] = '7'	Did the patient or provider mention opioid cravings during the visit?	yesno<br>1 Yes<br>0 No
1031	return_wd_4<br><br>Show the field ONLY if:<br>[return_num] = '4' or [return_num] = '5' or [return_num] = '6' or [return_num] = '7'	Did the patient complaint about or exhibit any signs or symptoms of withdrawal during this visit?<br>*If the patient received buprenorphine during this visit, only select signs/symptoms documented BEFORE buprenorphine administration.*	yesno<br>1 Yes<br>0 No
1032	return_sedation_4<br><br>Show the field ONLY if:<br>[return_wd_4] = '1'	Section Header: *Return Sedation 4*<br><br>Which of the following signs or symptoms of sedation were documented?	checkbox<br>1 return_sedation_4_1 Nodding<br>2 return_sedation_4_2 Sleepy, drowsy, diffculty staying awake<br>3 return_sedation_4_3 Feelings of general fatigue or weakness<br>0 return_sedation_4_0 None of the above

	1033	return_intox_4<br><br>Show the field ONLY if:<br>[return_wd_4] ='1'	Section Header: *Return Intoxication 4*<br><br>Which of the following signs or symptoms of intoxication were documented?	checkbox<br>1 return_intox_4_1 Good mood<br>2 return_intox_4_2 Coasting (Spaced out)<br>3 return_intox_4_3 Pleasant Sick<br>4 return_intox_4_4 Drive (energy)<br>5 return_intox_4_5 High<br>6 return_intox_4_6 Drunken<br>7 return_intox_4_7 Soapbox (talkative)<br>8 return_intox_4_8 Rush<br>9 return_intox_4_9 Friendly<br>10 return_intox_4_10 Relaxed<br>11 return_intox_4_11 Diffculty concentrating<br>12 return_intox_4_12 Feeling lightheaded or dizzy<br>13 return_intox_4_13 Feeling confused<br>0 return_intox_4_0 None of the above
	1034	return_gi_4<br><br>Show the field ONLY if:<br>[return_wd_4] ='1'	Section Header: *Return GI 4*<br><br>Which of the following gastrointestinal signs or symptoms were documented?	checkbox<br>1 return_gi_4 1 Constipation<br>2 return_gi_4 2 Turning of stomach/cramping/abdo pain<br>3 return_gi_4 3 Nausea<br>4 return_gi_4_ 4 Vomiting<br>5 return_gi_4_ 5 Diarrhea<br>0 return_gi_4 0 None of the above
	1035	return_anxiety_4<br><br>Show the field ONLY if:<br>[return_wd_4] ='1'	Section Header: *Return Anxiety 4*<br><br>Which of the following signs or symptoms of anxiety were documented?	checkbox<br>1 return_anxiety_4_1 Anxiety, irritability, or agitation<br>2 return_anxiety_4_2 Nervousness<br>3 return_anxiety_4_3 Tremor<br>4 return_anxiety_4_4 Restlessness<br>0 return_anxiety_4_0 None of the above

	1036 return_withdrawal_4<br><br>Show the field ONLY if:<br>[return_wd_4] = '1'	**Section Header:** *Return Withdrawal 4*<br><br>Which of the following signs or symptoms of withdrawal were documented?	checkbox<br>1 return_withdrawal_4 1 Yawning<br>2 return_withdrawal_4 2 Gooseflesh skin (chills/piloerecti<br>3 return_withdrawal_4 3 Pulse > 80<br>4 return_withdrawal_4 4 Sweating (diaphoresis)<br>5 return_withdrawal_4 5 Pupil size larger normal (dilation/mydria<br>6 return_withdrawal_4 6 Bone, joint ache body aches (arthralgias/mya<br>7 return_withdrawal_4 7 Runny nose or tearing (rhinorr or lacrimation)<br>0 return_withdrawal_4 0 None of the abo
	1037 return_vitals_4<br><br>Show the field ONLY if:<br>[return_wd_4] = '1'	**Section Header:** *Return Vitals 4*<br><br>Which of the following abnormal vital signs were documented?	checkbox<br>1 return_vitals_4 1 Hypoxia<br>2 return_vitals_4 2 Hypercarbia<br>3 return_vitals_4 3 Hypotension<br>4 return_vitals_4 4 Bradycardia<br>0 return_vitals_4 0 None of the above
	1038 return_misc_4<br><br>Show the field ONLY if:<br>[return_wd_4] = '1'	**Section Header:** *Return Miscellaneous 4*<br><br>Which of the following miscellaneous opioid related side effects were documented?	checkbox<br>1 return_misc_4 1 Diffculty passing urine<br>2 return_misc_4 2 Itchiness<br>3 return_misc_4 3 Dry mouth<br>4 return_misc_4 4 Headache<br>5 return_misc_4 5 Unable to walk<br>6 return_misc_4 6 Unable to discharge<br>7 return_misc_4 7 Pupil size smaller than normal (constriction/miosis)<br>0 return_misc_4 0 None of the above
	1039 datetime_return_5<br><br>Show the field ONLY if:<br>[return_num] = '5' or [return_num] = '6' or [return_num] = '7'	**Section Header:** *Fifth Return Visit*<br><br>Date and time of arrival	text (datetime_mdy)
	1040 return_redcap_5<br><br>Show the field ONLY if:<br>[return_num] = '5' or [return_num] = '6' or [return_num] = '7'	Does this encounter coincide with an existing visit in REDCap?	yesno<br>1 Yes<br>0 No

	1041	return_redcap_spec_5<br>Show the field ONLY if:<br>[return_redcap_5] ='1'	Please enter the visit number	text
	1042	return_od_5<br>Show the field ONLY if:<br>[return_num] = '5' or [return_num] = '6' or [return_num] = '7'	Did the patient present to the ED for opioid overdose?	yesno<br>1 Yes<br>0 No
	1043	return_illicit_yn_5<br>Show the field ONLY if:<br>[return_num] = '5' or [return_num] = '6' or [return_num] = '7'	Did the patient or provider report any substance use between the index ED visit and this return visit?	yesno<br>1 Yes<br>0 No
	1044	return_substance_5<br>Show the field ONLY if:<br>[return_illicit_yn_5] = '1'	Which substance(s) did the patient use?	checkbox<br>1 return_substance_5 1 Opioids (not including buprenorph<br>2 return_substance_5 2 Methamphe<br>3 return_substance_5_ 3 Cocaine<br>4 return_substance_5 4 Cannabis<br>5 return_substance_5 5 Hallucinoge (including Ecstasy/MD<br>6 return_substance_5_ 6 LSD<br>7 return_substance_5 7 Benzodiaze<br>8 return_substance_5 8 Alcohol<br>9 return_substance_5_ 9 Tobacco<br>10 return_substance_5_ 10 Other stimu NOS<br>11 return_substance_5_ 11 Other (note heavy usage<br>999 return_substance_5_999 None of the
	1045	return_substance_oth_5<br>Show the field ONLY if:<br>[return_substance_5(11)] = '1'	If "Other," please specify.	text

1046	return_opioid_5<br>Show the field ONLY if: [return_substance_5(1)] = '1'	Which opioid(s) did the patient use?	checkbox<br>1 return_opioid_5_1 Heroin<br>2 return_opioid_5_2 Oxycodone (OxyContin, Percocet, etc.)<br>3 return_opioid_5_3 Hydrocodone (Norco, Vicodin, etc.)<br>4 return_opioid_5_4 Fentanyl (Duragesic, Ionsys)<br>5 return_opioid_5_5 Hydromorphone (Dilaudid)<br>6 return_opioid_5_6 Morphine<br>7 return_opioid_5_7 Other
1047	return_opioid_oth_5<br>Show the field ONLY if: [return_opioid_5(7)] = '1'	If "Other," please specify.	text
1048	return_utox_5<br>Show the field ONLY if: [return_num] = '5' or [return_num] = '6' or [return_num] = '7'	Was a urine toxicology screen performed for this visit?	yesno<br>1 Yes<br>0 No
1049	return_utox_spec_5<br>Show the field ONLY if: [return_utox_5] = '1'	Please select all drugs shown to be positive in the urine toxicology analysis:	checkbox<br>1 return_utox_spec_5_1 Amphetami<br>2 return_utox_spec_5_2 Barbiturates<br>3 return_utox_spec_5_3 Cocaine<br>4 return_utox_spec_5_4 Methaqualo<br>5 return_utox_spec_5_5 Methadone<br>6 return_utox_spec_5_6 Methamphe<br>7 return_utox_spec_5_7 Opiates<br>8 return_utox_spec_5_8 Phencyclidin<br>9 return_utox_spec_5_9 Marijuana<br>10 return_utox_spec_5_10 Benzodiazep<br>11 return_utox_spec_5_11 Propoxyphe<br>12 return_utox_spec_5_12 Amphetami<br>13 return_utox_spec_5_13 Other<br>999 return_utox_spec_5 999 None of the
1050	return_utox_oth_5<br>Show the field ONLY if: [return_utox_spec_5(13)] = '1'	If "Other," please specify	text

1051	return_craving_5<br><br>Show the field ONLY if: [return_num] = '5' or [return_num] = '6' or [return_num] = '7'	Did the patient or provider mention opioid cravings during the visit?	yesno<br>1 Yes<br>0 No
1052	return_wd_5<br><br>Show the field ONLY if: [return_num] = '5' or [return_num] = '6' or [return_num] = '7'	Did the patient complaint about or exhibit any signs or symptoms of withdrawal during this visit?<br>*If the patient received buprenorphine during this visit, only select signs/symptoms documented BEFORE buprenorphine administration.*	yesno<br>1 Yes<br>0 No
1053	return_sedation_5<br><br>Show the field ONLY if: [return_wd_5] = '1'	Section Header: *Return Sedation 5*<br><br>Which of the following signs or symptoms of sedation were documented?	checkbox<br>1 return_sedation_5_1 Nodding<br>2 return_sedation_5_2 Sleepy, drowsy, diffculty staying awake<br>3 return_sedation_5_3 Feelings of general fatigue or weakness<br>0 return_sedation_5_0 None of the above
1054	return_intox_5<br><br>Show the field ONLY if: [return_wd_5] = '1'	Section Header: *Return Intoxication 5*<br><br>Which of the following signs or symptoms of intoxication were documented?	checkbox<br>1 return_intox_5_1 Good mood<br>2 return_intox_5_2 Coasting (Spaced out)<br>3 return_intox_5_3 Pleasant Sick<br>4 return_intox_5_4 Drive (energy)<br>5 return_intox_5_5 High<br>6 return_intox_5_6 Drunken<br>7 return_intox_5_7 Soapbox (talkative)<br>8 return_intox_5_8 Rush<br>9 return_intox_5_9 Friendly<br>10 return_intox_5_10 Relaxed<br>11 return_intox_5_11 Diffculty concentrating<br>12 return_intox_5_12 Feeling lightheaded or dizzy<br>13 return_intox_5_13 Feeling confused<br>0 return_intox_5_0 None of the above

	1055	**return_gi_5**<br>**Show the field ONLY if: [return_wd_5] ='1'**	**Section Header:** *Return GI 5*<br>**Which of the following gastrointestinal signs or symptoms were documented?**	**checkbox**<br>1 return_gi_5 1 Constipation<br>2 return_gi_5 2 Turning of stomach/cramping/abdo pain<br>3 return_gi_5 3 Nausea<br>4 return_gi_5_ 4 Vomiting<br>5 return_gi_5_ 5 Diarrhea<br>0 return_gi_5 0 None of the above
	1056	**return_anxiety_5**<br>**Show the field ONLY if: [return_wd_5] ='1'**	**Section Header:** *Return Anxiety 5*<br>**Which of the following signs or symptoms of anxiety were documented?**	**checkbox**<br>1 return_anxiety_5_1 Anxiety, irritability, or agitation<br>2 return_anxiety_5_2 Nervousness<br>3 return_anxiety_5_3 Tremor<br>4 return_anxiety_5_4 Restlessness<br>0 return_anxiety_5_0 None of the above
	1057	**return_withdrawal_5**<br>**Show the field ONLY if: [return_wd_5] ='1'**	**Section Header:** *Return Withdrawal 5*<br>**Which of the following signs or symptoms of withdrawal were documented?**	**checkbox**<br>1 return_withdrawal_5 1 Yawning<br>2 return_withdrawal_5 2 Gooseflesh skin (chills/piloerecti<br>3 return_withdrawal_5 3 Pulse > 80<br>4 return_withdrawal_5 4 Sweating (diaphoresis)<br>5 return_withdrawal_5 5 Pupil size larger normal (dilation/mydria<br>6 return_withdrawal_5 6 Bone, joint ache body aches (arthralgias/mya<br>7 return_withdrawal_5 7 Runny nose or tearing (rhinorr or lacrimation)<br>0 return_withdrawal_5 0 None of the abo
	1058	**return_vitals_5**<br>**Show the field ONLY if: [return_wd_5] ='1'**	**Section Header:** *Return Abnormal Vitals 5*<br>**Which of the following abnormal vital signs were documented?**	**checkbox**<br>1 return_vitals_5_1 Hypoxia<br>2 return_vitals_5_2 Hypercarbia<br>3 return_vitals_5_3 Hypotension<br>4 return_vitals_5_4 Bradycardia<br>0 return_vitals_5_0 None of the above

	1059 return_misc_5<br><br>Show the field ONLY if: [return_wd_5] ='1'	Section Header: *Return Miscellaneous 5*<br><br>Which of the following miscellaneous opioid related side effects were documented?	checkbox<br>1 return_misc_5_1 Diffculty passing urine<br>2 return_misc_5_2 Itchiness<br>3 return_misc_5_3 Dry mouth<br>4 return_misc_5_4 Headache<br>5 return_misc_5_5 Unable to walk<br>6 return_misc_5_6 Unable to discharge<br>7 return_misc_5_7 Pupil size smaller than normal (constriction/miosis)<br>0 return_misc_5_0 None of the above
	1060 datetime_return_6<br><br>Show the field ONLY if: [return_num] = '6' or [return_num] = '7'	Section Header: *Sixth Return Visit*<br><br>Date and time of arrival	text (datetime_mdy)
	1061 return_redcap_6<br><br>Show the field ONLY if: [return_num] = '6' or [return_num] = '7'	Does this encounter coincide with an existing visit in REDCap?	yesno<br>1 Yes<br>0 No
	1062 return_redcap_spec_6<br><br>Show the field ONLY if: [return_redcap_6] ='1'	Please enter the visit number	text
	1063 return_od_6<br><br>Show the field ONLY if: [return_num] = '6' or [return_num] = '7'	Did the patient present to the ED for opioid overdose?	yesno<br>1 Yes<br>0 No
	1064 return_illicit_yn_6<br><br>Show the field ONLY if: [return_num] = '6' or [return_num] = '7'	Did the patient or provider report any substance use between the index ED visit and this return visit?	yesno<br>1 Yes<br>0 No

	1065	**return_substance_6**<br>**Show the field ONLY if: [return_illicit_yn_6] = '1'**	**Which substance(s) did the patient use?**	**checkbox**<br>1 | return_substance_6 1 | Opioids (not including buprenorph<br>2 | return_substance_6 2 | Methamphe<br>3 | return_substance_6_ 3 | Cocaine<br>4 | return_substance_6 4 | Cannabis<br>5 | return_substance_6 5 | Hallucinoge (including Ecstasy/MD<br>6 | return_substance_6_ 6 | LSD<br>7 | return_substance_6 7 | Benzodiaze<br>8 | return_substance_6 8 | Alcohol<br>9 | return_substance_6_ 9 | Tobacco<br>10 | return_substance_6_ 10 | Other stimu NOS<br>11 | return_substance_6_ 11 | Other (note heavy usage<br>999 | return_substance_6 999 | None of the
	1066	**return_substance_oth_6**<br>**Show the field ONLY if: [return_substance_6(11)] = '1'**	**If "Other," please specify.**	**text**
	1067	**return_opioid_6**<br>**Show the field ONLY if: [return_substance_6(1)] = '1'**	**Which opioid(s) did the patient use?**	**checkbox**<br>1 | return_opioid_6_1 | Heroin<br>2 | return_opioid_6_2 | Oxycodone (OxyContin, Percocet, etc.)<br>3 | return_opioid_6_3 | Hydrocodone (Norco, Vicodin, etc.)<br>4 | return_opioid_6_4 | Fentanyl (Duragesic, Ionsys)<br>5 | return_opioid_6_5 | Hydromorphone (Dilaudid)<br>6 | return_opioid_6_6 | Morphine<br>7 | return_opioid_6_7 | Other
	1068	**return_opioid_oth_6**<br>**Show the field ONLY if: [return_opioid_6(7)] = '1'**	**If "Other," please specify.**	**text**
	1069	**return_utox_6**<br>**Show the field ONLY if: [return_num] = '6' or [return_num] = '7'**	**Was a urine toxicology screen performed for this visit?**	**yesno**<br>1 | Yes<br>0 | No

	1070	return_utox_spec_6<br><br>Show the field ONLY if:<br>[return_utox_6] = '1'	Please select all drugs shown to be positive in the urine toxicology analysis:	checkbox<br>1 return_utox_spec_6 1 Amphetami<br>2 return_utox_spec_6 2 Barbiturates<br>3 return_utox_spec_6_ 3 Cocaine<br>4 return_utox_spec_6 4 Methaqualo<br>5 return_utox_spec_6 5 Methadone<br>6 return_utox_spec_6 6 Methamphe<br>7 return_utox_spec_6 7 Opiates<br>8 return_utox_spec_6 8 Phencyclidin<br>9 return_utox_spec_6 9 Marijuana<br>10 return_utox_spec_6_ 10 Benzodiazep<br>11 return_utox_spec_6_ 11 Propoxyphe<br>12 return_utox_spec_6_ 12 Amphetami<br>13 return_utox_spec_6_ 13 Other<br>999 return_utox_spec_6 999 None of the
	1071	return_utox_oth_6<br><br>Show the field ONLY if:<br>[return_utox_spec_6(13)] = '1'	If "Other," please specify	text
	1072	return_craving_6<br><br>Show the field ONLY if:<br>[return_num] = '6' or [return_num] = '7'	Did the patient or provider mention opioid cravings during the visit?	yesno<br>1 Yes<br>0 No
	1073	return_wd_6<br><br>Show the field ONLY if:<br>[return_num] = '6' or [return_num] = '7'	Did the patient complaint about or exhibit any signs or symptoms of withdrawal during this visit?<br>*If the patient received buprenorphine during this visit, only select signs/symptoms documented BEFORE buprenorphine administration.*	yesno<br>1 Yes<br>0 No
	1074	return_sedation_6<br><br>Show the field ONLY if:<br>[return_wd_6] = '1'	Section Header: *Return Sedation 6*<br><br>Which of the following signs or symptoms of sedation were documented?	checkbox<br>1 return_sedation_6_1 Nodding<br>2 return_sedation_6_2 Sleepy, drowsy, diffculty staying awake<br>3 return_sedation_6_3 Feelings of general fatigue or weakness<br>0 return_sedation_6_0 None of the above

	1075	return_intox_6<br><br>Show the field ONLY if:<br>[return_wd_6] ='1'	Section Header: *Return Intoxication 6*<br><br>**Which of the following signs or symptoms of intoxication were documented?**	checkbox<br>1 return_intox_6_1 Good mood<br>2 return_intox_6_2 Coasting (Spaced out)<br>3 return_intox_6_3 Pleasant Sick<br>4 return_intox_6_4 Drive (energy)<br>5 return_intox_6_5 High<br>6 return_intox_6_6 Drunken<br>7 return_intox_6_7 Soapbox (talkative)<br>8 return_intox_6_8 Rush<br>9 return_intox_6_9 Friendly<br>10 return_intox_6_10 Relaxed<br>11 return_intox_6_11 Diffculty concentrating<br>12 return_intox_6_12 Feeling lightheaded or dizzy<br>13 return_intox_6_13 Feeling confused<br>0 return_intox_6_0 None of the above
	1076	return_gi_6<br><br>Show the field ONLY if:<br>[return_wd_6] ='1'	Section Header: *Return GI 6*<br><br>**Which of the following gastrointestinal signs or symptoms were documented?**	checkbox<br>1 return_gi_6 1 Constipation<br>2 return_gi_6 2 Turning of stomach/cramping/abdo pain<br>3 return_gi_6 3 Nausea<br>4 return_gi_6_ 4 Vomiting<br>5 return_gi_6_ 5 Diarrhea<br>0 return_gi_6 0 None of the above
	1077	return_anxiety_6<br><br>Show the field ONLY if:<br>[return_wd_6] ='1'	Section Header: *Return Anxiety 6*<br><br>**Which of the following signs or symptoms of anxiety were documented?**	checkbox<br>1 return_anxiety_6_1 Anxiety, irritability, or agitation<br>2 return_anxiety_6_2 Nervousness<br>3 return_anxiety_6_3 Tremor<br>4 return_anxiety_6_4 Restlessness<br>0 return_anxiety_6_0 None of the above

	1078 return_withdrawal_6<br><br>Show the field ONLY if:<br>[return_wd_6] ='1'	Section Header: *Return Withdrawal 6*<br><br>Which of the following signs or symptoms of withdrawal were documented?	checkbox<br><br>1 return_withdrawal_6 1 Yawning<br>2 return_withdrawal_6 2 Gooseflesh skin (chills/piloerecti<br>3 return_withdrawal_6 3 Pulse > 80<br>4 return_withdrawal_6 4 Sweating (diaphoresis)<br>5 return_withdrawal_6 5 Pupil size larger normal (dilation/mydria<br>6 return_withdrawal_6 6 Bone, joint ache body aches (arthralgias/mya<br>7 return_withdrawal_6 7 Runny nose or tearing (rhinorr or lacrimation)<br>0 return_withdrawal_6 0 None of the abo
	1079 return_vitals_6<br><br>Show the field ONLY if:<br>[return_wd_6] ='1'	Section Header: *Return Abnormal Vitals 6*<br><br>Which of the following abnormal vital signs were documented?	checkbox<br><br>1 return_vitals_6_1 Hypoxia<br>2 return_vitals_6_2 Hypercarbia<br>3 return_vitals_6_3 Hypotension<br>4 return_vitals_6_4 Bradycardia<br>0 return_vitals_6_0 None of the above
	1080 return_misc_6<br><br>Show the field ONLY if:<br>[return_wd_6] ='1'	Section Header: *Return Miscellaneous 6*<br><br>Which of the following miscellaneous opioid related side effects were documented?	checkbox<br><br>1 return_misc_6 1 Diffculty passing urine<br>2 return_misc_6 2 Itchiness<br>3 return_misc_6 3 Dry mouth<br>4 return_misc_6 4 Headache<br>5 return_misc_6 5 Unable to walk<br>6 return_misc_6 6 Unable to discharge<br>7 return_misc_6 7 Pupil size smaller than normal (constriction/miosis)<br>0 return_misc_6 0 None of the above
	1081 datetime_return_7<br><br>Show the field ONLY if:<br>[return_num] = '7'	Section Header: *Seventh Return Visit*<br><br>Date and time of arrival	text (datetime_mdy)
	1082 return_redcap_7<br><br>Show the field ONLY if:<br>[return_num] = '7'	Does this encounter coincide with an existing visit in REDCap?	yesno<br><br>1 Yes<br>0 No
	1083 return_redcap_spec_7<br><br>Show the field ONLY if:<br>[return_redcap_7] ='1'	Please enter the visit number	text

© 2021 Herring AA et al. *JAMA Network Open.*

1084	return_od_7<br>Show the field ONLY if:<br>[return_num] = '7'	Did the patient present to the ED for opioid overdose?	yesno<br>1 Yes<br>0 No
1085	return_illicit_yn_7<br>Show the field ONLY if:<br>[return_num] = '7'	Did the patient or provider report any substance use between the index ED visit and this return visit?	yesno<br>1 Yes<br>0 No
1086	return_substance_7<br>Show the field ONLY if:<br>[return_illicit_yn_7] = '1'	Which substance(s) did the patient use?	checkbox<br>1 return_substance_7 1 Opioids (not including buprenorph<br>2 return_substance_7 2 Methamphe<br>3 return_substance_7_ 3 Cocaine<br>4 return_substance_7 4 Cannabis<br>5 return_substance_7 5 Hallucinoge (including Ecstasy/MD<br>6 return_substance_7_ 6 LSD<br>7 return_substance_7 7 Benzodiaze<br>8 return_substance_7 8 Alcohol<br>9 return_substance_7_ 9 Tobacco<br>10 return_substance_7_ 10 Other stimu NOS<br>11 return_substance_7_ 11 Other (note heavy usage<br>999 return_substance_7_999 None of the
1087	return_substance_oth_7<br>Show the field ONLY if:<br>[return_substance_7(1 1)] = '1'	If "Other," please specify.	text
1088	return_opioid_7<br>Show the field ONLY if:<br>[return_substance_7(1)] = '1'	Which opioid(s) did the patient use?	checkbox<br>1 return_opioid_7_1 Heroin<br>2 return_opioid_7_2 Oxycodone (OxyContin, Percocet, etc.)<br>3 return_opioid_7_3 Hydrocodone (Norco, Vicodin, etc.)<br>4 return_opioid_7_4 Fentanyl (Duragesic, Ionsys)<br>5 return_opioid_7_5 Hydromorphone (Dilaudid)<br>6 return_opioid_7_6 Morphine<br>7 return_opioid_7_7 Other

	1089	return_opioid_oth_7<br>Show the field ONLY if: [return_opioid_7(7)] = '1'	If "Other," please specify.	text
	1090	return_utox_7<br>Show the field ONLY if: [return_num] = '7'	Was a urine toxicology screen performed for this visit?	yesno<br>1 Yes<br>0 No
	1091	return_utox_spec_7<br>Show the field ONLY if: [return_utox_7] = '1'	Please select all drugs shown to be positive in the urine toxicology analysis:	checkbox<br>1 return_utox_spec_7 1 Amphetami<br>2 return_utox_spec_7 2 Barbiturates<br>3 return_utox_spec_7_ 3 Cocaine<br>4 return_utox_spec_7 4 Methaqualo<br>5 return_utox_spec_7 5 Methadone<br>6 return_utox_spec_7 6 Methamphe<br>7 return_utox_spec_7 7 Opiates<br>8 return_utox_spec_7 8 Phencyclidin<br>9 return_utox_spec_7 9 Marijuana<br>10 return_utox_spec_7_ 10 Benzodiazep<br>11 return_utox_spec_7_ 11 Propoxyphe<br>12 return_utox_spec_7_ 12 Amphetami<br>13 return_utox_spec_7_ 13 Other<br>999 return_utox_spec_7 999 None of the
	1092	return_utox_oth_7<br>Show the field ONLY if: [return_utox_spec_7(13)] = '1'	If "Other," please specify	text
	1093	return_craving_7<br>Show the field ONLY if: [return_num] = '7'	Did the patient or provider mention opioid cravings during the visit?	yesno<br>1 Yes<br>0 No
	1094	return_wd_7<br>Show the field ONLY if: [return_num] = '7'	Did the patient complaint about or exhibit any signs or symptoms of withdrawal during this visit?<br>*If the patient received buprenorphine during this visit, only select signs/symptoms documented BEFORE buprenorphine administration.*	yesno<br>1 Yes<br>0 No
	1095	return_sedation_7<br>Show the field ONLY if: [return_wd_7] = '1'	**Section Header:** *Return Sedation 7*<br>Which of the following signs or symptoms of sedation were documented?	checkbox<br>1 return_sedation_7_1 Nodding<br>2 return_sedation_7_2 Sleepy, drowsy, diffculty staying awake<br>3 return_sedation_7_3 Feelings of general fatigue or weakness<br>0 return_sedation_7_0 None of the above

	1096	return_intox_7<br><br>Show the field ONLY if:<br>[return_wd_7] ='1'	Section Header: *Return Intoxication 7*<br><br>Which of the following signs or symptoms of intoxication were documented?	checkbox<br>1 return_intox_7_1 Good mood<br>2 return_intox_7_2 Coasting (Spaced out)<br>3 return_intox_7_3 Pleasant Sick<br>4 return_intox_7_4 Drive (energy)<br>5 return_intox_7_5 High<br>6 return_intox_7_6 Drunken<br>7 return_intox_7_7 Soapbox (talkative)<br>8 return_intox_7_8 Rush<br>9 return_intox_7_9 Friendly<br>10 return_intox_7_10 Relaxed<br>11 return_intox_7_11 Diffculty concentrating<br>12 return_intox_7_12 Feeling lightheaded or dizzy<br>13 return_intox_7_13 Feeling confused<br>0 return_intox_7_0 None of the above
	1097	return_gi_7<br><br>Show the field ONLY if:<br>[return_wd_7] ='1'	Section Header: *Return GI 7*<br><br>Which of the following gastrointestinal signs or symptoms were documented?	checkbox<br>1 return_gi_7 1 Constipation<br>2 return_gi_7 2 Turning of stomach/cramping/abdo pain<br>3 return_gi_7 3 Nausea<br>4 return_gi_7_ 4 Vomiting<br>5 return_gi_7_ 5 Diarrhea<br>0 return_gi_7 0 None of the above
	1098	return_anxiety_7<br><br>Show the field ONLY if:<br>[return_wd_7] ='1'	Section Header: *Return Anxiety 7*<br><br>Which of the following signs or symptoms of anxiety were documented?	checkbox<br>1 return_anxiety_7_1 Anxiety, irritability, or agitation<br>2 return_anxiety_7_2 Nervousness<br>3 return_anxiety_7_3 Tremor<br>4 return_anxiety_7_4 Restlessness<br>0 return_anxiety_7_0 None of the above

	1099	return_withdrawal_7<br><br>Show the field ONLY if:<br>[return_wd_7] ='1'	**Section Header:** *Return Withdrawal 7*<br><br>Which of the following signs or symptoms of withdrawal were documented?	checkbox<br><br>1 | return_withdrawal_7 1 | Yawning<br>2 | return_withdrawal_7 2 | Gooseflesh skin (chills/piloerecti<br>3 | return_withdrawal_7 3 | Pulse > 80<br>4 | return_withdrawal_7 4 | Sweating (diaphoresis)<br>5 | return_withdrawal_7 5 | Pupil size larger normal (dilation/mydria<br>6 | return_withdrawal_7 6 | Bone, joint ache body aches (arthralgias/mya<br>7 | return_withdrawal_7 7 | Runny nose or tearing (rhinorr or lacrimation)<br>0 | return_withdrawal_7 0 | None of the abo
	1100	return_vitals_7<br><br>Show the field ONLY if:<br>[return_wd_7] ='1'	**Section Header:** *Return Abnormal Vitals 7*<br><br>Which of the following abnormal vital signs were documented?	checkbox<br><br>1 | return_vitals_7_1 | Hypoxia<br>2 | return_vitals_7_2 | Hypercarbia<br>3 | return_vitals_7_3 | Hypotension<br>4 | return_vitals_7_4 | Bradycardia<br>0 | return_vitals_7_0 | None of the above
	1101	return_misc_7<br><br>Show the field ONLY if:<br>[return_wd_7] ='1'	**Section Header:** *Return Miscellaneous 7*<br><br>Which of the following miscellaneous opioid related side effects were documented?	checkbox<br><br>1 | return_misc_7 1 | Diffculty passing urine<br>2 | return_misc_7 2 | Itchiness<br>3 | return_misc_7 3 | Dry mouth<br>4 | return_misc_7 4 | Headache<br>5 | return_misc_7 5 | Unable to walk<br>6 | return_misc_7 6 | Unable to discharge<br>7 | return_misc_7 7 | Pupil size smaller than normal (constriction/miosis)<br>0 | return_misc_7 0 | None of the above
	1102	return_visits_complete	**Section Header:** *Form Status*<br><br>Complete?	dropdown<br><br>0 | Incomplete<br>1 | Unverified<br>2 | Complete
